# A Post-Synaptic Scaffold at the Origin of the Animal Kingdom

**DOI:** 10.1371/journal.pone.0000506

**Published:** 2007-06-06

**Authors:** Onur Sakarya, Kathryn A. Armstrong, Maja Adamska, Marcin Adamski, I-Fan Wang, Bruce Tidor, Bernard M. Degnan, Todd H. Oakley, Kenneth S. Kosik

**Affiliations:** 1 Neuroscience Research Institute, University of California, Santa Barbara, Santa Barbara, California, United States of America; 2 Department of Molecular, Cellular and Developmental Biology, University of California, Santa Barbara, Santa Barbara, California, United States of America; 3 Department of Computer Science, University of California, Santa Barbara, Santa Barbara, California, United States of America; 4 Biological Engineering Division, Computer Science and Artificial Intelligence Laboratory, Massachusetts Institute of Technology, Cambridge, Massachusetts, United States of America; 5 School of Integrative Biology, University of Queensland, Brisbane, Queensland, Australia; 6 Department of Ecology, Evolution and Marine Biology, University of California, Santa Barbara, Santa Barbara, California, United States of America; The Rockefeller University, United States of America

## Abstract

**Background:**

The evolution of complex sub-cellular structures such as the synapse requires the assembly of multiple proteins, each conferring added functionality to the integrated structure. Tracking the early evolution of synapses has not been possible without genomic information from the earliest branching animals. As the closest extant relatives to the Eumetazoa, Porifera (sponges) represent a pivotal group for understanding the evolution of nervous systems, because sponges lack neurons with clearly recognizable synapses, in contrast to eumetazoan animals.

**Methodology/Principal Findings:**

We show that the genome of the demosponge *Amphimedon queenslandica* possesses a nearly complete set of post-synaptic protein homologs whose conserved interaction motifs suggest assembly into a complex structure. In the critical synaptic scaffold gene, *dlg*, residues that make hydrogen bonds and van der Waals interactions with the PDZ ligand are 100% conserved between sponge and human, as is the motif organization of the scaffolds. Expression in *Amphimedon* of multiple post-synaptic gene homologs in larval flask cells further supports the existence of an assembled structure. Among the few post-synaptic genes absent from *Amphimedon*, but present in Eumetazoa, are receptor genes including the entire ionotropic glutamate receptor family.

**Conclusions/Significance:**

Highly conserved protein interaction motifs and co-expression in sponges of multiple proteins whose homologs interact in eumetazoan synapses indicate that a complex protein scaffold was present at the origin of animals, perhaps predating nervous systems. A relatively small number of crucial innovations to this pre-existing structure may represent the founding changes that led to a post-synaptic element.

## Introduction

A fundamental question in biology is how complex, integrated traits like nervous systems originated. One approach to this question involves analyzing individually the numerous components of a defining structure, such as the post-synaptic proteins of nervous systems, to determine which – if any – predate an integrated nervous system. These exapted [1] components, defined as a biologic unit originating with a function other than that for which it was later selected, can be revealed by comparative genomics of animals with nervous systems and their closest relatives that lack them. Furthermore, integration of the exapted components as an assembled structure may be inferred by the conservation of protein-protein interaction motifs between proteins of modern nervous systems and those of early branching animals that lack nervous systems.

Porifera (sponges) lack organs and nervous systems and possess a limited number of discrete cell types [2]. They lie in one of at least two highly informative phylogenetic positions with regard to the last common ancestor of all animals. One phylogenetic hypothesis, favored mostly by mitochondrial genome data, but known in other taxa to produce a misleading phylogenetic signal [3], is that sponges and Cnidarians form part of a monophyletic group sister to Bilaterians [4], [5]. Under a second phylogenetic hypothesis supported by a large amount of molecular and morphological evidence, the siliceous (demosponges + hexactinellids) and calcareous sponge lineages are the earliest branches off the main metazoan tree [6]–[8]. Under either phylogenetic hypothesis, shared features between sponges and bilaterians likely represent features of ancestral animals. Under the second phylogenetic hypothesis, sponge genomes additionally can reveal shared features that predate nervous systems and cell-specific adaptations, such as the synaptic junction.

A core structure of the post-synaptic complex is the post-synaptic density, a membrane region specialized for signaling and plasticity. While a definitive proteomic analysis of post-synaptic components does not exist, the enumerated proteins range from 77 to ∼1000 [9]–[11], boundaries which probably represent under- and over-estimates. These reports describe multiple isoforms of receptors, channels, adaptors, scaffolds, and proteins involved in adhesion, signaling, translation, and the cytoskeleton. Orthologous family members of proteins found in the post-synaptic complex are present in many animals; thus one can track this gene set over evolutionary time. Critical to the assembly of these proteins into a functional structure are protein-protein interaction domains. Among synaptic post-synaptic proteins the PDZ domain (**p**ost synaptic density protein (PSD95), **D**rosophila disc large tumor suppressor (*dlg*), and **z**o-1 protein) is a highly versatile interaction motif that connects many of the proteins in the post-synaptic junction. PDZ domains consist of 80–90 amino-acids and proteins containing these domains are found in bacteria, yeast, plants, and animals.

Recently, genomes from two representative organisms at pivotal positions in nervous system evolution have become available. They are the demosponge *Amphimedon queenslandica* (formerly known as *Reniera* sp.), which lacks neurons and a nervous system, and the cnidarian *Nematostella vectensis*, which possesses a nerve net that has condensed at some locations into plexuses and nerve tracts [12].

## Results

### Post-synaptic Genes in Early Branching Animals

**Figure 1:**
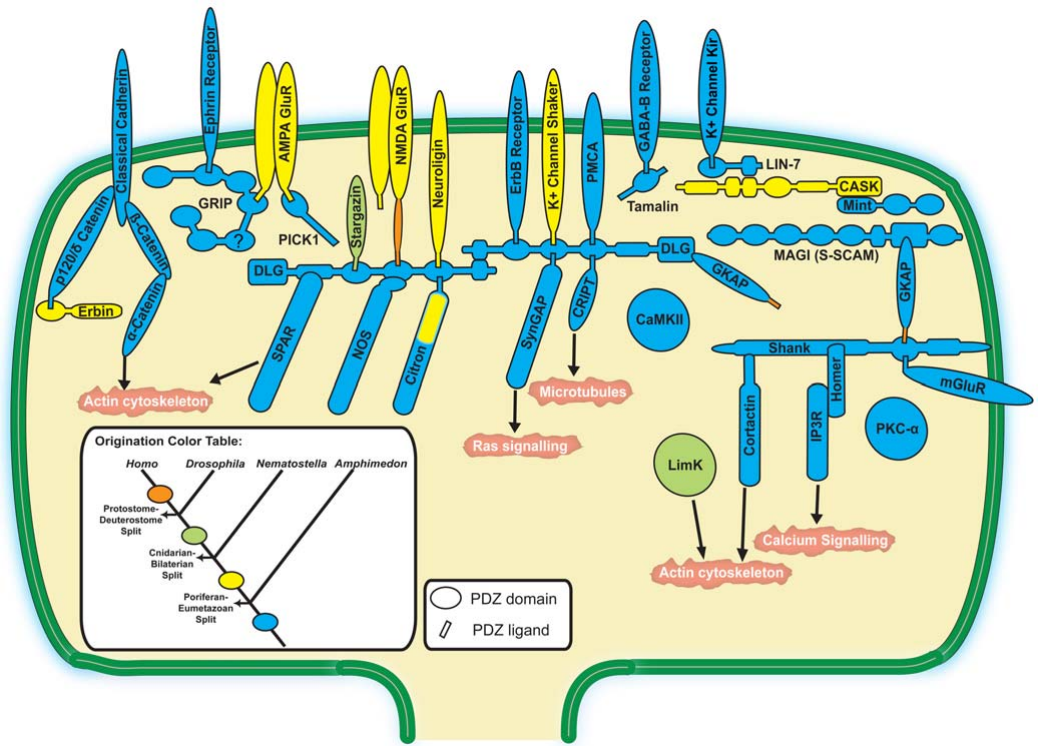
Origination periods of post-synaptic genes. One possible configuration of the post-synaptic genes based on the known organization of the post-synaptic junction is illustrated. Each color represents the origination period (figure inset) of the gene family inferred from phylogenetic analyses (Figure S1). As further evidence for orthology, domain architectures of selected gene family members were compared (Figure S2). NCBI accession numbers for each gene family member are provided in Table S1. Some gene families may have been lost from the investigated genomes and originated with an earlier ancestor than shown. Question mark indicates insufficient traces to confirm this PDZ domain.

We assembled phylogenies for 36 gene families of the post-synaptic excitatory vertebrate complex (Figure S1). The genomes of two basal metazoans were surveyed, *Amphimedon queenslandica* and *Nematostella vectensis*, as well as genes from *Drosophila melanogaster*, as a representative protostome, and *Homo sapiens*, as a representative deuterostome. A surprisingly large number of vertebrate post-synaptic gene homologs are present in the sponge and nearly the entire gene set is present in *Nematostella* (Figure 1). Furthermore, the domain architecture of all those proteins is highly conserved in the investigated animal lineages (Figure S2). Based on conservation of established binding domains, this set of genes appears capable of assembling their products into a complete sub-synaptic scaffold layer. For example, the *dlg* gene is present as a single copy and includes an L27 domain that has been lost from mammalian *dlg* paralogs except SAP-97. Conservation among PDZ ligand sequences is displayed in Table 1. For comparisons of the intron-exon structure of sponge *dlg* gene with human SAP-97, see Table S2. As shown in Table S3, most of these post-synaptic families do not have orthologs in yeast (*Saccharomyces cerevisiae*), dicty (*Dictyostelium discoideum*) and plants (*Arabidopsis thaliana* and *Oryza sativa*).

**Table 1:** PDZ ligand conservation in animal kingdom.

Species	δ-catenin	PMCA	CRIPT	GABABR	mGluR	K+ Kir	EphrinR	ErbBR
*Human*	∼PDSWV	∼LETSV	∼KQTSV	∼MVSGL	∼TTSSL	∼SESKV	∼VGIPI	∼EFIGA
		∼LETSL			∼SSSSL	∼NESKV	∼TGIQV	∼RNTVV
		∼VETSL			∼SSSTL	∼ARGSV	∼RMVPV	∼LDVPV
*Fly*	∼VRKQL	∼TETAV	∼KQSST	∼IVEYL	∼LQTNL	∼IDSIC	∼LDTII	∼TETRV
*Worm*	∼DDSWV	∼ETNNL	∼RQSTT	∼DEILL	∼HDTFL	∼ASGFL	∼EGFFV	∼KETCL
*Nematostella*	∼DFHAV	∼IETAM	∼RQSSA	∼YVIRL	∼ISTYL	∼DILFV	∼GELAI	N/A
*Sponge*	∼IDSWV	∼KETEV	∼VQSTV	∼EYYCV	∼NSTKL	∼EATNM	∼SPDFI	∼ATSIA

Sequences Correspond to the C-terminal 5 residues of the selected members of the gene families. N/A: gene not available. Species abbreviations used: Human, *Homo sapiens*; Fly, *Drosophila Melanogaster*; Worm, *Caenorhabditis elegans*; Nematostella, *Nematostella vectensis*; Sponge, *Amphimedon queenslandica*.

The main distinction among the post-synaptic gene set in the poriferan and cnidarian species are genes that encode excitatory post-synaptic receptors. Among the post-synaptic receptor genes absent from the *Amphimedon* and non-animal eukaryotic genomes such as yeast, *Tetrahymena*, and *Dictyostelium* are the Shaker type voltage gated K+ channel, neuroligin, and iGluRs (ionotropic glutamate receptors consisting of NMDA/AMPA/Kainate and Delta receptors). Orthologs of these genes are found in *Nematostella*. In the case of the iGluRs, *Nematostella* has a diversity of receptor subtypes comparable to human. At least 11 iGluRs are present in *Nematostella* compared to 18 in human and 20 to 30 in fly [13]. Of the 11 *Nematostella* iGluRs, eight clade with NMDA receptors and the remaining ones clade with AMPA/Kainate receptors (Figure S1.19). Multiple metabotropic glutamate receptors (mGluRs) are present in *Amphimedon,* but neither in *Amphimedon*, nor in *Nematostella* do they have sufficiently distinct sequence identities to be classified as members of bilaterian groups I, II, or III mGluRs (Figure S1.12). mGluR scaffolding proteins (Homer, Shank, PICK1) are also present in *Amphimedon*.

### Atomic Level Conservation of the Binding Domain

To investigate conservation of the binding domain at the atomic level we compared the PDZ3–CRIPT interaction in *Rattus norvegius* (rat), *Danio renio* (fish), *Drosophila melanogaster* (fly), *Anopheles gambiae* (mosquito), *Strongylocentrotus purpuratus* (sea urchin), *Caenorhabditis elegans* (worm), *Nematostella vectensis* (cnidarian), and *Amphimedon queenslandica* (sponge) (Figure S3). We used the rat PDZ3–CRIPT co-crystal structure (PDB code 1BE9 [14], 1.82-Å resolution), as the starting point for our analysis of this protein–protein complex. The rat and *Amphimedon* sequences are only 50% identical (over the residues in the 1BE9 structure that are designated as the PDZ3 domain, which are residues 302–402 in 1BE9), while the average identity between the rat and each of the other organisms is 66%. Despite these differences over the entire domain, the core residues in direct contact with the peptide are identical between the rat and sponge (Figure S4).

**Figure 2:**
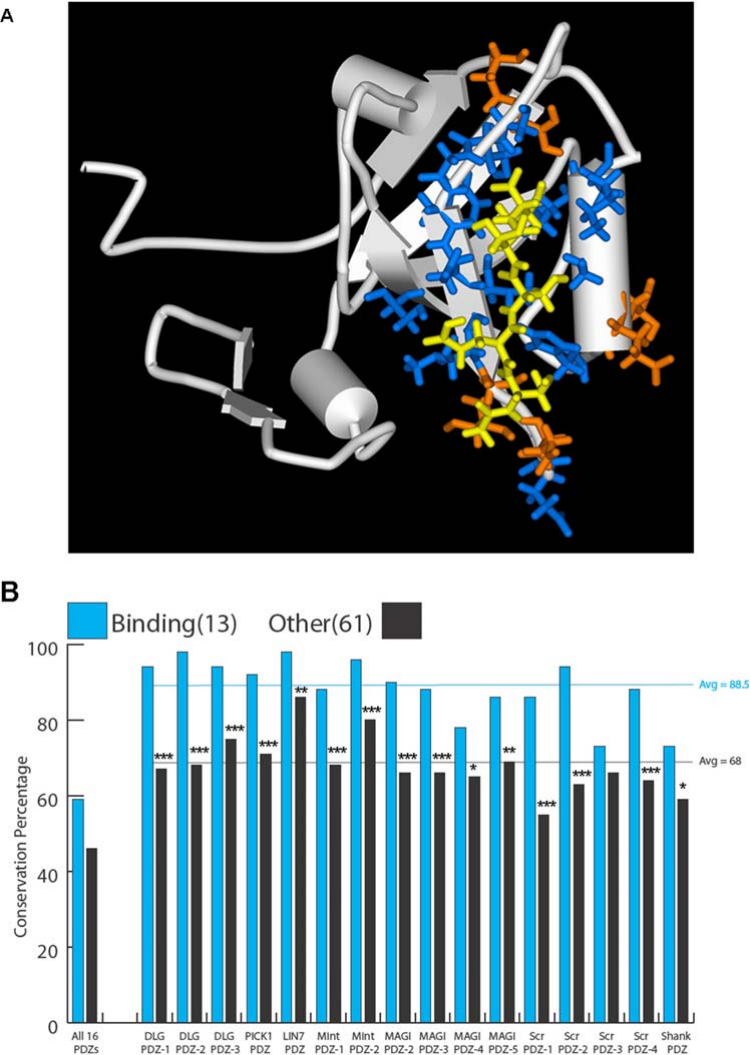
(A) Sponge PDZ3—CRIPT Homology Model. The last five residues of the CRIPT protein (yellow) interact with PDZ3 residues (blue and orange) by making van der Waals contacts, hydrogen bonds, or electrostatic interactions of greater than 0.1 kcal/mol in magnitude in any of the PDZ3 homology models. (Figure S4). The subset of residues painted blue represent the core union set that interact directly with the ligand in the PDZ1 co-crystal (2I1N), the PDZ2 co-crystal (2G2L), or the PDZ3 co-crystal (1BE9) by either van der Waals contacts of 3.9 Å or shorter or by hydrogen-bond lengths of 3.5 Å or shorter. (B) Ligand-binding residues are very highly conserved within a specific type of PDZ domain. Conservation of the 13 binding residues compared to the remaining 61 more distant residues for 16 types of PDZ domains from *Homo, Drosophila, Nematostella* and *Amphimedon*. These frequencies are also calculated across all those domains at once (column 1). Comparison of the conservation of binding residues versus non-binding residues; *, p<0.05; **, p<0.01; ***, p<0.001 (Probability associated with a Student's two-sample unequal variance t-Test).

Five separate homology models for the sponge PDZ3—CRIPT co-complex structure all demonstrated a shape that was highly similar to the rat crystal structure (average C_α_ RMSD 2.7 Å) (Figure 2A), with the largest deviations occurring in the N- and C-terminal regions and at the proline insertion in the sponge sequence between positions 318 and 319. To assess the effect of these rat-to-sponge sequence changes on binding, we computationally estimated the free energy of binding for the PDZ3—CRIPT interaction for the rat crystal structure and for each sponge model complex. The calculated total binding affinity varied by less than two-fold between these organisms, over all of the homology models, indicating that PDZ3 and CRIPT could have interacted in the animal ancestor.

Using three available crystal structures from *dlg* PDZ1-3 [14]–[17], a core union of 13 amino acids in contact with the ligand was defined (blue residues in Figure 2A). Within a specific PDZ domain these amino acids have 88.5% conservation among *Homo, Drososphila, Nematostella,* and *Amphimedon*; whereas the same conservation metric across the rest of the PDZ domain drops to ∼68% (Figure 2B). When the core union set of amino acids is compared between all PDZ domains its conservation is ∼59% suggesting that this group of amino acids—those that are most highly conserved within specific PDZ domains—are not only strong drivers of PDZ domain diversity and specificity but very likely gained their distinguishing features before the Eumetazoan-Poriferan ancestor.

### Conservation of Domain Organization

A defined arrangement of motifs among many post-synaptic proteins is a well-recognized architectural feature of the complex. For example, the supra-motif organization of *dlg* beginning at the amino terminus consists of an L27 domain, three adjacent PDZ domains, a Src homology-3 (SH3) domain, and an inactive guanylate kinase-like (GUK) domain (Figure S2.23.a). This organization is invariant and predates the origins of the animal kingdom as indicated by its presence in the choanoflagellate, *Monosiga brevicollis*, a class of protists considered to be the closest living relatives of the animals (data not shown).

Prokaryotes and fungi have very few PDZ domain genes. Based on analysis of recently available genomes, the choanoflagellate *Monosiga brevicollis* has 58 PDZ containing genes and the cnidarian, *Nematostella vectensis* has 66 PDZ containing genes. Of these, 15 can be considered orthologous in terms of bidirectional sequence alignments, but only seven are conserved through the entirety of their domain architectures (data not shown). *dlg* and *Shank* are among these seven. At least fifty-four PDZ containing genes, many of which are among the synaptic gene repertoire, were present in the Eumetazoan ancestor and are conserved till present day lineages of Deuterostomes, Protostomes and Cnidaria. Other gene families such as the cadherins and tyrosine kinases also appeared before the metazoa/choanozoa split and laid the ground work for rapid expansion of these families within metazoan lineages [18].

A conserved supra-motif organization implies evolutionary constraints on the manner in which the scaffold presents its ligands, and therefore, one might expect to find conserved features of inter-domain regions. Although the spacing between domains is not conserved (Figure S2.23.a), several highly conserved proline codons and other residues occur in the *dlg* inter-domain sequences. The positioning of ligands on the *dlg* platform does not appear to depend precisely on the spacing of the *dlg* PDZ domains; more likely axis rotation of the domains to allow multiple binding events as has been reported with PDZ1 and PDZ2 domains of syntenin [19] is the conserved feature.

Among the post-synaptic genes present in *Amphimedon,* many other interaction domains are also conserved making it likely that the ancient signature motifs were competent to interact with ligands. For example, in mammals the Shank—Cortactin interaction occurs through a PPVP motif in Shank and an SH3 domain in Cortactin, both of which are present in the *Amphimedon* genome. The interaction between Shank and Homer occurs via a conserved PPXXF motif in Shank, and an EVH1 domain in Homer which is also present in *Amphimedon* (as is an EVH1 domain in Cortactin). The conservation of PDZ ligand sequences is shown in Table 1. The mammalian Shank-mGluR interaction occurs via a PDZ interaction. *Amphimedon* Shank has a PDZ domain and its mGluR has a PDZ-binding motif. The Homer binding domain in IP3R is nearly identical throughout the Metazoa (the PPKKFR motif has a small change to PPMKFR in *Amphimedon*). On the other hand, GKAP, while present in *Amphimedon*, did not acquire its PDZ ligand sequence until after the protostome-deuterostome split. This observation suggests that the GKAP interaction with PSD-95 via its GK and SH3 domains [20] is more ancient than its association with Shank even though a Shank ortholog is present in the sponge.

### Expression of Post-synaptic Gene Orthologs in Sponge

**Figure 3:**
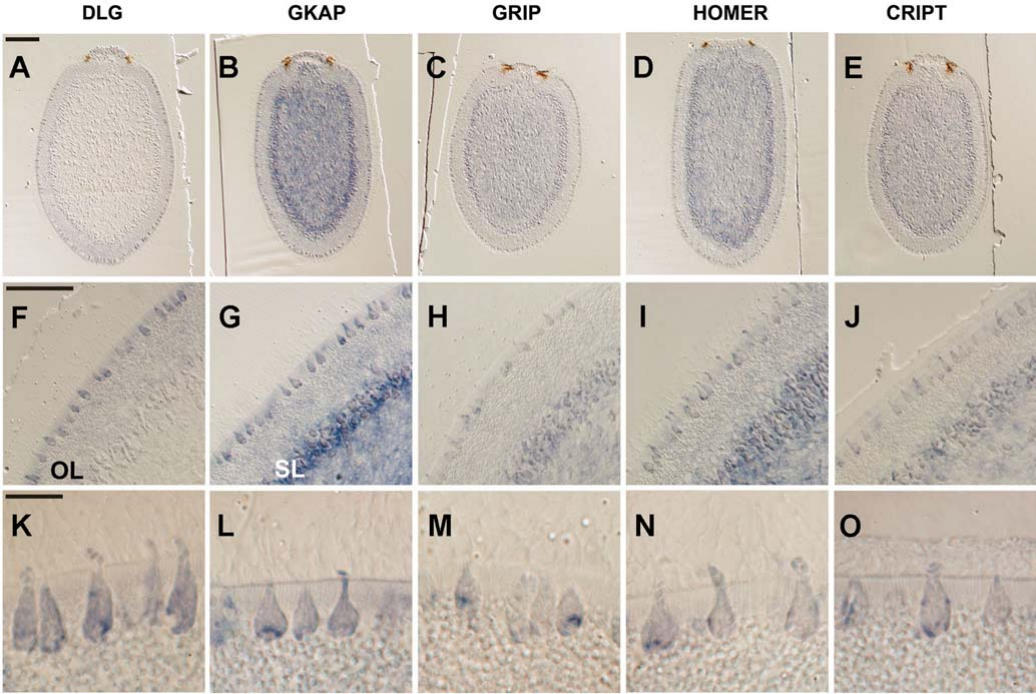
Expression of Post-Synaptic Orthologs in *Amphimedon* Larvae. DLG, GKAP, GRIP, HOMER and CRIPT. All five genes (listed across the top) are expressed in the flask cells of *Amphimedon* larvae. (A–E) Sections of whole mount *in situ* hybridized larvae, with the posterior pole to the top. OL, outer epithelial- like layer; SL, subepithelial (middle) layer; ICM, inner cell mass. (F–J) Magnification of OL and SLs, with flask cells distributed in OL. (K–O) Scale bars: a–e, 100 µm; f–j, 50 µm; k–o, 10 µm.

To determine whether the ‘synaptic’ genes identified in *Amphimedon* are expressed in the same cell type we performed *in situ* hybridization for DLG (*dlg*), HOMER, GRIP, CRIPT and GKAP using sponge gene probe sequences. All five genes were expressed in the flask cells, which are large ciliated cells that protrude from the columnar epithelial-like outer layer of the sponge larva (Figure 3). They are found through out the epidermis, but at higher density towards the anterior. Sections of an *Amphimedon* larva revealed that these genes are not expressed together in any other larval cell types. This expression data is therefore consistent with the existence of a proto-postsynaptic-like scaffold in larval flask cells.

In addition to the labelling in flask cells all five genes show pleiotropic expression patterns. *GRIP, HOMER* and *CRIPT* appear to be expressed in a subset of the flask cells (Figure S5). *dlg* transcripts also are detected throughout the outer epithelial- like layer (OL). Analysis of embryonic expression of *dlg* reveals that this gene is expressed early in cells fated for the outer layer and later down regulated in all cells, except flask cells (Figure S6). *GKAP* and *HOMER* also display relatively high levels of expression in the subepithelial (middle) layer (SL) and large cells inside the pigment ring and a subset of ICM cells. In addition to the flask cells, *GRIP* transcripts are detected at low levels in the SL and a few ICM cells. *CRIPT* is expressed in a similar pattern to *GRIP*. The differential expression patterns of these five genes support their specificity. Furthermore, our previous studies have demonstrated other unique labelling patterns in the larva [21].

## Discussion

The data presented here support the presence of a proto-post-synaptic scaffold in the last common ancestor to all living animals. The presence of a large number of post-synaptic genes in the genome of demosponge *Amphimedon*, the nearly absolute conservation of binding domains and ligands between this sponge and animals with neurons, as well as the expression of a set of post-synaptic mRNAs in the same cell type, suggest the proto-post-synaptic scaffold existed as an assembled functional structure very early in animal evolution. More definitive evidence will require immunocytochemical protein localization. The set of genes found in *Amphimedon* are mainly components of a sub-membranous specialization in animals with nervous systems known as the post-synaptic density (PSD). Although no clear morphological correlate of a PSD or a synapse has been observed in sponges, osmophilic regions with septae have been observed in the larvae of homoscleromorph sponges as well as impulse conduction in the syncytial hexactinellid sponge Rhabdocalyptus dawsoni ([22], [23]; and personal communication, Sally Leys).

The core potential for evolving synapses in sponges may extend to other types of junctions. As metazoan cell types emerged, both orthologs and paralogs of the genes found here became components of junctions other than synapses as well. Therefore, the set of protosynaptic genes (as well as other genes) might be viewed not as prototypical with regard to synaptic junction evolution, but as a set of genes capable of giving rise to a diversity of junctions. For example, the first and second PDZ domains of *dlg* diversified greatly in terms of its surrounding domain organization such that PDZ domains which possibly duplicated from *dlg* PDZ 1/2 are found in true metazoan genes such as Erbin/densin-180, scribbled, and ZO. These early metazoan paralogs may have contributed to the evolution of other junctions such as the tight junction and to the establishment of polarity in epithelial cells.

The *Amphimedon* cell type that most prominently expresses the post-synaptic orthologs is the flask cell found in larva and characterized by a deep cilium and a large population of basal vesicles. The flask cell may have some environmental sensing properties that require membrane specializations [24] and perhaps reflect an evolutionary intermediate and a cell type that served as a starting point for the evolution of neurons. Alternatively, the functional neuron evolved prior to the divergence of sponges and eumetazoan lineages, and selective loss of genes encoding transmembrane receptors yielded the flask cell, which in this scenario is an evolutionary relic of the nerve cell.

The modular structure of the proto-post-synaptic complex, defined in terms of its motif organization, changed little over the entire course of animal evolution. Yet, assuming sponges are sister to eumetazoans, the few innovations that came with the origination of nervous systems were novel binding partners to an existing scaffold (Table S3). These genes tend to be trans-membrane proteins. For example, both NMDA receptors and neuroligins seem to have appeared during evolution well after the *dlg* scaffold to which they bind. These two genes represent a central organizing unit for the excitatory glutamatergic synapse [25]. A Shaker type K channel, not present in *Amphimedon*, also linked to the pre-existing *dlg*. Similarly, the AMPA receptors appeared after GRIP, which serves as their scaffold. In fact, a GRIP PDZ 4 and PDZ 5 domain, the domains to which the AMPA receptors bind, is distinguishable in the sponge GRIP ortholog. And GKAP, albeit present in sponge, did not acquire its PDZ ligand sequence until after protostome-deuterostome split.

Whether orthologs of these few critical post-synaptic genes were lost in the sponge or acquired by an evolutionary descendant, does not negate the finding that a proto-post-synaptic complex existed at the origin of the animal kingdom. Sponge genomes can reveal shared features that predate nervous systems and cell-specific adaptations, such as the synaptic junction. The evolution of the nervous system required network strategies by which novel binding partners can compete with pre-existing ligands in a manner that does not disrupt the assembly of the post-synaptic complex. Up-regulation of scaffold protein expression represents a possible solution to this issue, and indeed most of the scaffold proteins represent abundant components of the synapse.

Ancestral membrane-spanning receptors related to those genes which appeared to transform the proto-synaptic scaffold to a functional synaptic machine were present in neoproterozoic animals, possibly lost in the sponge, and may have critically contributed to the evolution of the post-synaptic complex in a Poriferan-Eumetazoan common ancestor. Relatively minimal modifications of the core modular structure could result in functional evolutionary leaps. The existence of a versatile proto-synaptic complex suggests that the post-synaptic junction has a modular past which is seamlessly inapparent except through its evolutionary history.

## Materials and Methods

### Isolation of *Amphimedon* genes

Genomic traces and ESTs were generated as part a collaborative genome project with the Joint Genome Institute and are publicly available (http://www.ncbi.nlm.nih.gov/Traces). Genes were identified in EST and genome trace archives based on similarity to vertebrate sequences. The 5′ part of these genes was cloned by means of 5′ RACE using BD Smart Kit (ClonTech) and gene specific primers. The complete coding sequence was confirmed by RT-PCR of embryonic RNA (primer sequences available upon request).

### Data Collection and Phylogenetic Analyses

For selected synaptic genes, protein sequences of *Homo sapiens, Drosophila melanogaster, Saccharomyces cerevisiae, Dictyostelium discoideum, Arabidopsis thaliana*, and *Oryza sativa* orthologs were collected by BLASTP searches on the NCBI blast server (http://www.ncbi.nlm.nih.gov/BLAST/) following a symmetrical best hit approach (SymBet) [26] with a cut-off threshold of E-value e^−5^ or less. For each lineage, the dataset was enlarged, when possible, by the inclusion of co-orthologs [27] which are two or more genes in one lineage that are, collectively, orthologous to one or more genes in another lineage due to lineage specific duplications. As an example, human SAP-97, PSD-95, SAP-102 and Chapsyn-110 are co-orthologs with fly *dlg*.

Those sequences were used to query the database of *Amphimedon queenslandica* genomic traces and *Nematostella vectensis* genomic contigs [28] by tBlastn [29] for SymBets. For *Amphimedon* queries, the matching traces were collected, manually evaluated and assembled into genomic contigs using an in house assembly pipeline (sequential use of MegaBlast [30] for selection of additional traces and PHRAP or PCAP assemblers [31], [32] for construction of the contigs). To reduce the risk of analyzing non-sponge contaminants, any genomic region was required to be present on multiple traces. Gene intron/exon structures were identified using GenomeScan [33] and GENSCAN [34] and additional manual inspection. Conserved regions were subjected to further scrutiny to make sure the homology spans a considerable length of the original gene. *Nematostella* sequences were also predicted by GenomeScan and GENSCAN and added to the dataset in cases where an *Amphimedon* ortholog could not be found.

Although it is not easy to resolve the early metazoan phylogenetic history [35], we focused on the simpler task of whether a given gene family originated before or after the Poriferan-Eumetazoan (or Cnidarian-Bilaterian) split. For each *Amphimedon* or *Nematostella* candidate for orthology, we ran comprehensive phylogenetic analyses. If orthologs of the gene family are found in selected non-animal species, then the choice of outgroups for the phylogenetic tree is straight-forward; they are the gene family members from those non-animals. On the other hand, if the gene family members are found only in animals, phylogeny is best established by including other closely related gene families in the dataset. Most closely related gene families are often outparalogs [26]; paralogous genes resulting from a lineage-specific duplication preceding a given speciation event. Accordingly, our dataset was enlarged, when possible, by the inclusion of the most closely related outparalogs from the above mentioned genomes with respect to the Poriferan-Eumetazoan or Cnidarian-Bilaterian splits. Inclusion of outparalogs in the dataset ensured that we were not incorrectly classifying a closely-related gene in a wrong family. In some cases, it was not possible to enlarge the dataset due to lack of any outparalogs, so we had no choice but to make phylogeny with *Amphimedon* or *Nematostella* sequences as the root of the tree. In those cases, orthology was further concluded by the requirement of a high-scoring SymBet and shared domain architecture. Protein accession numbers for all those proteins in each dataset are provided in Table S1.

Protein domains were identified using PFAM and SMART [36], [37] and those domain architectures for each protein family from selected species are illustrated in Figure S2. Sequences spanning one or more of those shared domains were used for multiple sequence alignment with ClustalW [38]. For uncertain cases, alignments with T-Coffee [39] and different ClustalW parameters were compared to combine them into a final alignment. Gene family phylogenies were determined using Maximum Parsimony (MP) and Neighbour Joining (NJ) of p-distances, implemented in PAUP* [40]; Maximum Likelihood (ML) implemented in phyml [41]; and Bayesian MCMC (BMCMC), implemented in MrBayes [42]. To assess confidence in individual nodes, MP, ML and NJ bootstrapping were implemented with 1000 pseudoreplicates and BMCMC posterior probabilities were reported. For ML analyses, the best-fit likelihood model was determined using ProtTest [43]. The BMCMC analyses were conducted by running two independent analyses, each with four heated Markov chains, using a mixed amino-acid model, until a convergence diagnostic (standard deviation of split frequencies (SDSF)) fell below 0.01. Initial steps in the Markov chains were discarded as burn-in. Trees were rooted by selecting an outgroup gene according to the most parsimonious duplication and loss scenario using NOTUNG 2.0 [44] with respect to conventional species trees. Those phylogenetic trees are illustrated in Figure S1.

As a special case, phylogeny of 12 PDZ domain-containing protein families were determined with a single domain tree which includes selected PDZ domains of those proteins, numbered according to their spatial distribution on their corresponding scaffold. Those sequences were aligned using the global pair algorithm of MAFFT [45] which aligns large sets of distantly related sequences of similar length with high accuracy. For phylogenetic analysis, only a single human ortholog for each of those PDZ domain-containing proteins were included to ensure the BMCMC trees converge (otherwise our computers were not able to obtain a SDSF below 0.01).

### Computation of Structures

Hydrogen atoms were added to the 1BE9 rat crystal structure using the HBUILD facility [46] and the PARAM22 parameter set [47] of CHARMM [48]. All water molecules were removed from the structure, except for those making hydrogen bonds with the peptide. Missing side chains (F301, D332, and K5) were re-built, and the missing atoms of K5 were then minimized.

Homology models were generating using Modeller version 8v2 [49], using the rat (1BE9) crystal structure and each other sequence as aligned in Figure S3. Ten models were requested for each sequence, and the structure was selected that had the lowest Modeller objective function without errors in PROCHECK [50]. The five homology models referenced for sponge are the first five homology models generated by Modeller. Binding free energies were calculated as the sum of van der Waals, solvent-accessible surface area [51], and continuum electrostatic interaction and solvation terms, after minimizing the CRIPT peptide in the bound-state.

### In Situ Hybridization

Whole mount in situ hybridizations were performed as described [21]. In short, larvae were fixed in 4% paraformaldehyde/0.05% glutaraldehyde in MOPS buffer and stepped into 70% ethanol. After rehydratation, proteinase K treatment and postfixation, larvae were hybridized over 20 h with digoxigenin-labeled antisense riboprobes transcribed from cDNAs fragments cloned into pGEMT plasmid. Probe lengths were as follows: DLG: 1kb, Homer: ∼ 800 bp, GKAP: ∼500 bp, GRIP: 400 bp, CRIPT: 350 bp. Following washes to remove excess probe larvae were incubated with alkaline phosphatase-conjugated anti-digoxigenin antibody, washed, and the color reaction was performed using NBT/BCIP as a substrate. Larvae were photographed whole mount and subsequently dehydrated in ethanol and infiltrated with Epon 812. Sections were cut at 5 um and mounted in Histomount. A detailed protocol is available upon request.

## Supporting Information

Figure S1 Phylogenetic analyses of post-synaptic gene families. Statistical values of each essential clade is given in the order of, top left box, Bayesian Inference; top right box, Maximum likelihood; bottom left box, Maximum parsimony; bottom right box, Neighbor joining. Red and yellow colored clades represent gene families that originated before Poriferan-Eumetazoan and Cnidarian-Bilaterian splits, respectively. Green tagged sequences are from *Amphimedon queenslandica* and blue tagged sequences are from *Nematostella vectensis*. Abbreviations used in trees are: Sponge, *Amphimedon queenslandica*; CN, *Nematostella vectensis*; Human, *Homo sapiens*; Fly, *Drosophila melanogaster*; Yeast, *Saccharomyces cerevisiae*; Dicty, *Dictyostelium discoideum*; At, *Arabidopsis thaliana*; Os, *Oryza sativa*.
(2.97 MB PDF)

Figure S2 Domain architectures of post-synaptic gene families. Domain architectures of representative members for each gene family are displayed as SMART output. Output was manually edited for legibility for some PFAM domains, otherwise it is presented as SMART prediction. Abbreviations used in displays are: Sponge, *Amphimedon queenslandica*; CN, *Nematostella vectensis*; Human, *Homo sapiens*; Fly, *Drosophila melanogaster*; Beetle, *Tribolium castaneum*.
(3.66 MB PDF)

Figure S3 DLG PDZ-3 sequence alignment through 8 species. Alignment of PDZ3 sequences used for homology modeling. Conserved residues are colored according to the scheme shown. Residues that can interact with CRIPT are noted by a gray line.
(0.10 MB PDF)

Figure S4 Structural interactions between the C-terminus of the CRIPT peptide and the PDZ3 domain. (A), the C-terminus of the CRIPT peptide and the PDZ3 domain in rat, fish, fly, mosquito, cnidarian, sea urchin, and worm and (B), the C-terminus of the CRIPT peptide and the PDZ-3 domain in sponge. All the distances shown in part A and the “ray” marks indicating hydrophobic contacts are based on the rat (1BE9) crystal structure. Distances to Lys5 are shown to the beta-carbon because this is the last atom whose coordinates were reported in the crystal structure. Hydrophobic contacts from residues G322, A376, and L379 were found to interact in some organisms, but in the rat they were somewhat distant. For each residue, the main label corresponds to the rat sequence, and the smaller (or “residue name”) notes correspond to other organisms (not rat or sponge). Two interactions seen in the worm homology model were not included because they were unlike those seen in other organisms (G330 and P335). In the sponge diagram (part B), interactions that are possible given homology to the rat sequence are indicated, such as the hydrogen-bonds between PDZ3 and peptide. Residue numbering in the sponge structure corresponds to the aligned residue in the rat sequence. Some of the homology models suggested variant hydrogen-bonding patterns in the sponge, especially for residues Gln6 and Asn326. The figure was created using Ligplot and HBPLUS. The identical residues between rat and sponge are: 311, 312, 314, 318, 322*, 323*, 324*, 325*, 326*, 327*, 328*, 329, 330, 331, 335, 336, 337, 338, 339*, 341, 345, 347, 351, 353, 354, 356, 357, 359, 360, 362, 363, 364, 367, 371, 372*, 373, 375, 376, 378, 379, 380*, 382, 383, 385, 386, 387, 392, 393, 394, 396, and 400. Residues that make direct contact with the CRIPT ligand in the rat crystal structure are marked with an asterisk.
(0.13 MB PDF)

Figure S5 A surface view of an *Amphimedon* larva showing HOMER is expressed in a limited number of flask cells (arrows) by whole mount *in situ* hybridization. The large cells are the flask cells interspersed among the more numerous columnar epithelial cells. Transcripts are not detected in the remaining negatively stained flask cells in this field of view.
(0.07 MB PDF)

Figure S6 Developmental expression of *dlg* during *Amphimedon* embryogenesis. All panels are sections of *in situ* hybridized embryos; posterior pole is to the top. In the blastula, a small number of small cells express *dlg*. During the gastrulation-like stage, *dlg*-expressing cells sort to the outer layer; no expression is detected in the inner cell mass. At the later spot and ring stages, prior to hatching, cells expressing *dlg* are restricted to the outer epithelial-like layer. After hatching (Figure 3), flask cells in this layer express *dlg* at a higher level than the surrounding columnar epithelium.
(1.01 MB PDF)

Table S1 Protein accession numbers for sequences used in phylogenetic analyses. Each table contains the accession numbers for the sequences used in the corresponding tree from Figure S1. PDZ and iGluR genes/domains that are shown in table S1.19 and S1.23 in grey letters are not included in phylogenetic analyses. In table S1.23, PDZ domain amino-acid locations on their corresponding proteins are shown in parenthesis after abbreviation and all gi numbers correspond to their proteins. Abbreviations used are: Sponge, *Amphimedon queenslandica*; CN, *Nematostella vectensis*; Human, *Homo sapiens*; Fly, *Drosophila melanogaster*; Yeast, *Saccharomyces cerevisiae*; Dicty, *Dictyostelium discoideum*; At, *Arabidopsis thaliana*; Os, *Oryza sativa*.
(0.03 MB PDF)

Table S2 Sponge *dlg* and Human SAP97 intron/exon structure comparison. Cloned cDNAs of Sponge (*Amphimedon*) *dlg* were fully sequenced and mapped to its genomic sequence. Intron/exon structure was compared with its human ortholog SAP97, and about half the exons were found to be almost same size (highlighted in red) and content (in bold). However, human SAP-97 introns were in average 100 times larger than *Amphimedon dlg* introns (data not shown).
(0.03 MB PDF)

Table S3 Presence of post-synaptic gene orthologs in animals, yeast, dicty and plants. Species abbreviations used: Human, *Homo sapiens*; Fly, *Drosophila Melanogaster*; Nema, *Nematostella vectensis*; Sponge, *Amphimedon queenslandica*; Yeast, *Saccharomyces cerevisiae*; Dicty, *Dictyostelium discoideum*; Plants, *Arabidopsis thaliana* and *Oryza sativa*.
(0.08 MB PDF)
