# Supplementary material for: A Post-Synaptic Scaffold at the Origin of the Animal Kingdom
Source: PLoS One. 2007 Jun 6;2(6):e506. doi: 10.1371/journal.pone.0000506 (PMC1876816; doi:10.1371/journal.pone.0000506)
Supplement: Figure S1 — Phylogenetic analyses of post-synaptic gene families. Statistical values of each essential clade is given in the order of, top left box, Bayesian Inference; top right box, Maximum likelihood; bottom left box, Maximum parsimony; bottom right box, Neighbor joining. Red and yellow colored clades represent gene families that originated before Poriferan-Eumetazoan and Cnidarian-Bilaterian splits, respectively. Green tagged sequences are from Amphimedon queenslandica and blue tagged sequences are from Nematostella vectensis. Abbreviations used in trees are: Sponge, Amphimedon queenslandica; CN, Nematostella vectensis; Human, Homo sapiens; Fly, Drosophila melanogaster; Yeast, Saccharomyces cerevisiae; Dicty, Dictyostelium discoideum; At, Arabidopsis thaliana; Os, Oryza sativa. (2.97 MB PDF) [file pone.0000506.s001.pdf]

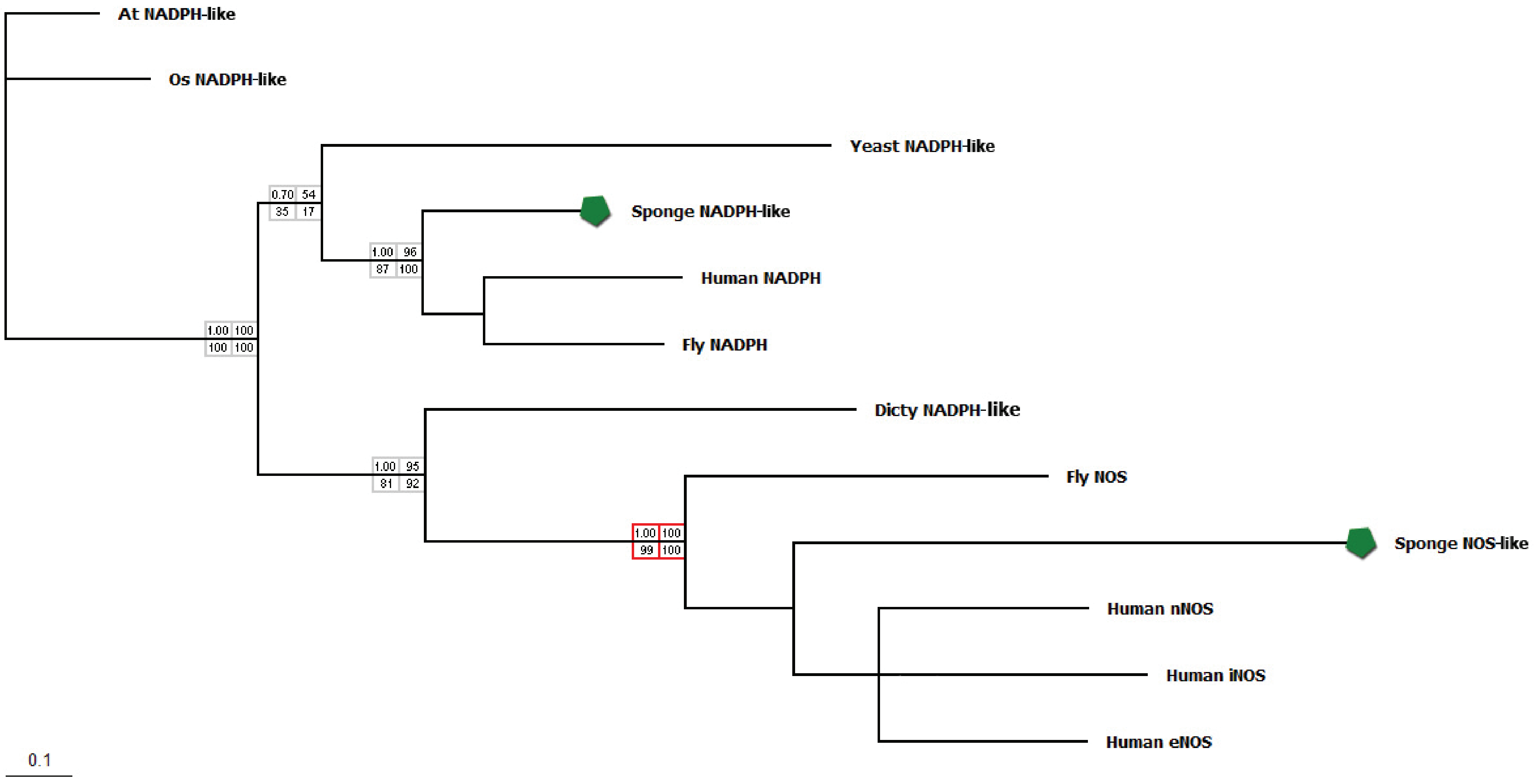

Figure S1.1. Phylogenetic analyses of NOS family.

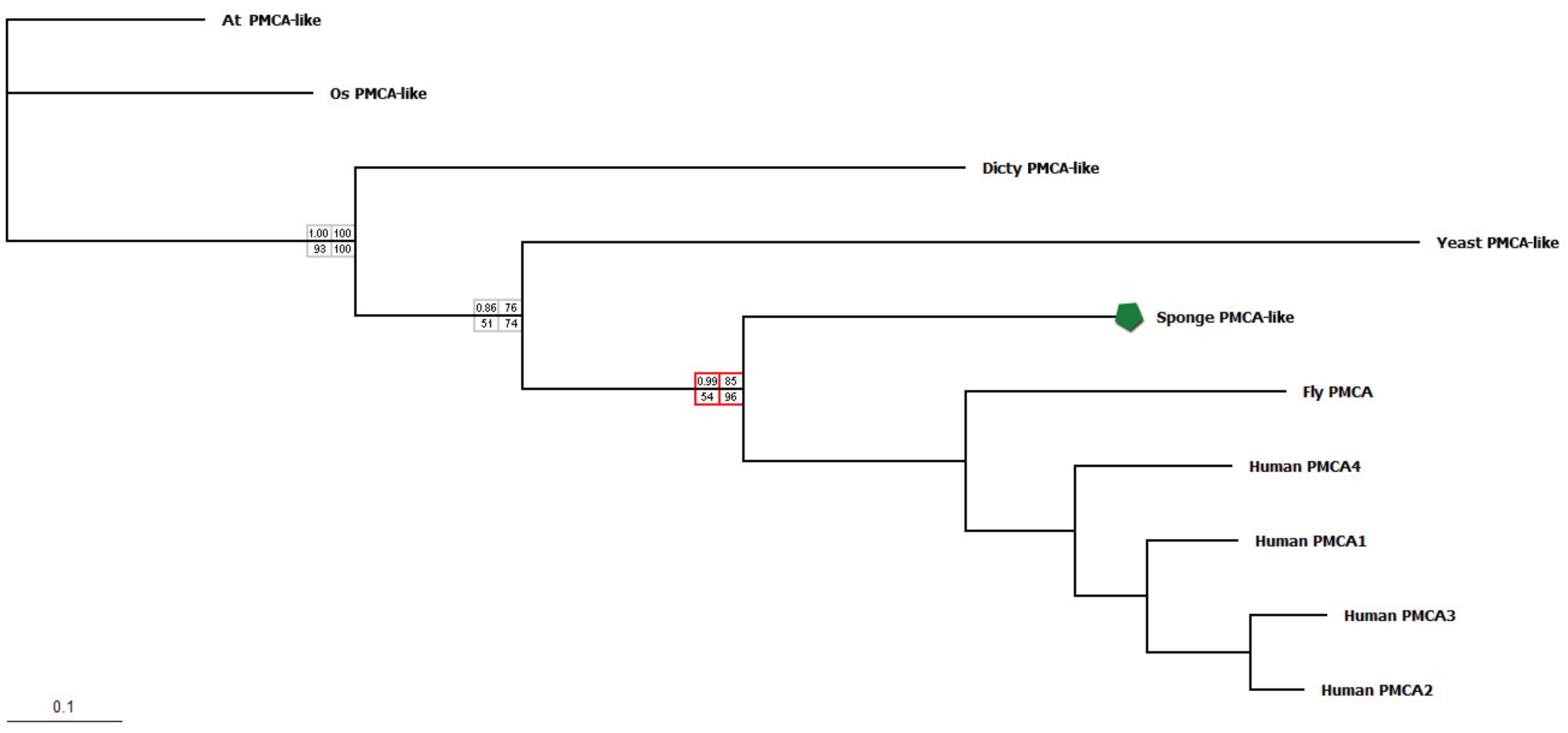

Figure S1.2. Phylogenetic analyses of PMCA family.

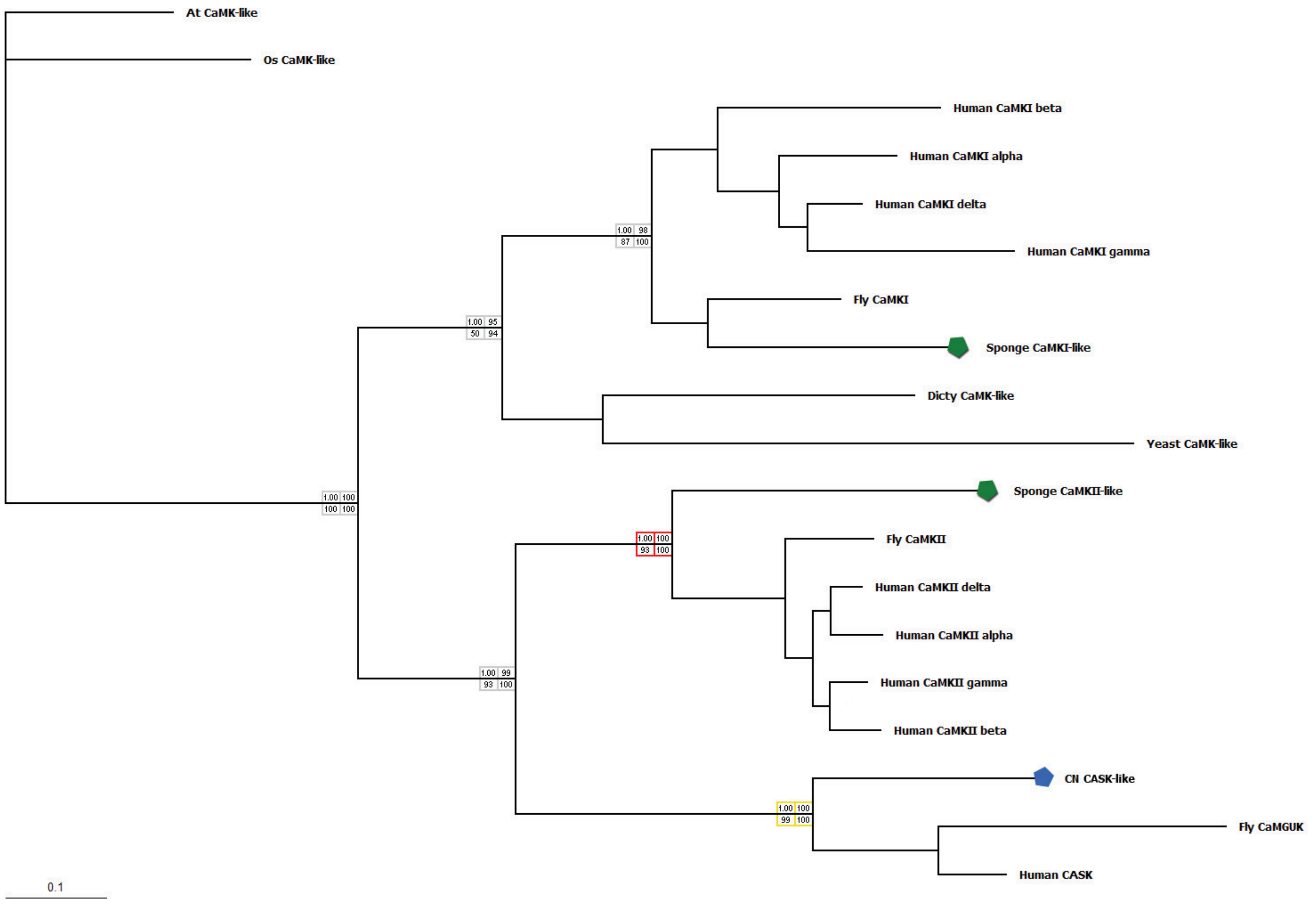

Figure S1.3. Phylogenetic analyses of CaMKII and CASK families.

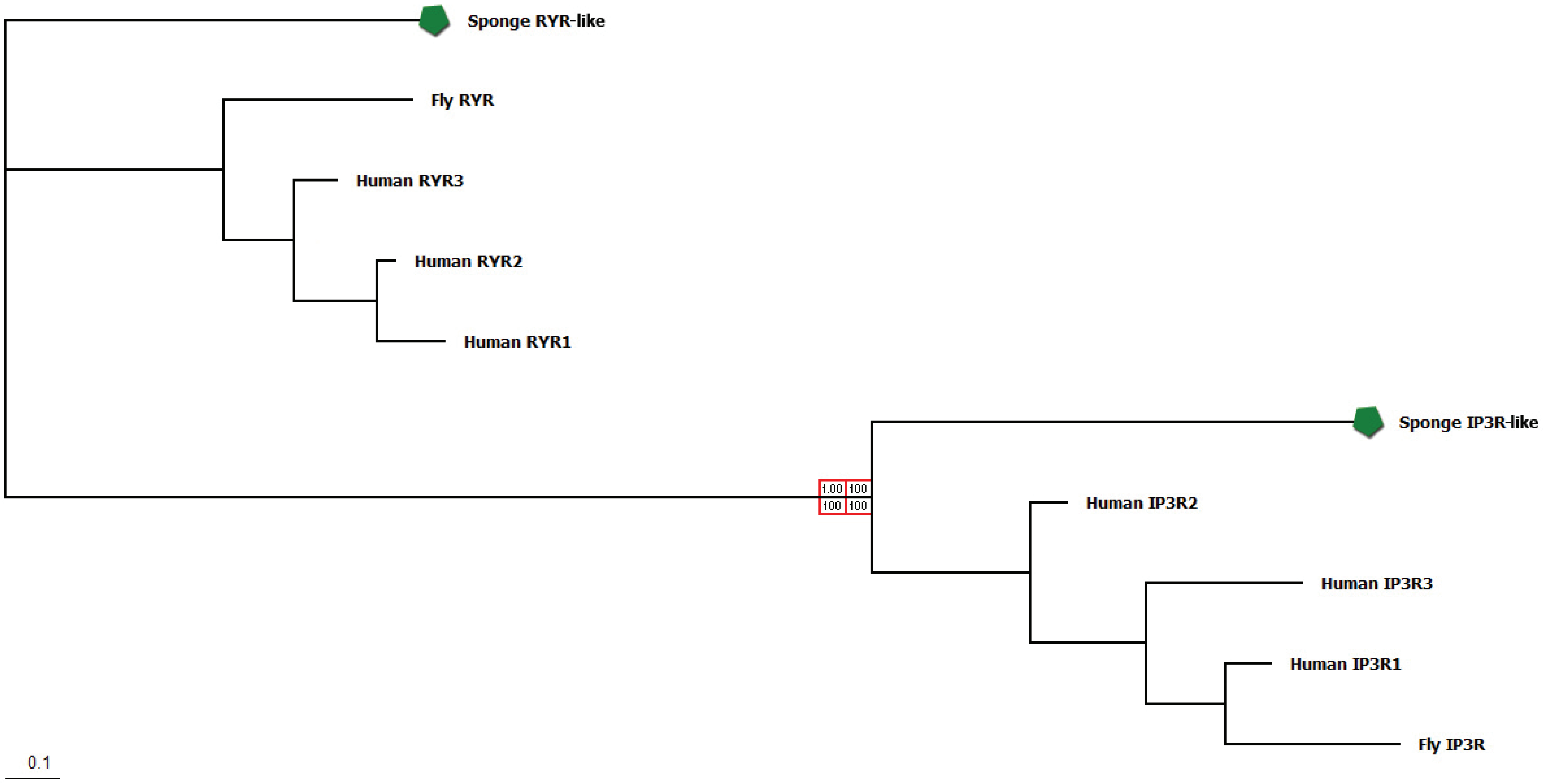

Figure S1.4. Phylogenetic analyses of IP3R family.

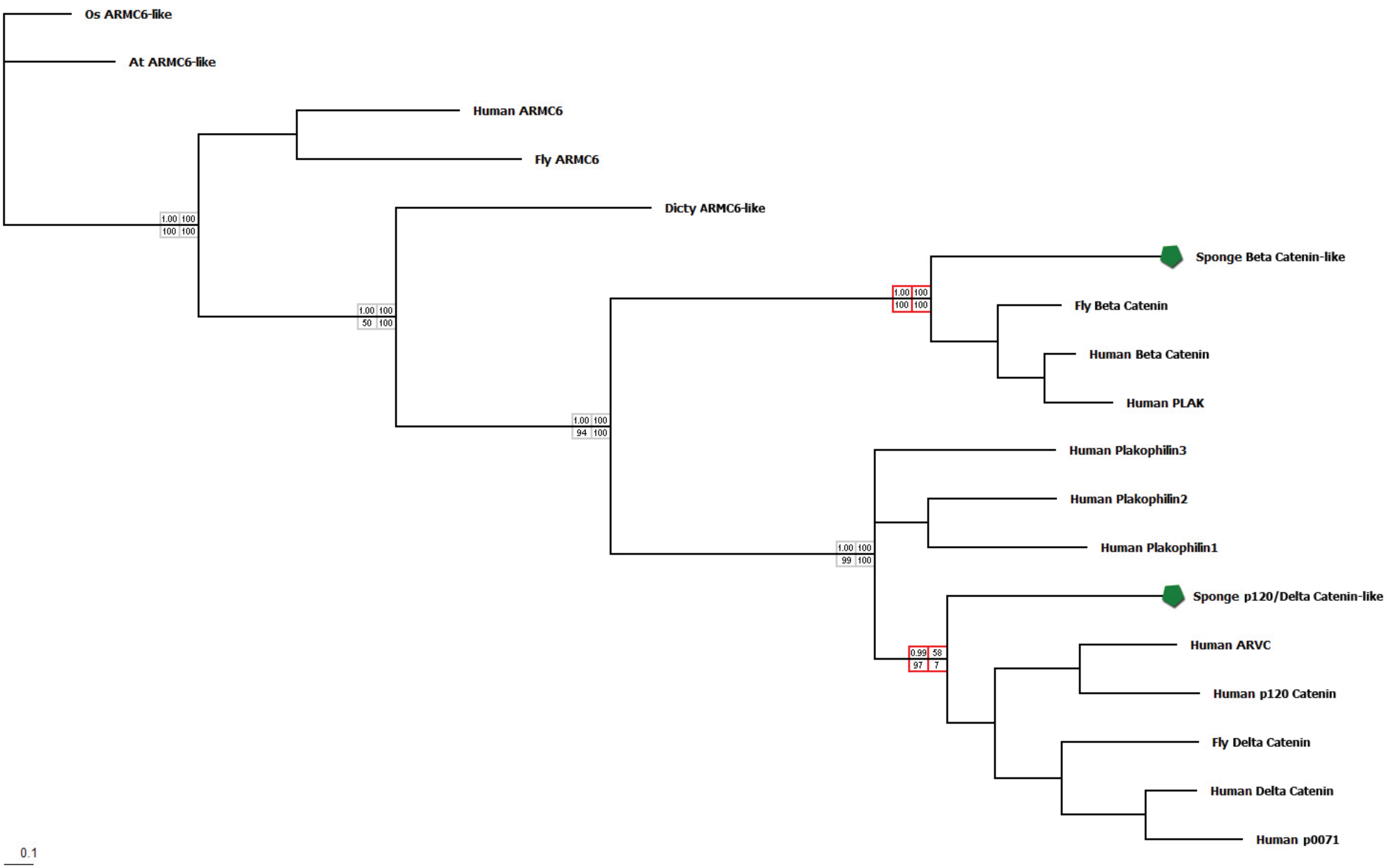

Figure S1.5. Phylogenetic analyses of Delta and Beta Catenin families.

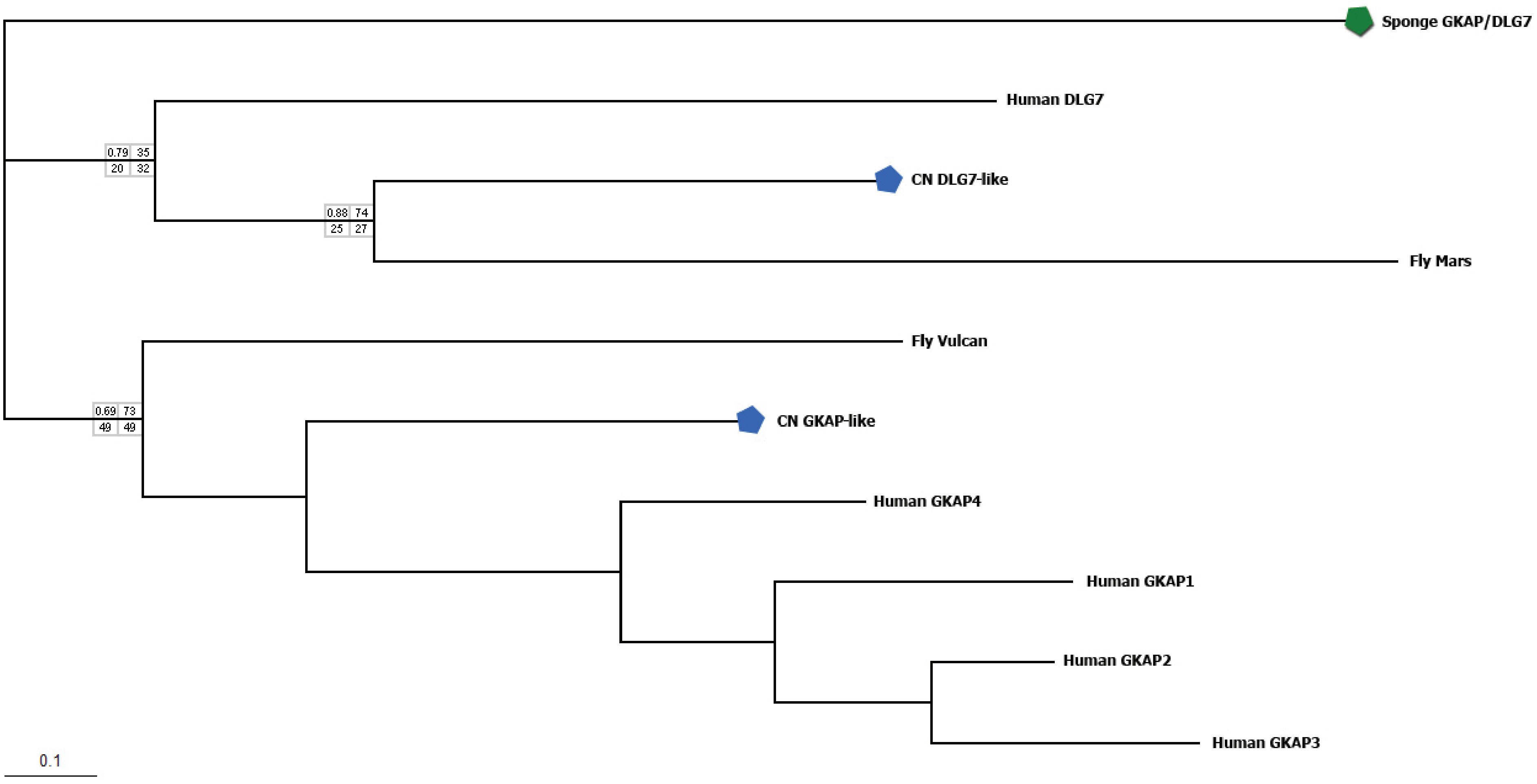

Figure S1.6. Phylogenetic analyses of GKAP family.

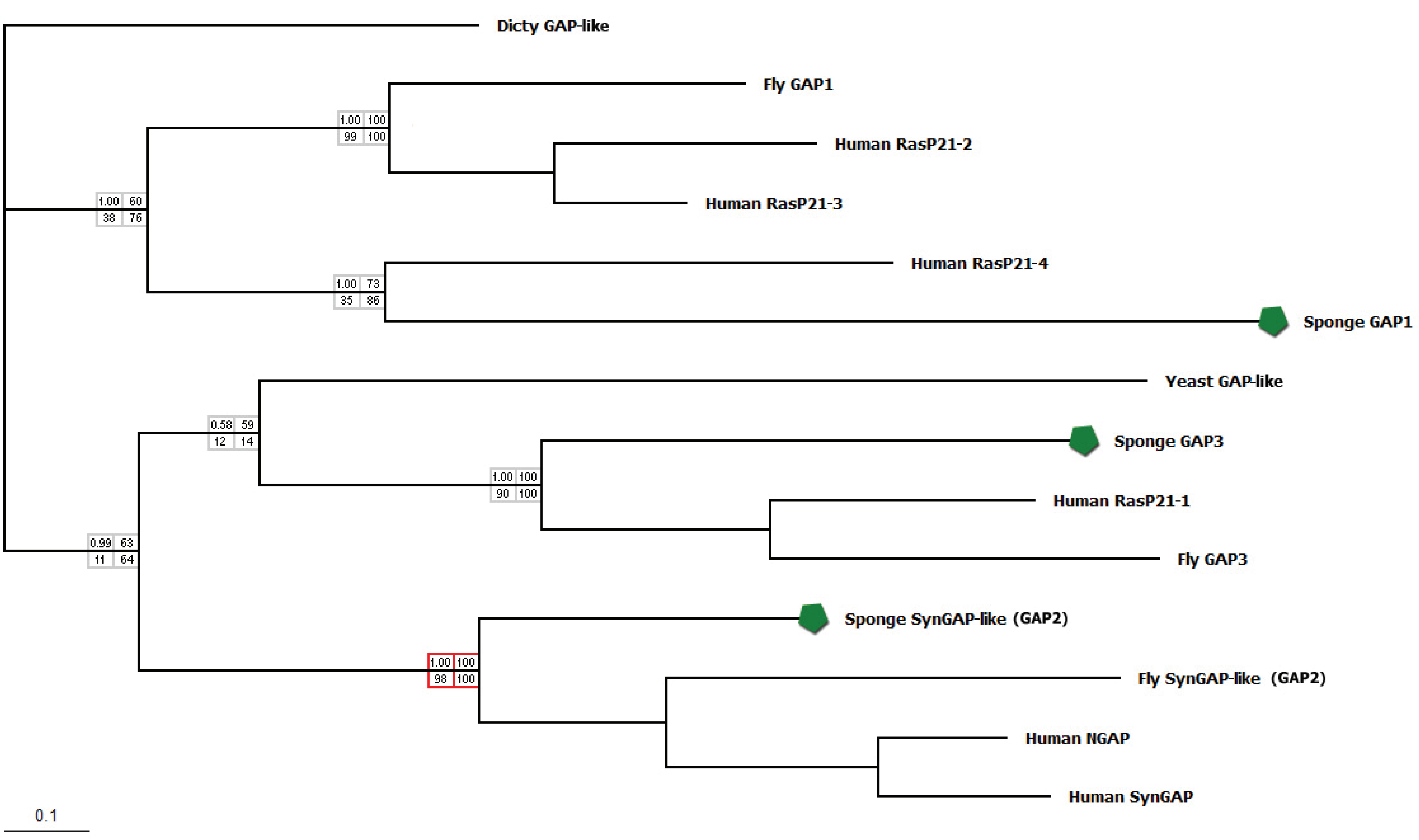

Figure S1.7. Phylogenetic analyses of SynGAP family.

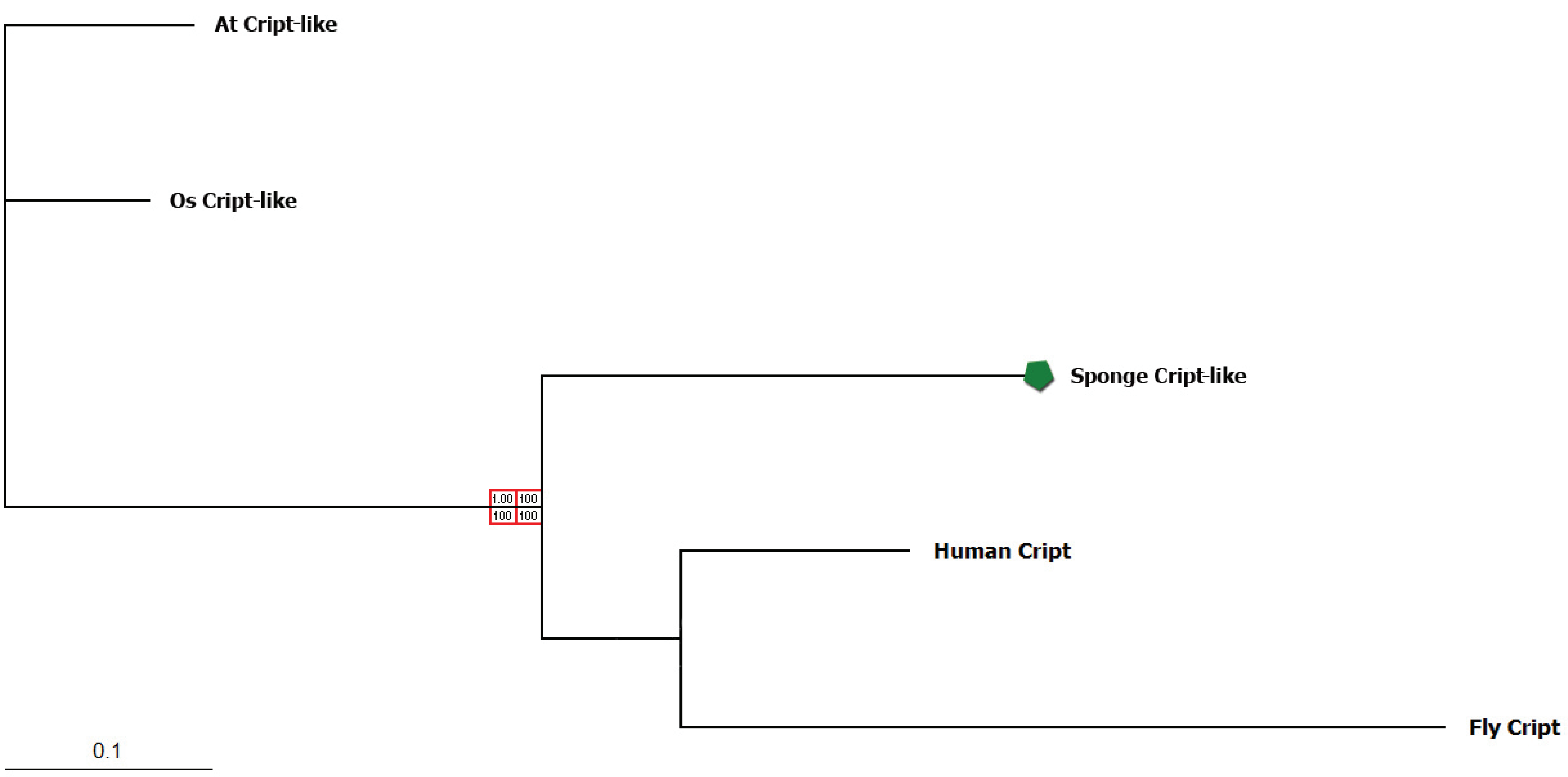

Figure S1.8. Phylogenetic analyses of CRIPT family.

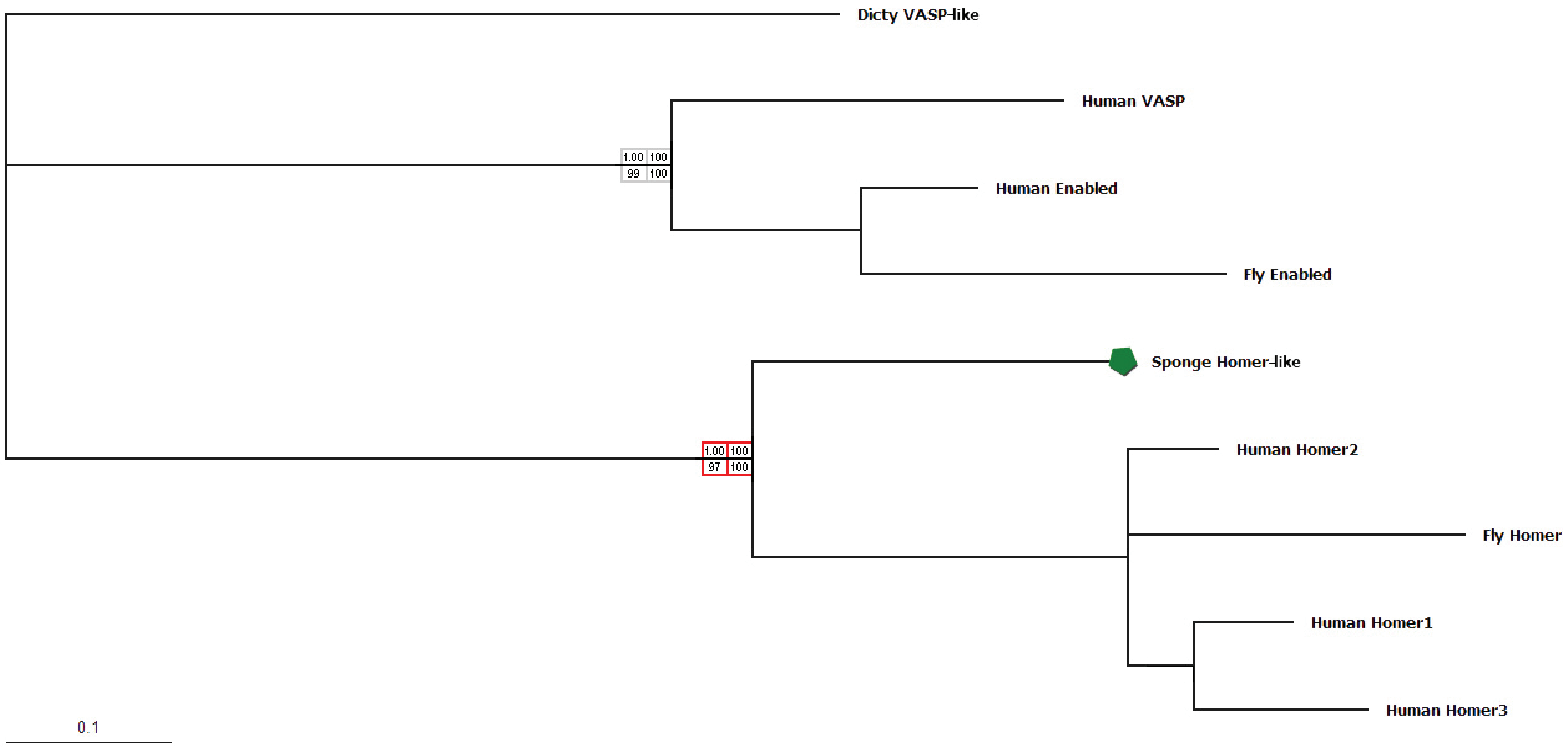

Figure S1.9. Phylogenetic analyses of Homer family.

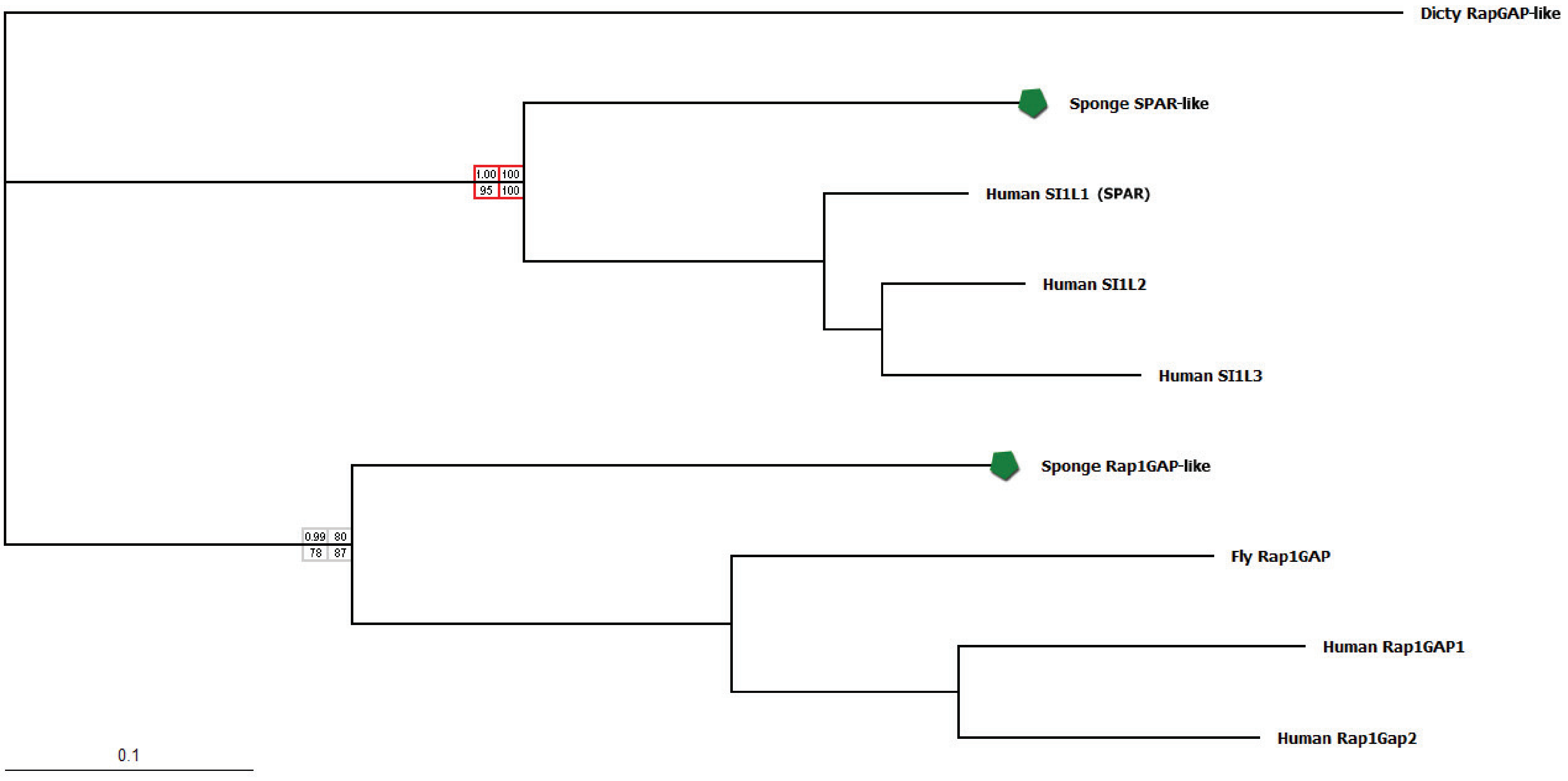

Figure S1.10. Phylogenetic analyses of SPAR family.

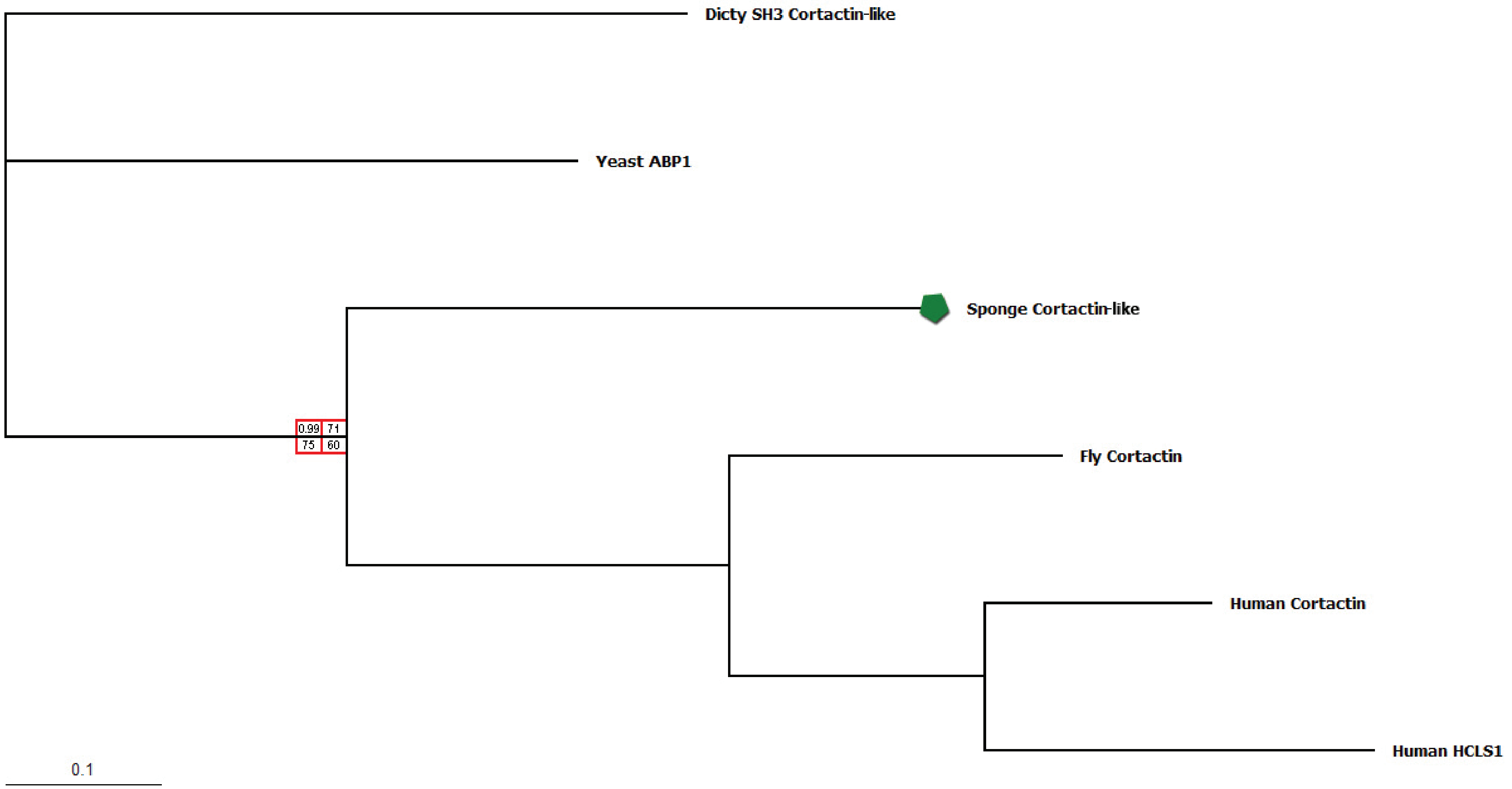

Figure S1.11. Phylogenetic analyses of Cortactin family.

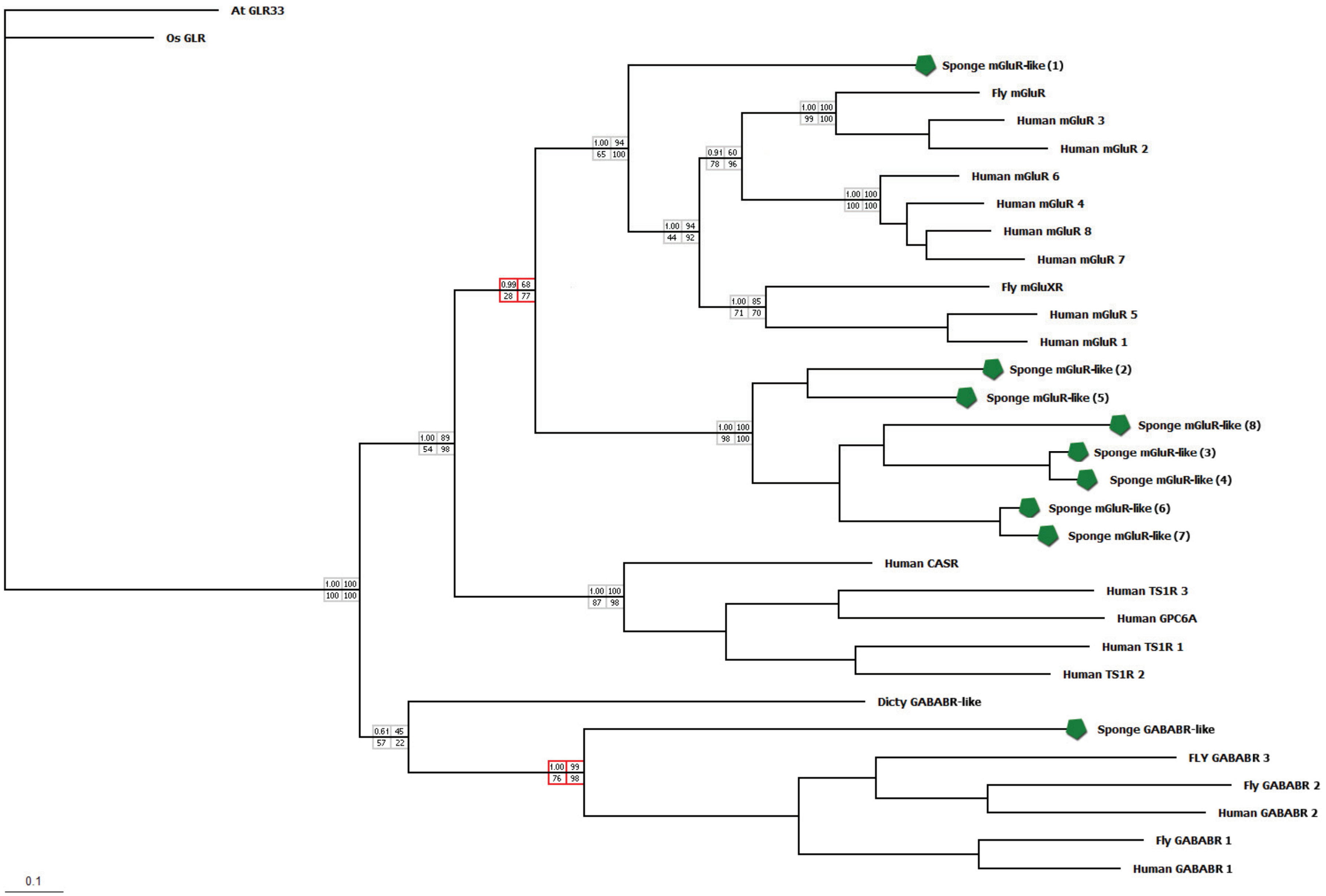

Figure S1.12. Phylogenetic analyses of Metabotropic GluR and GABAB Receptor families.

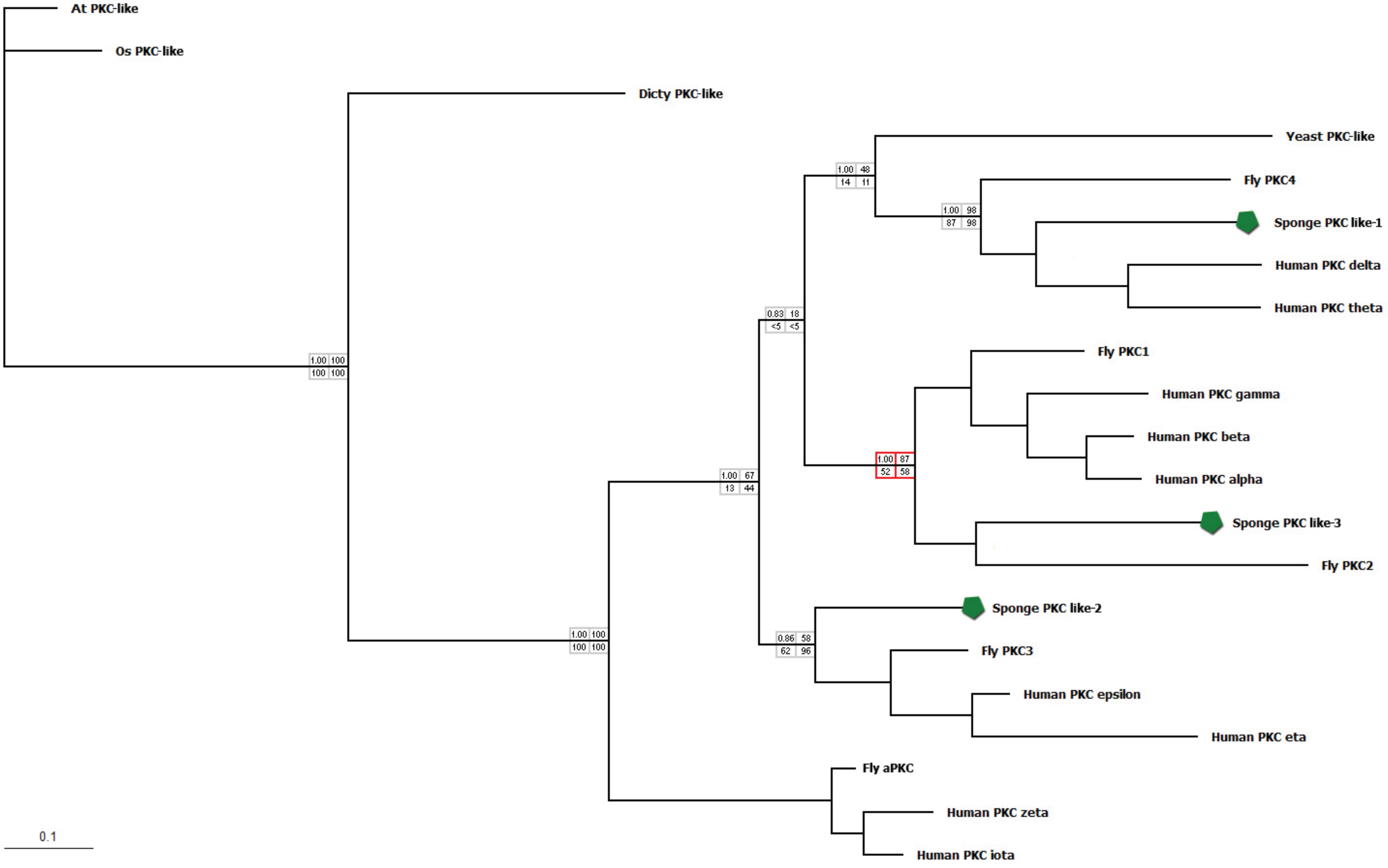

Figure S1.13. Phylogenetic analyses of PKC family.

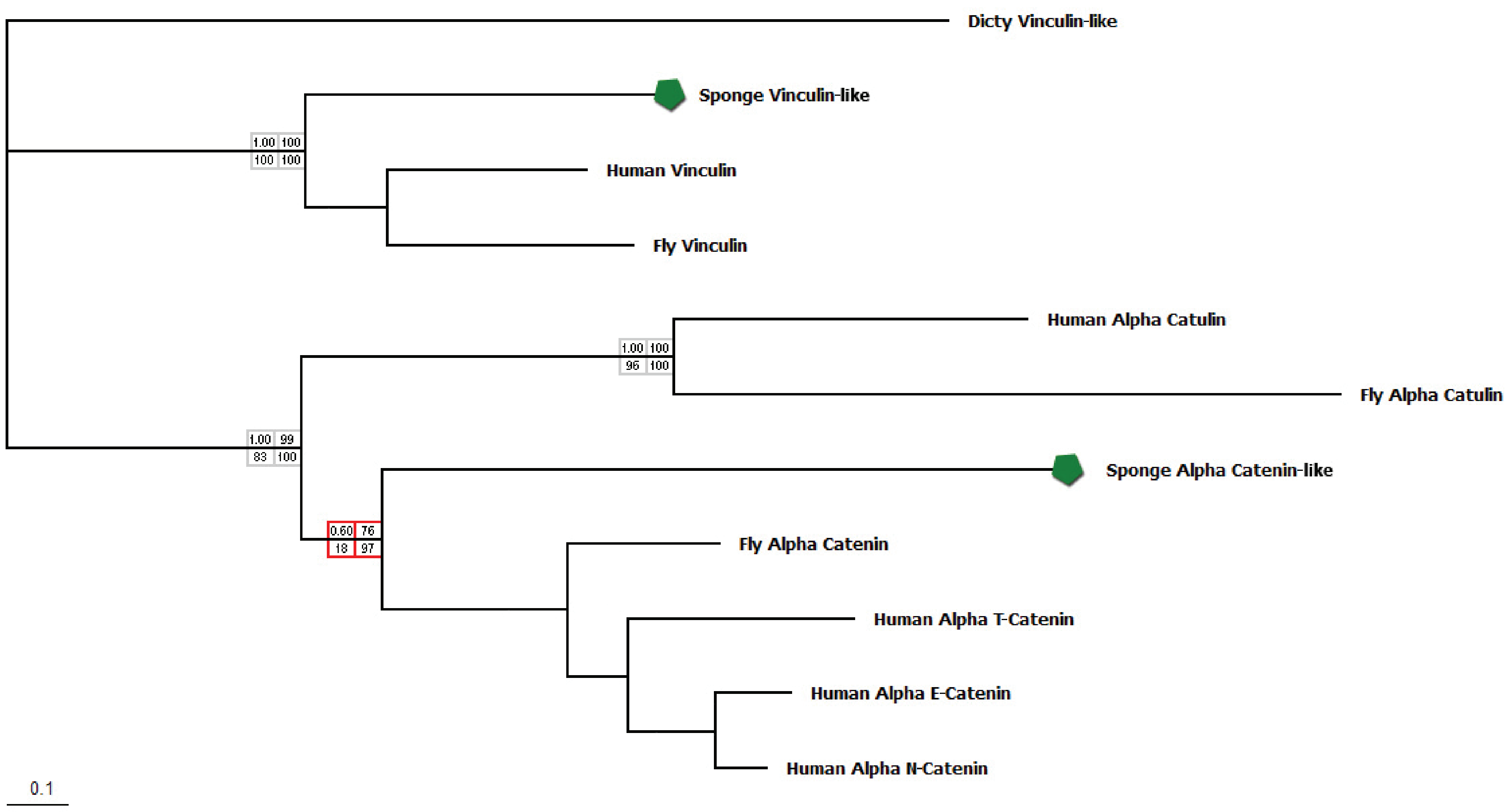

Figure S1.14. Phylogenetic analyses of Alpha Catenin family.

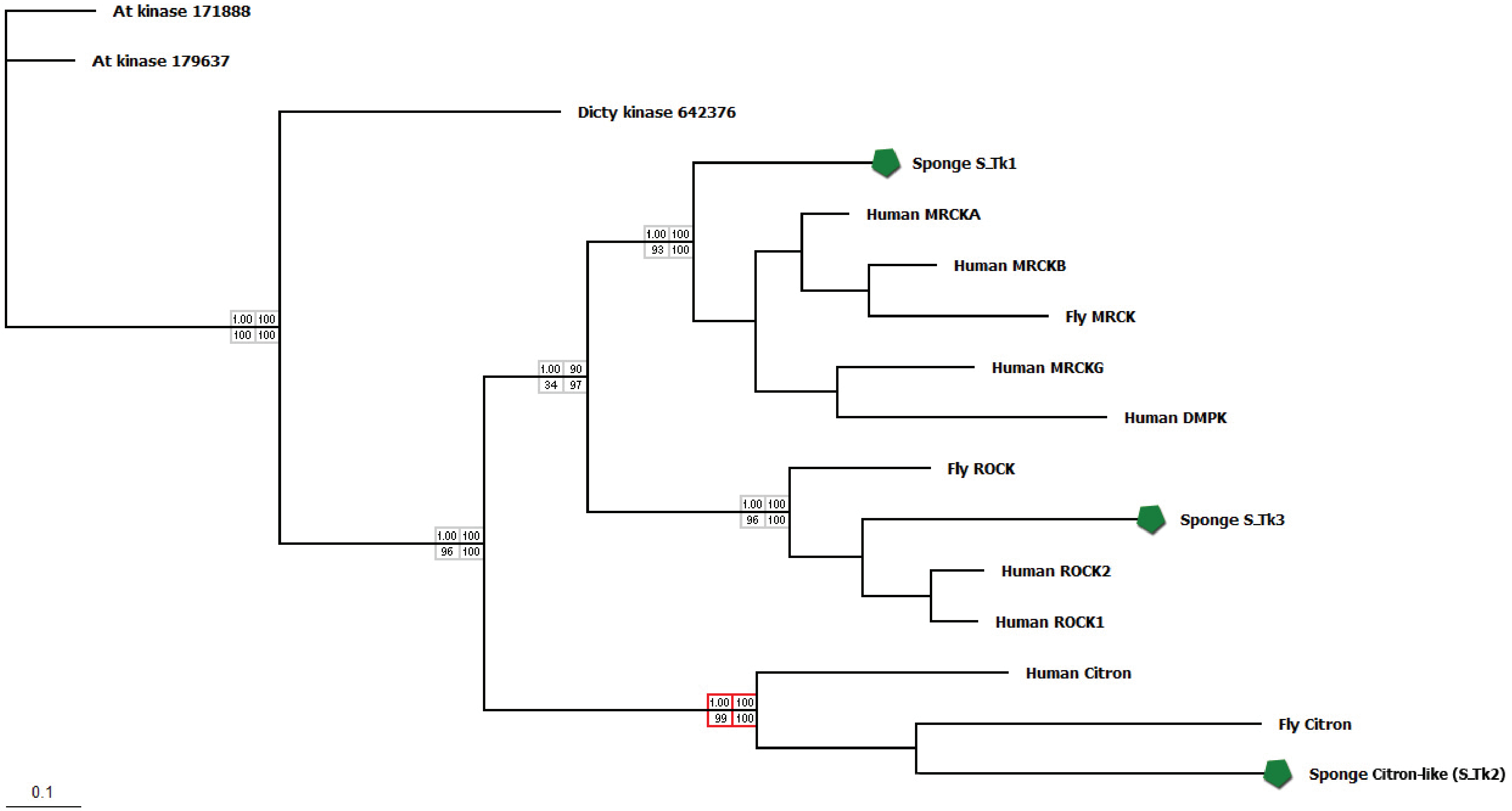

Figure S1.15. Phylogenetic analyses of Citron family.

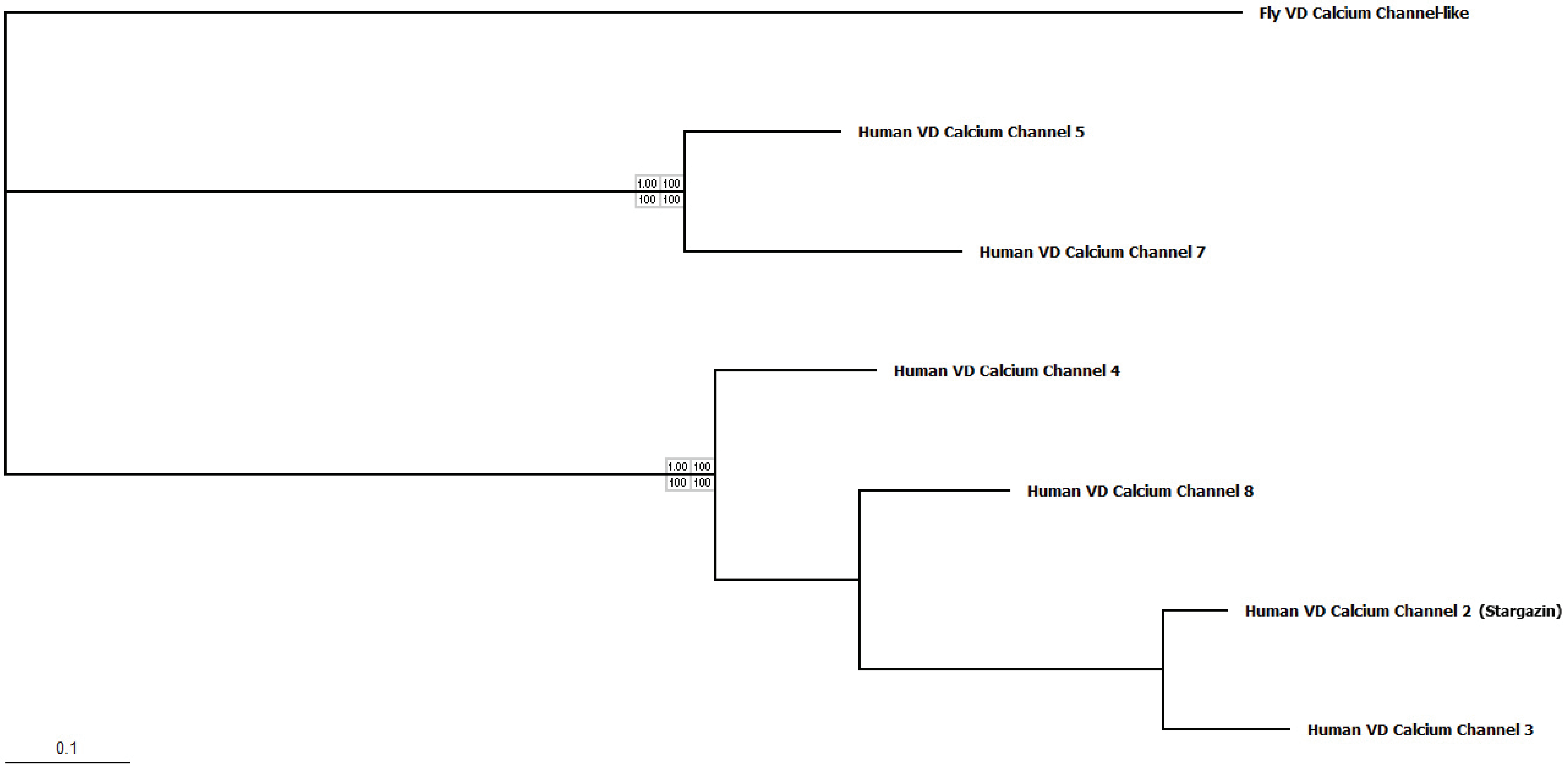

Figure S1.16. Phylogenetic analyses of Stargazin family.

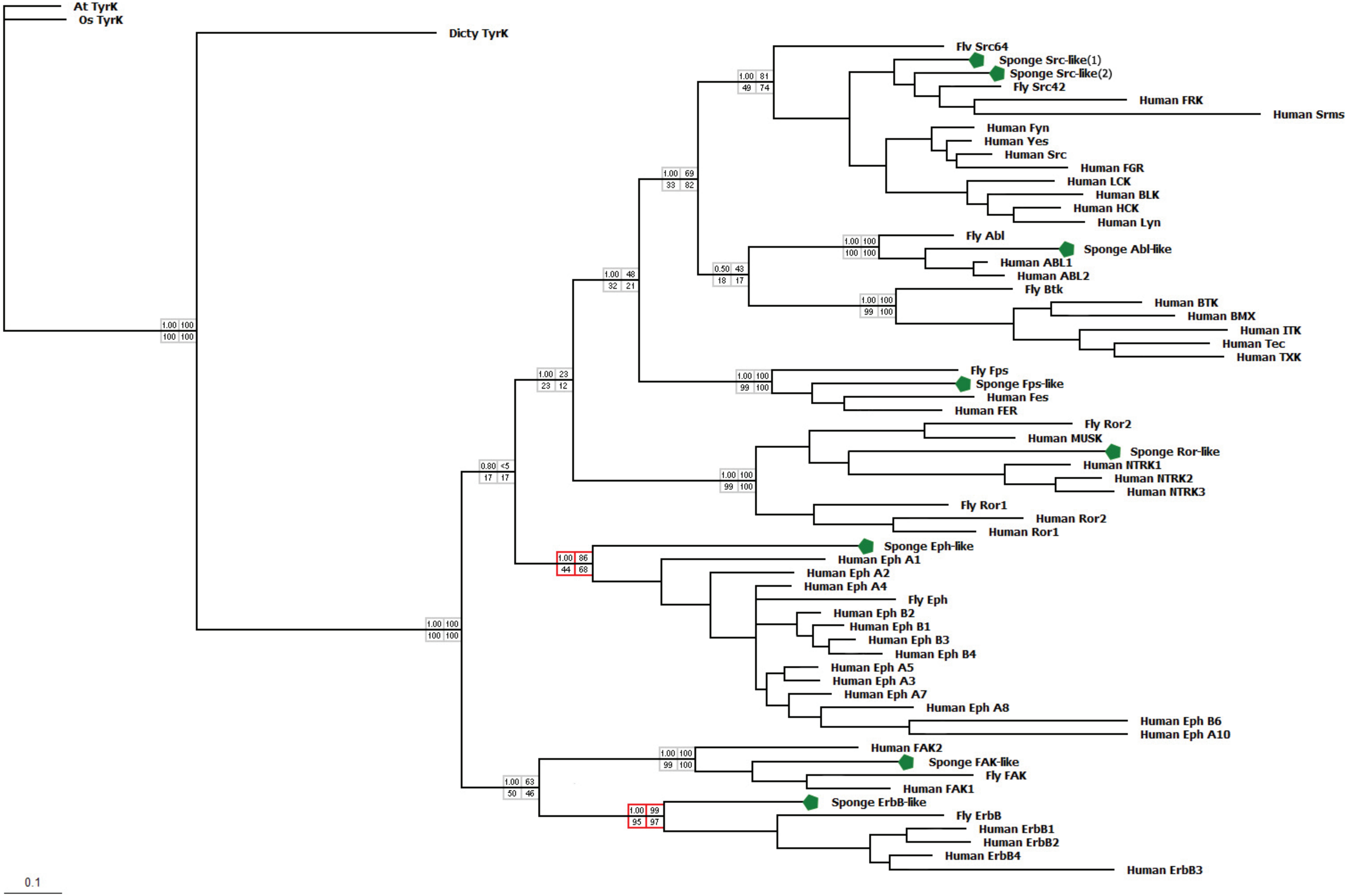

Figure S1.17. Phylogenetic analyses of Ephrin Receptor and ErbB Receptor families.

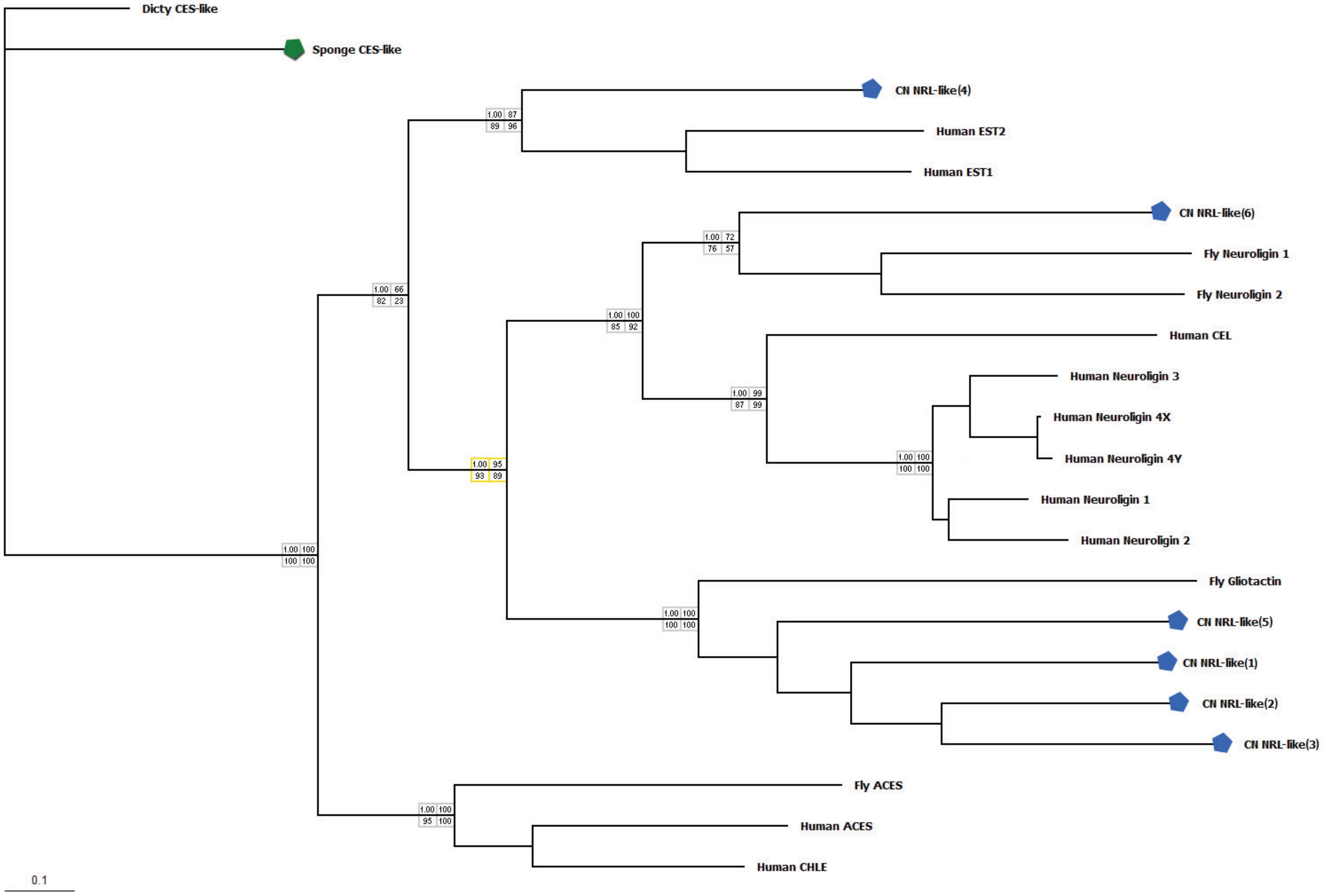

Figure S1.18. Phylogenetic analyses of Neuroligin family.

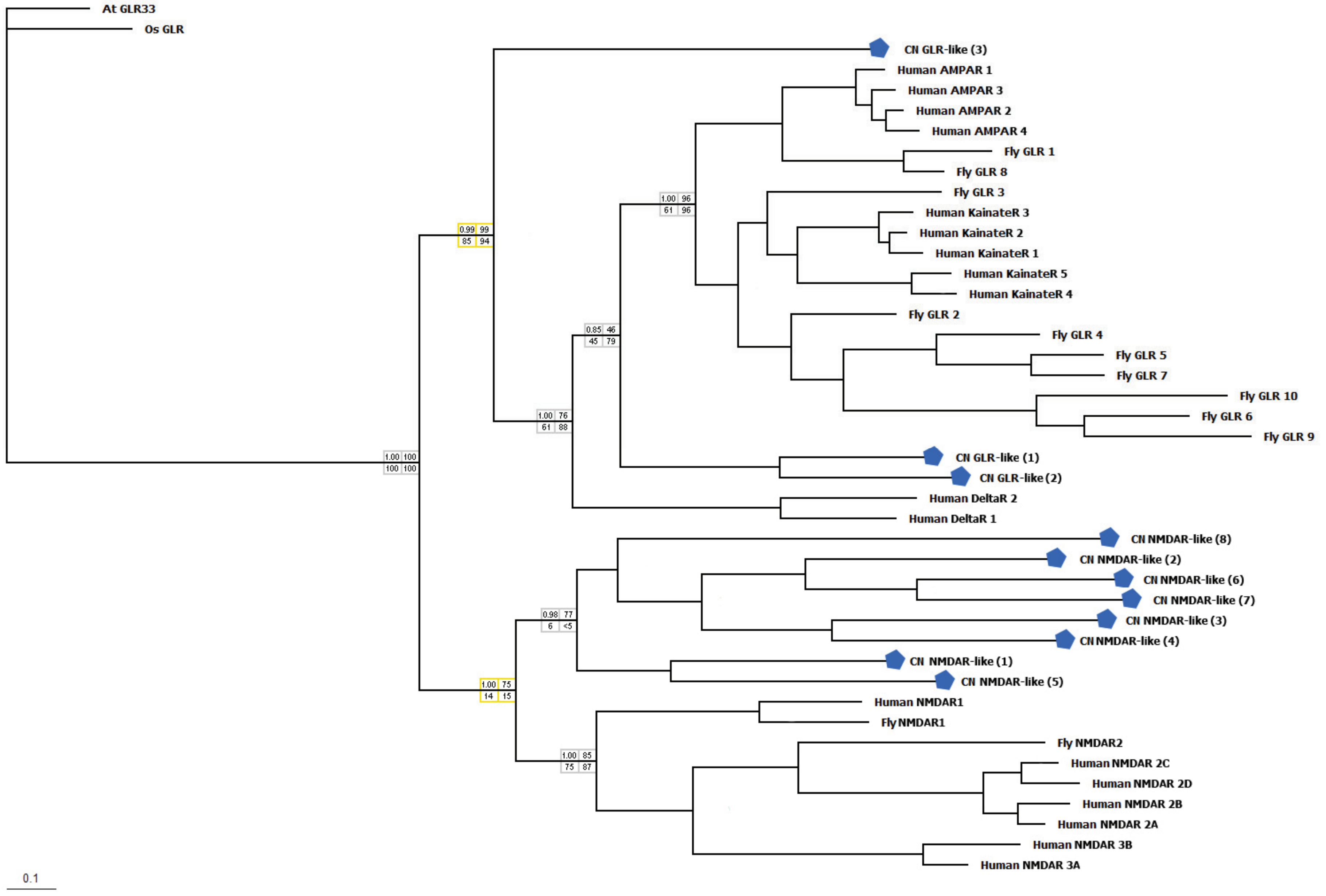

Figure S1.19. Phylogenetic analyses Ionotropic Glutamate Receptors (NMDA-R, AMPA-R, Kainate-R and related Delta-R).

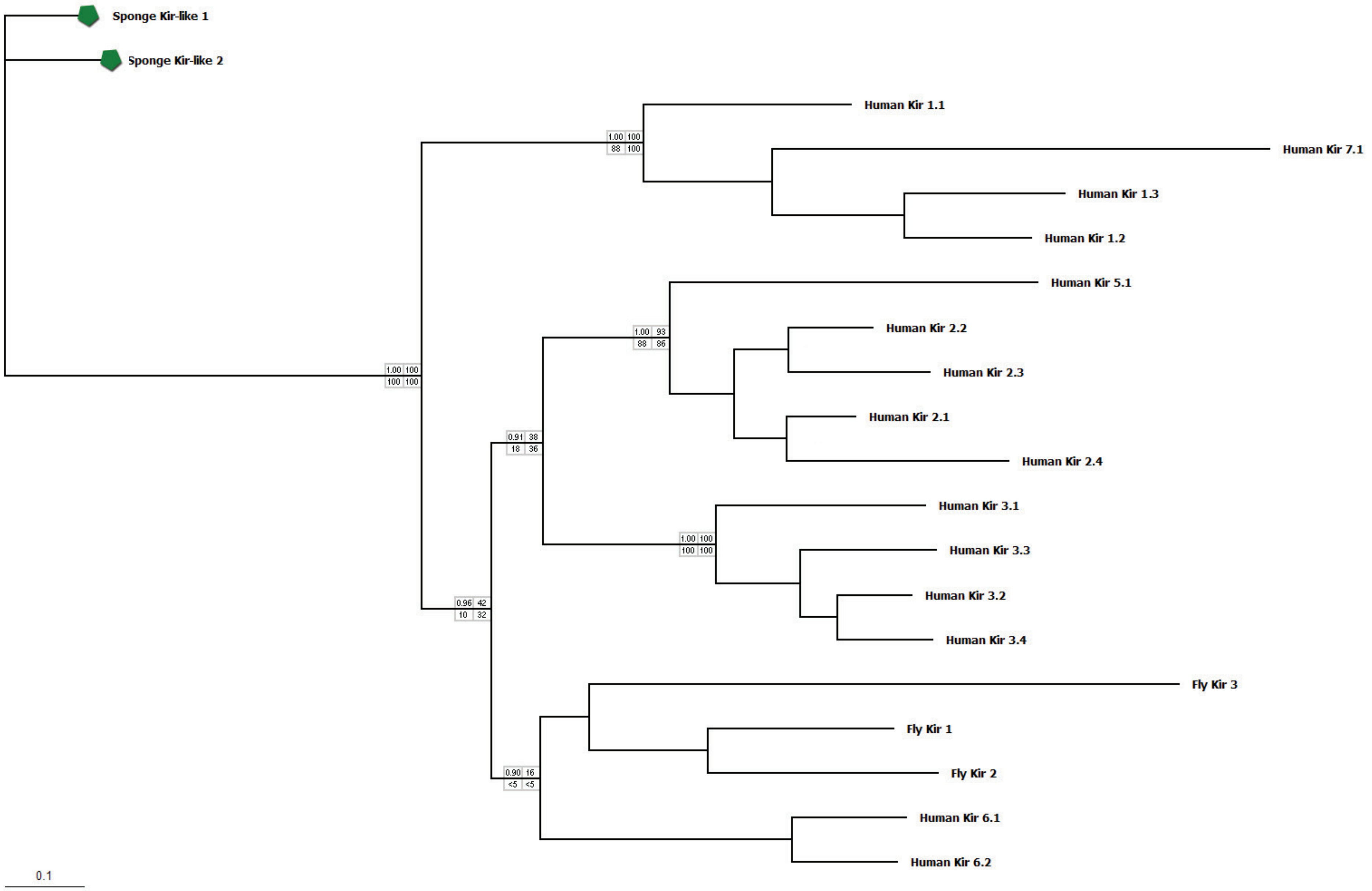

Figure S1.20. Phylogenetic analyses of K+ Channel Kir family.

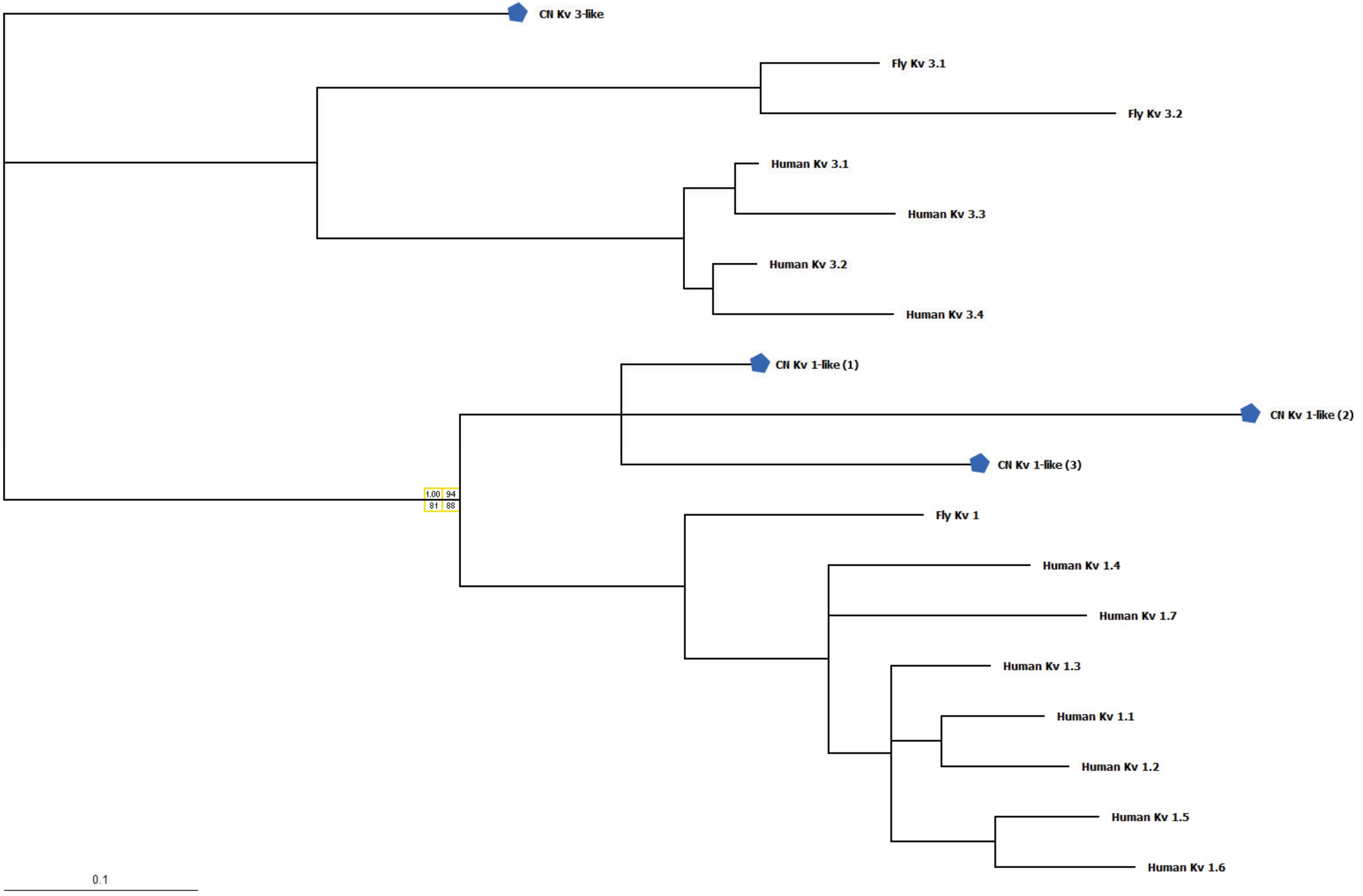

Figure S1.21. Phylogenetic analyses of K+ Channel Shaker family.

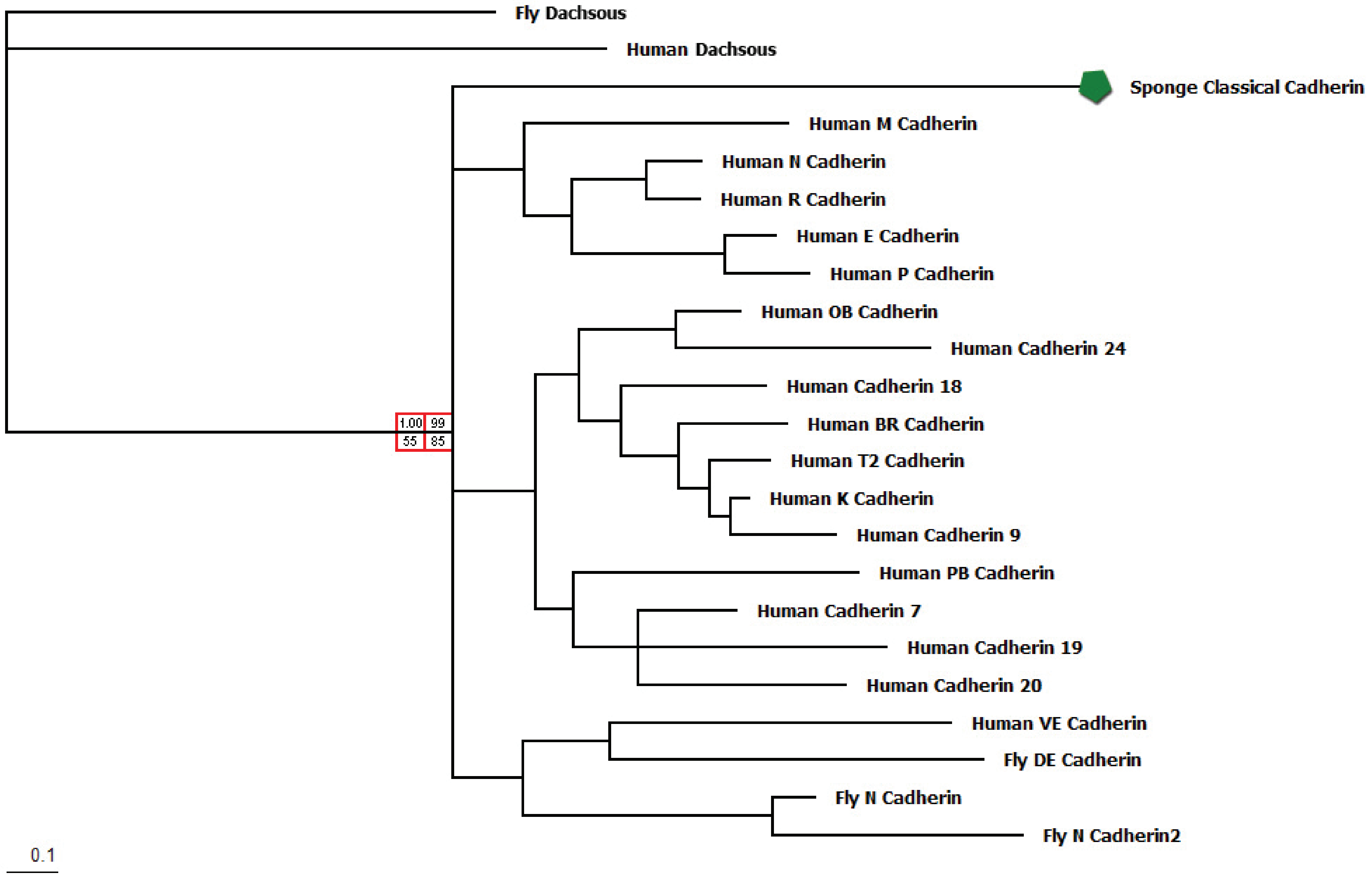

Figure S1.22. Phylogenetic analyses of Classical Cadherin family.

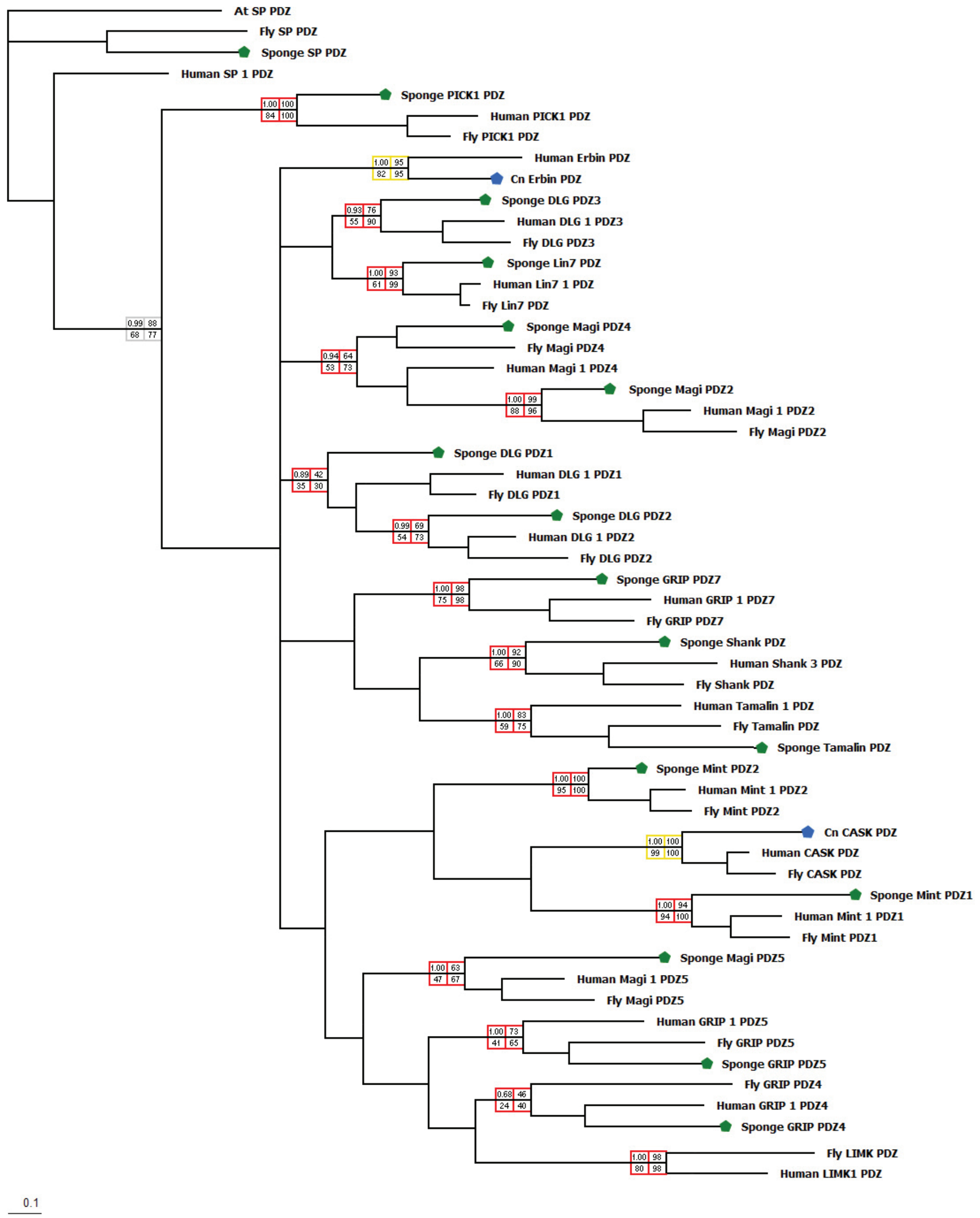

Figure S1.23. Phylogenetic analyses of DLG, Shank, GRIP, MAGI, LIN-7, LIMK, Mint, Tamalin, Erbin, NOS, PICK1, CASK families.
