# Supplementary material for: A Post-Synaptic Scaffold at the Origin of the Animal Kingdom
Source: PLoS One. 2007 Jun 6;2(6):e506. doi: 10.1371/journal.pone.0000506 (PMC1876816; doi:10.1371/journal.pone.0000506)
Supplement: Figure S2 — Domain architectures of post-synaptic gene families. Domain architectures of representative members for each gene family are displayed as SMART output. Output was manually edited for legibility for some PFAM domains, otherwise it is presented as SMART prediction. Abbreviations used in displays are: Sponge, Amphimedon queenslandica; CN, Nematostella vectensis; Human, Homo sapiens; Fly, Drosophila melanogaster; Beetle, Tribolium castaneum. (3.66 MB PDF) [file pone.0000506.s002.pdf]

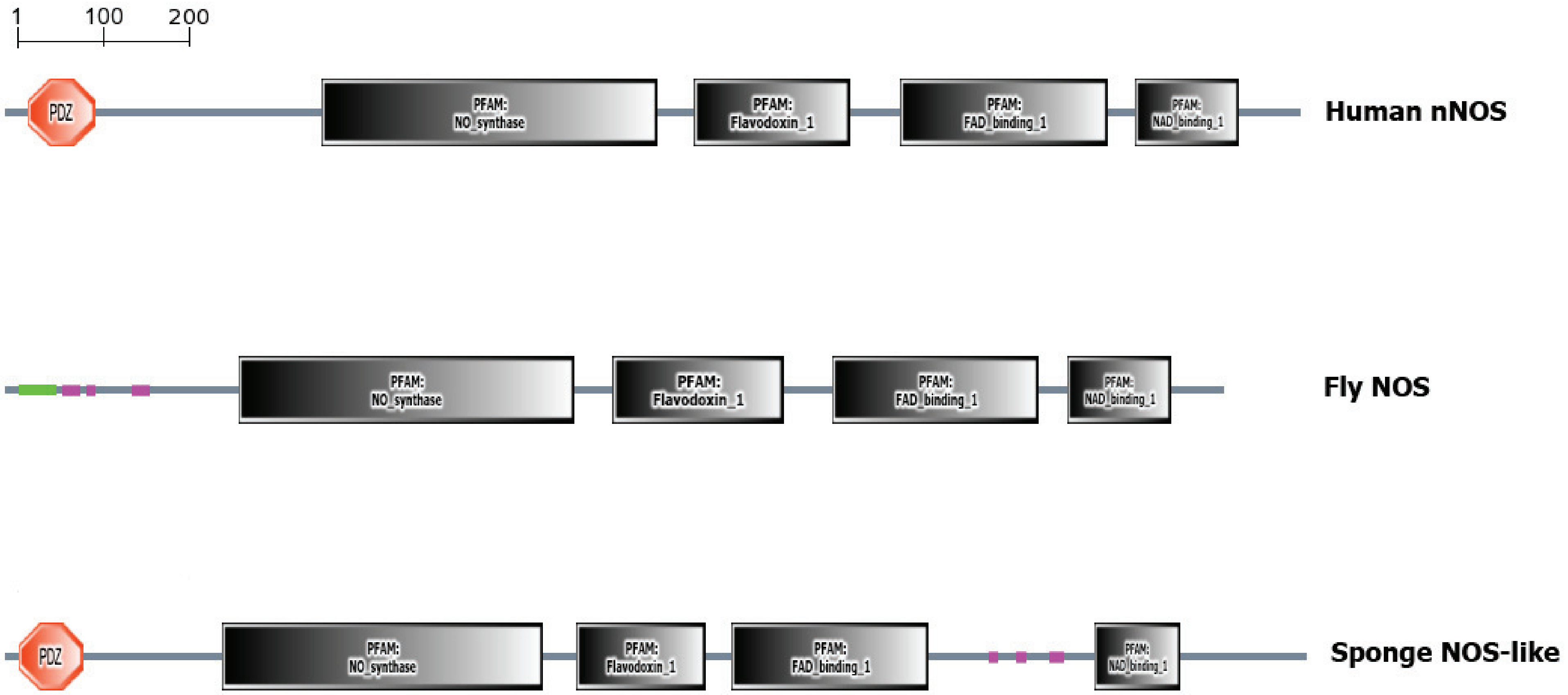

Figure S2.1. Domain architecture display of NOS family.

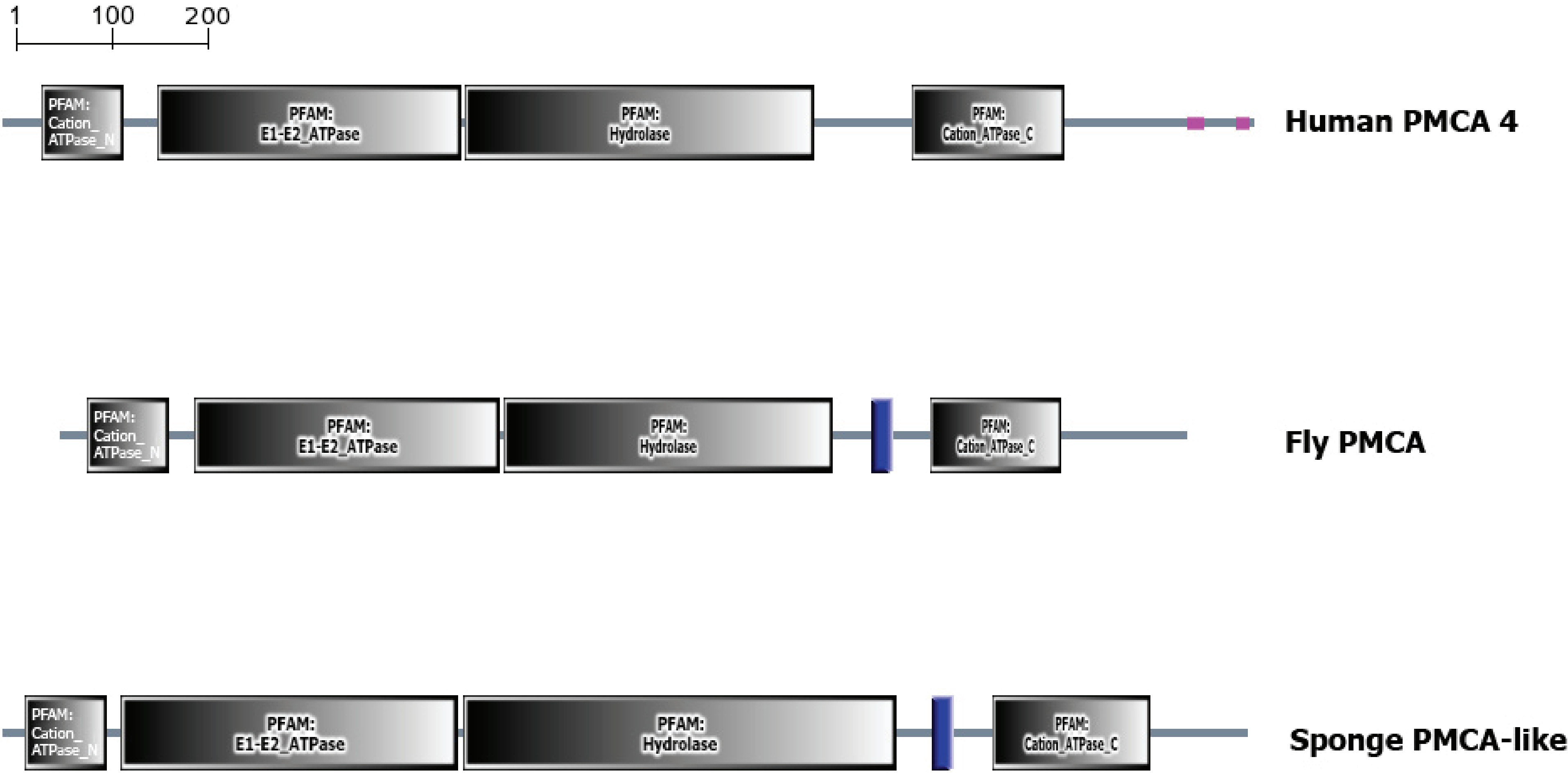

Figure S2.2. Domain architecture display of PMCA family.

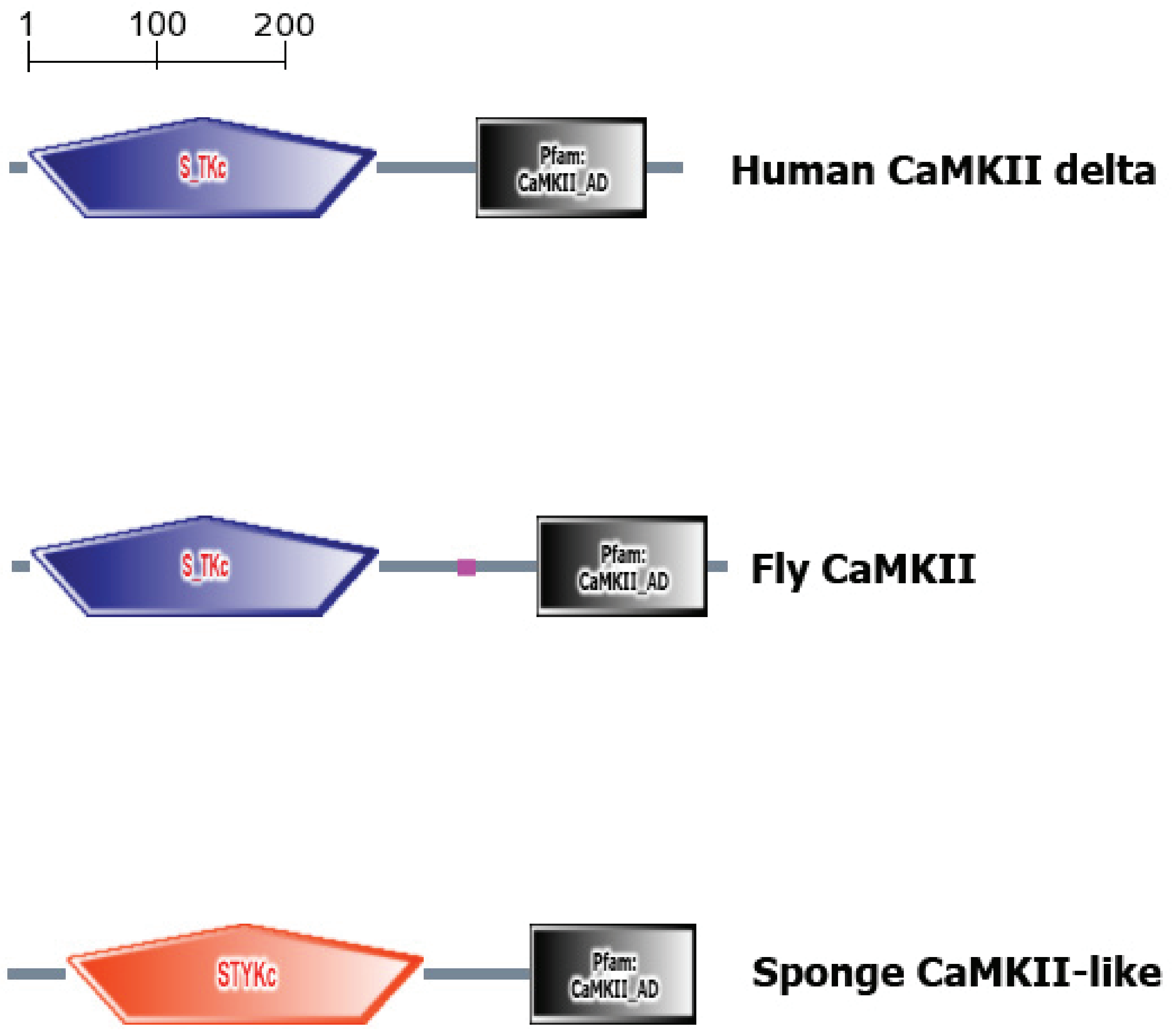

Figure S2.3.a. Domain architecture display of CaMKII family.

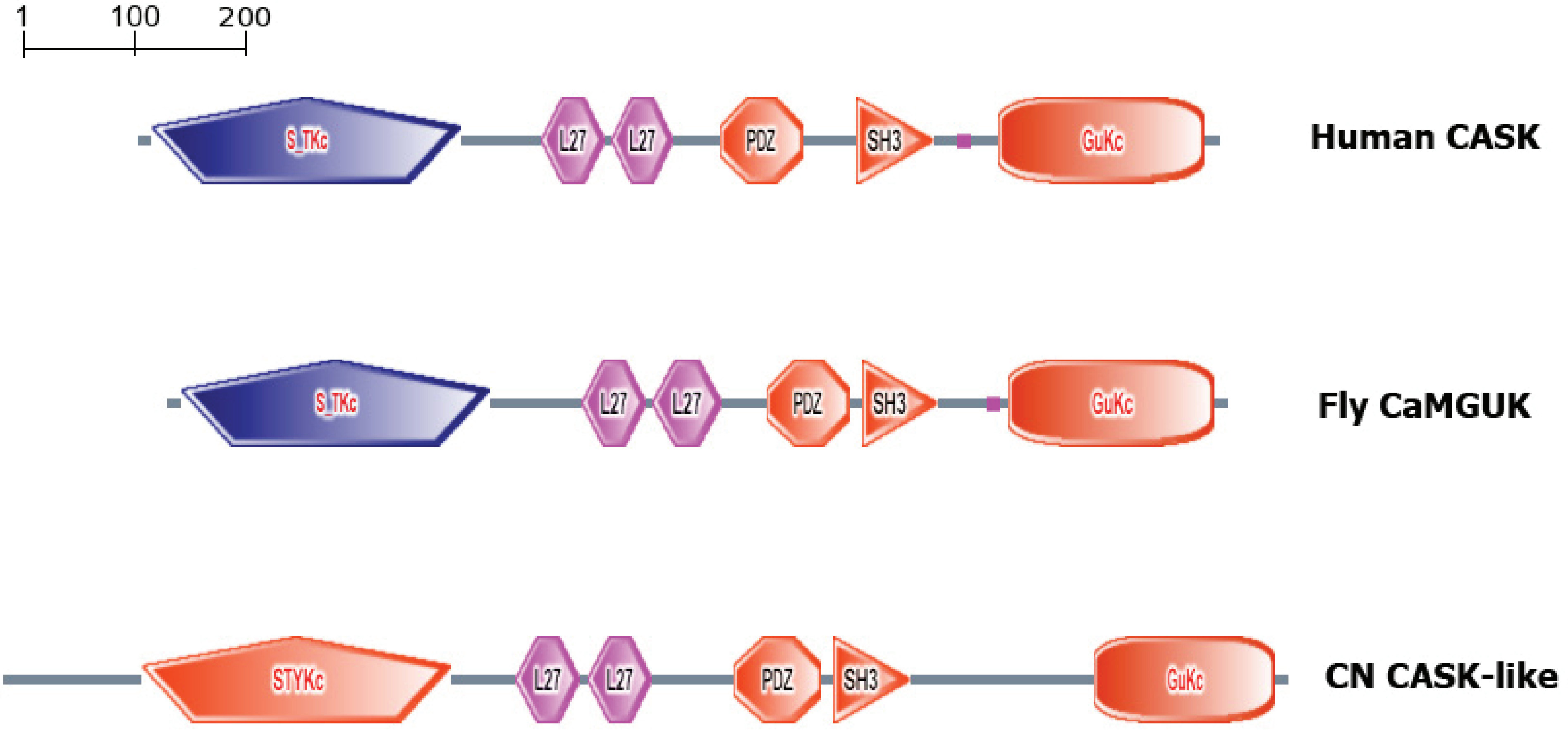

Figure S2.3.b. Domain architecture display of CASK family.

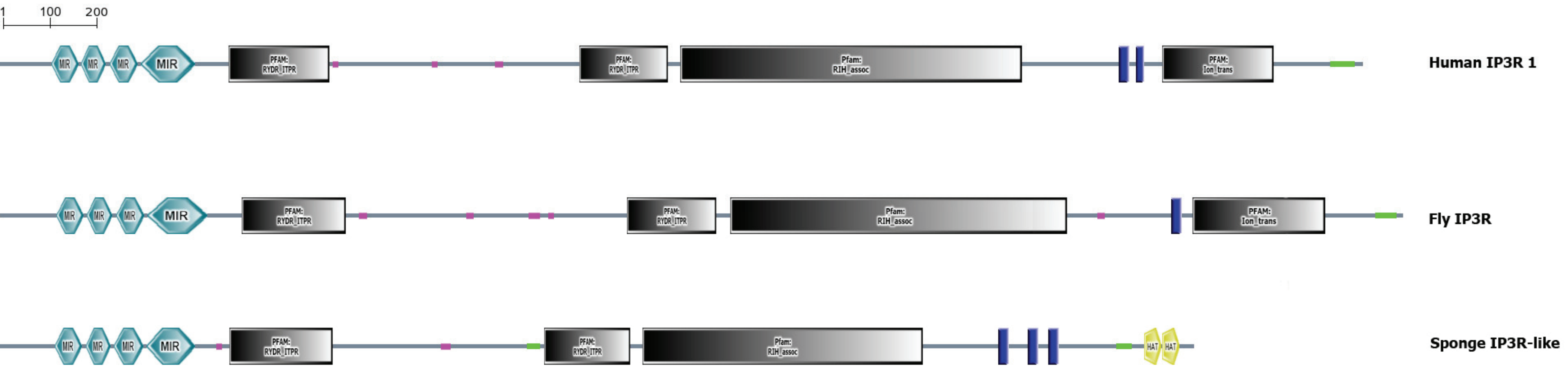

Figure S2.4. Domain architecture display of IP3R family.

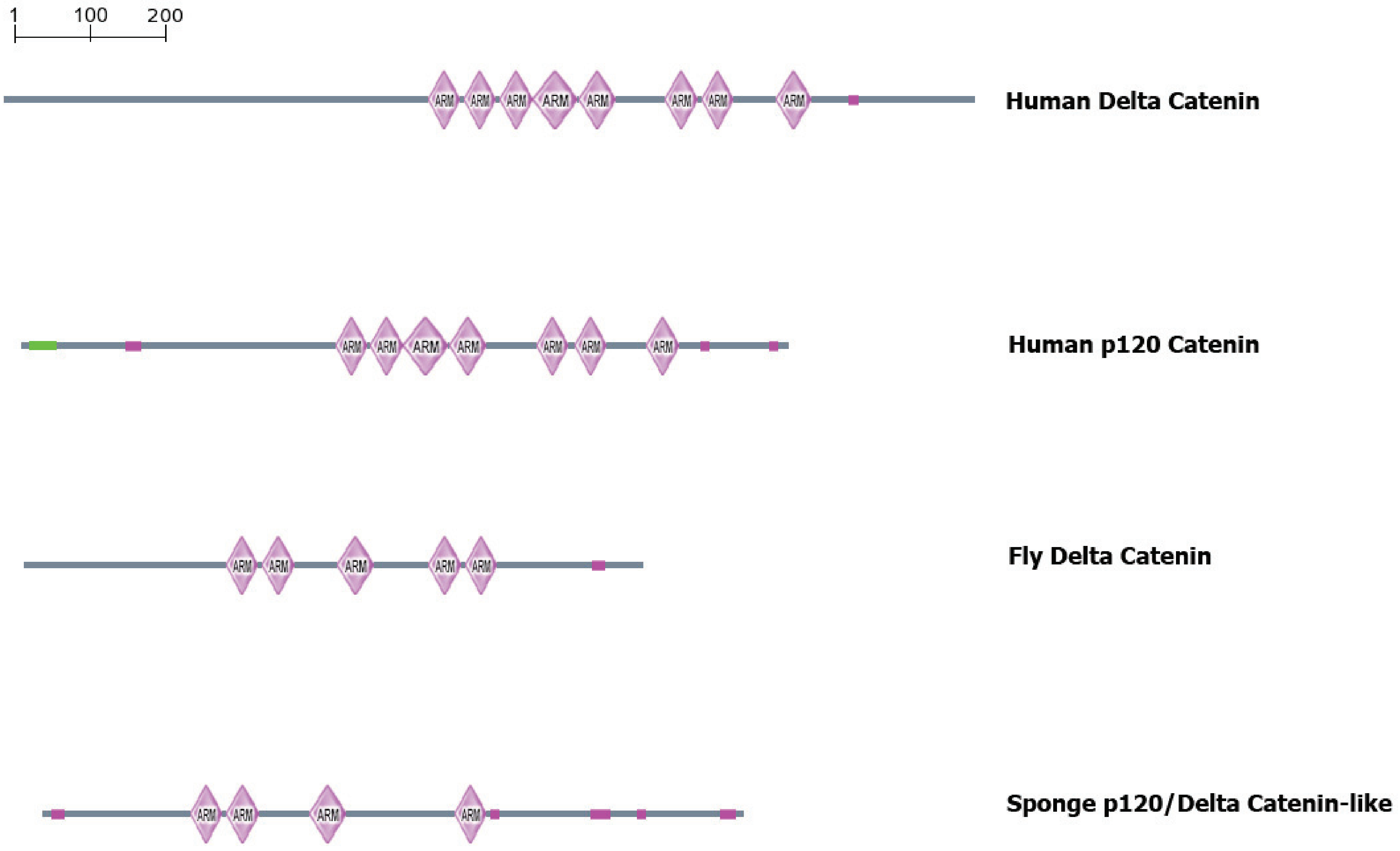

Figure S2.5.a. Domain architecture display of Delta Catenin family.

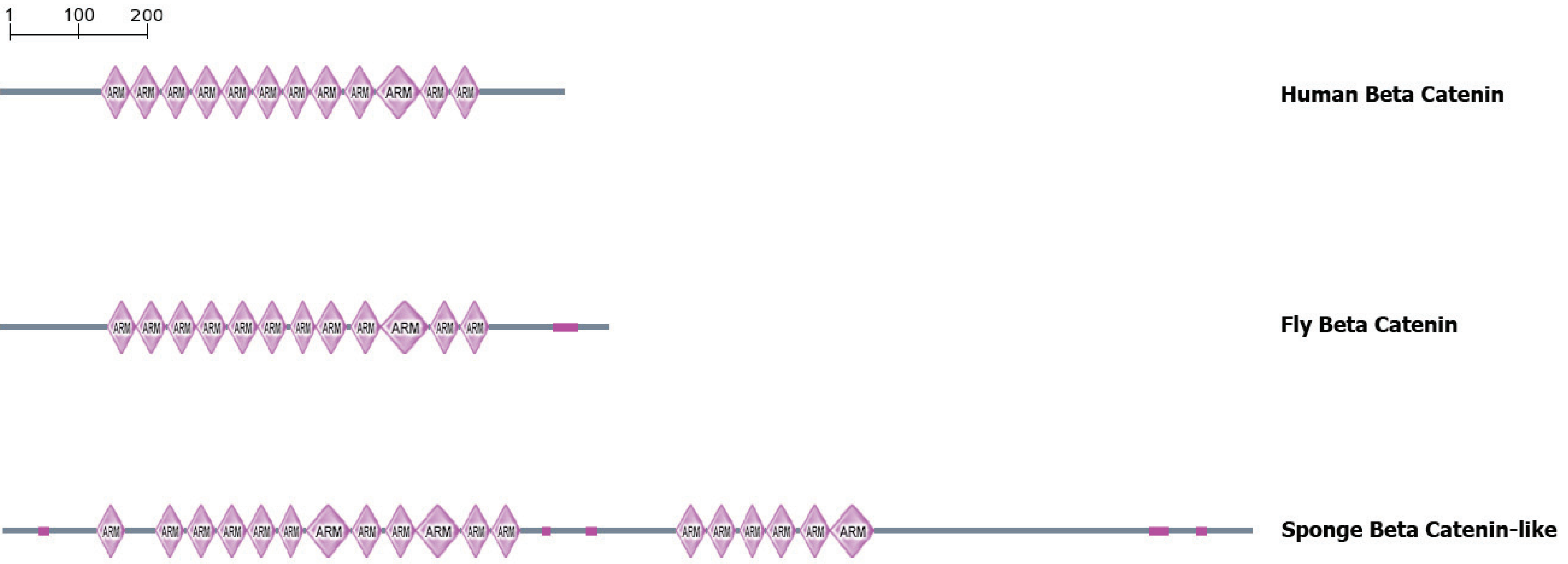

Figure S2.5.b. Domain architecture display of Beta Catenin family.

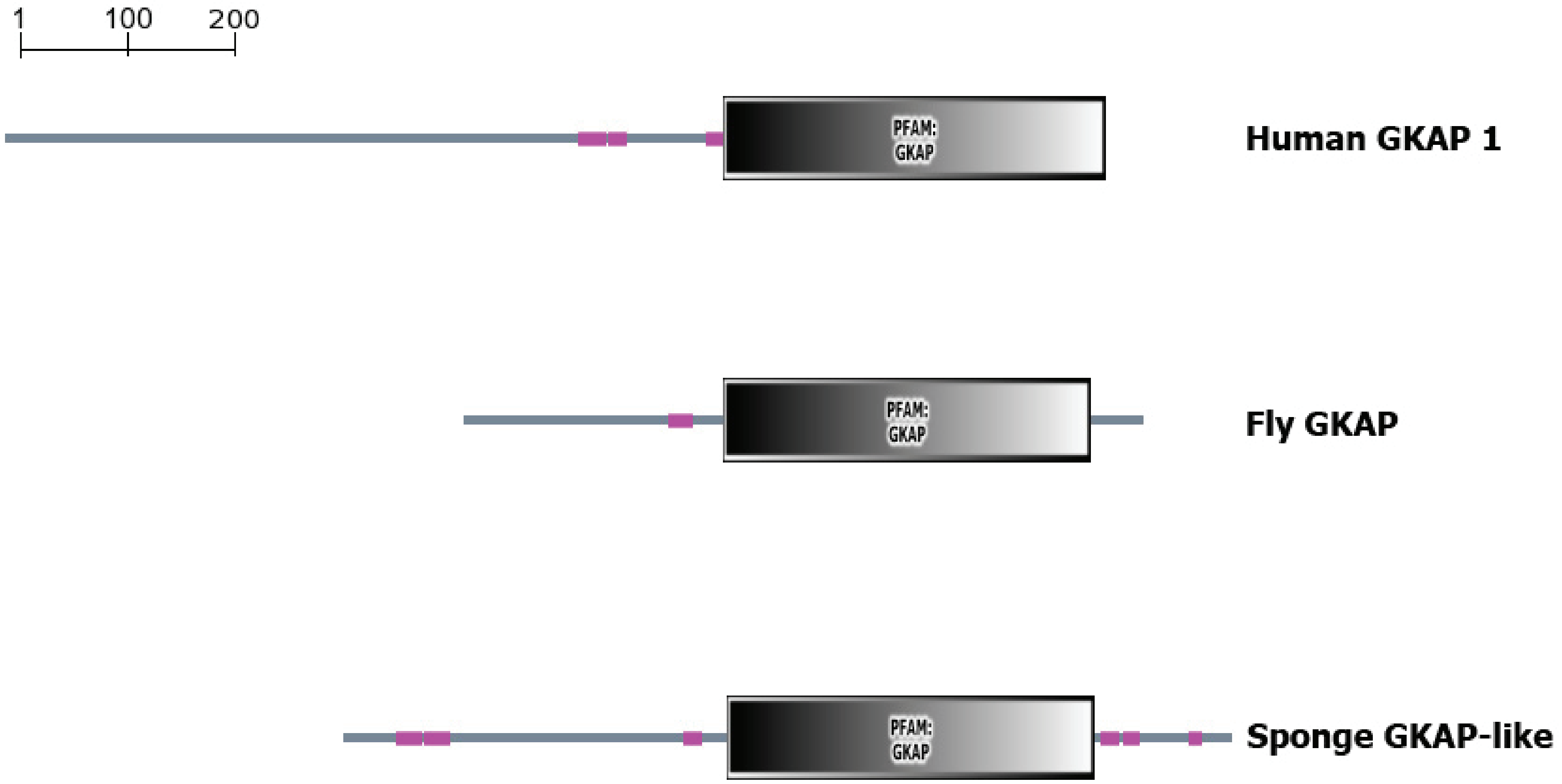

Figure S2.6. Domain architecture display of GKAP family.

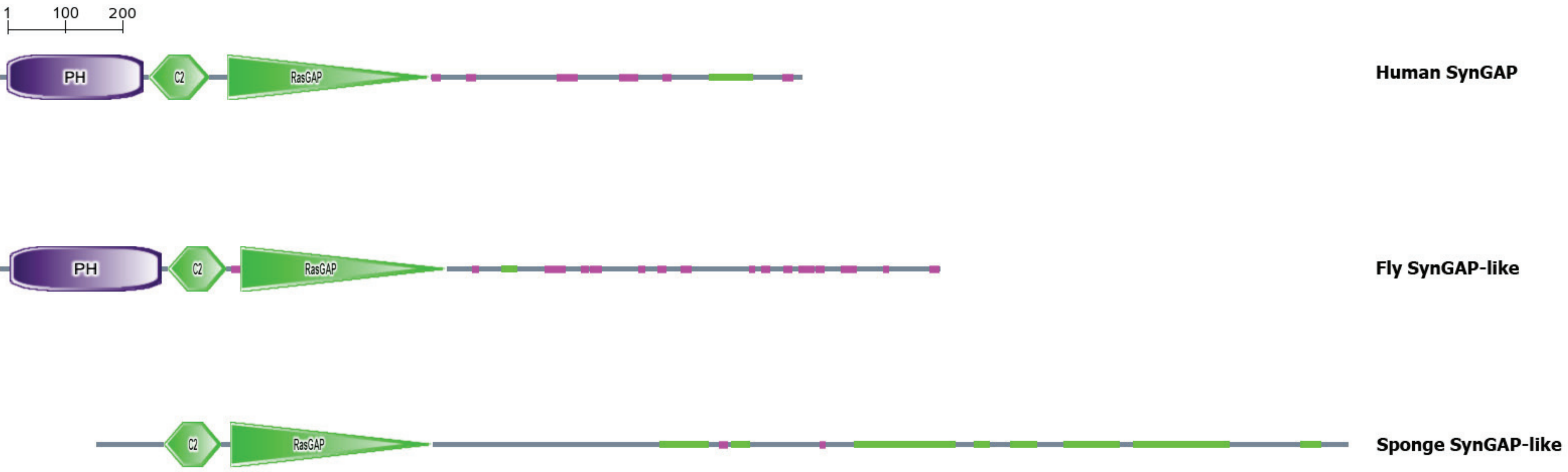

Figure S2.7. Domain architecture display of SynGAP family.

Figure S2.8. Domain architecture of CRIPT family is not shown due to no domains found by SMART.

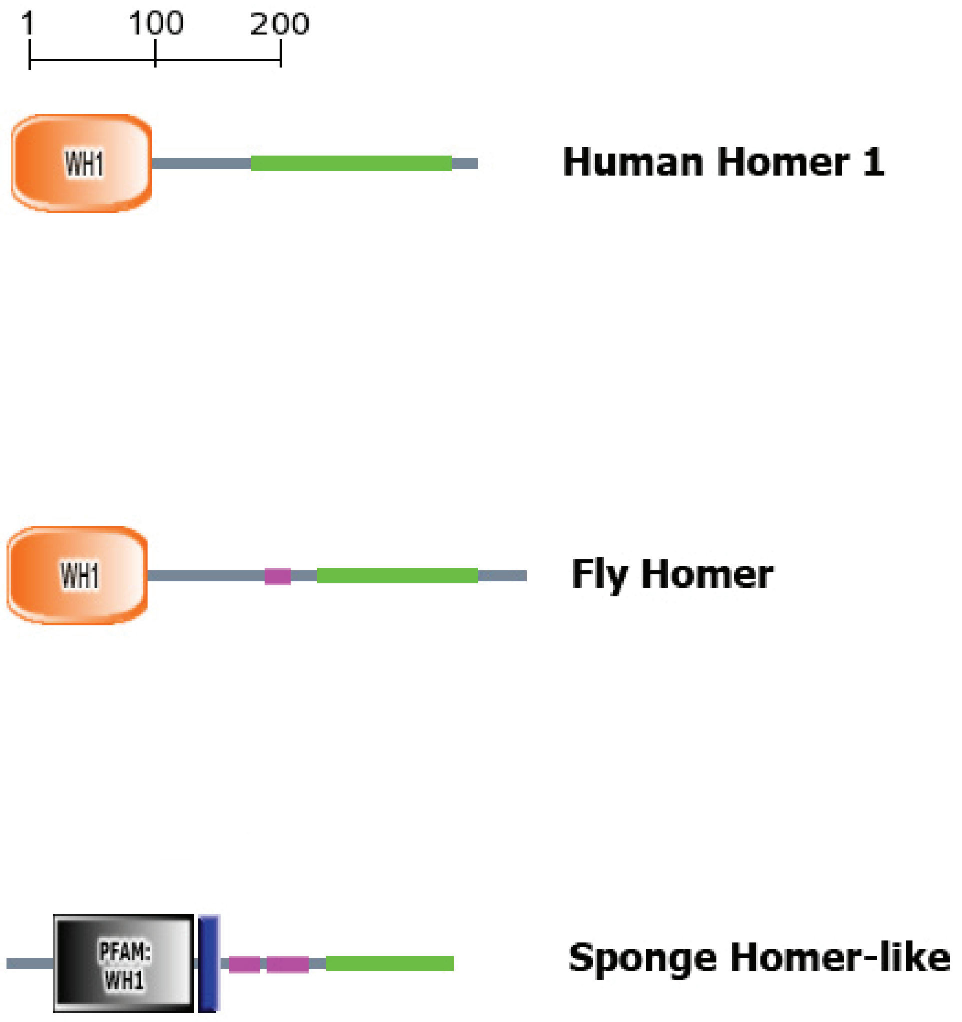

Figure S2.9. Domain architecture display of Homer family.

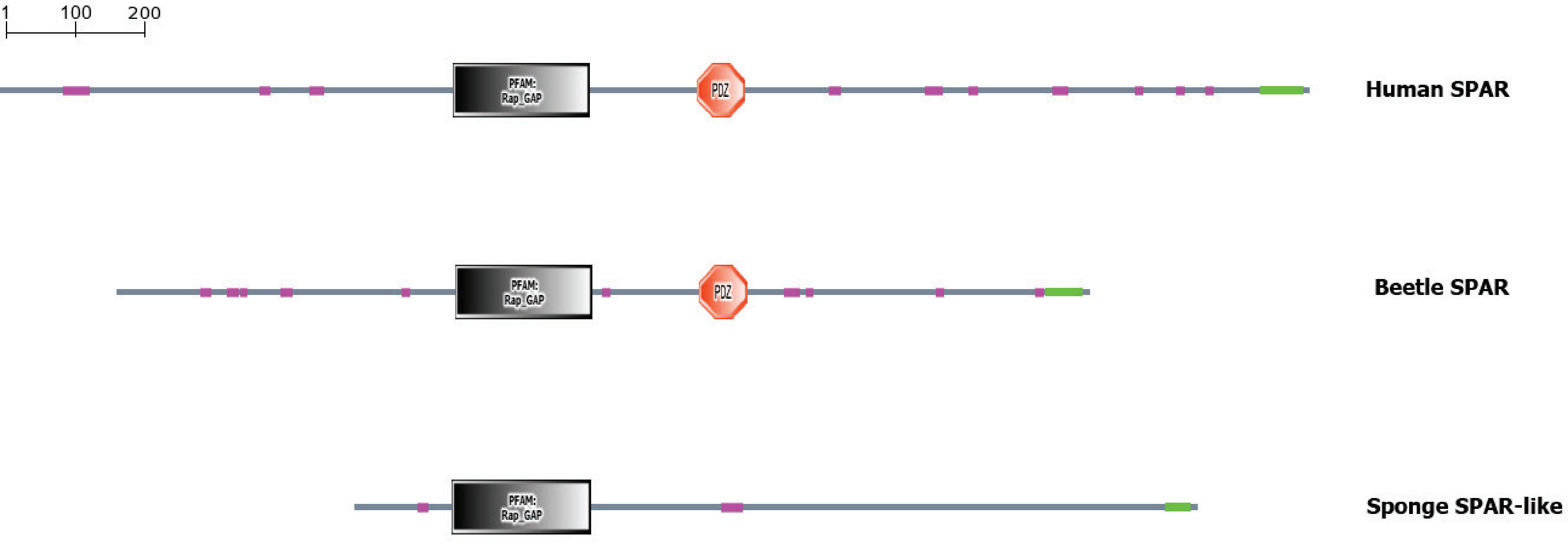

Figure S2.10. Domain architecture display of SPAR family.

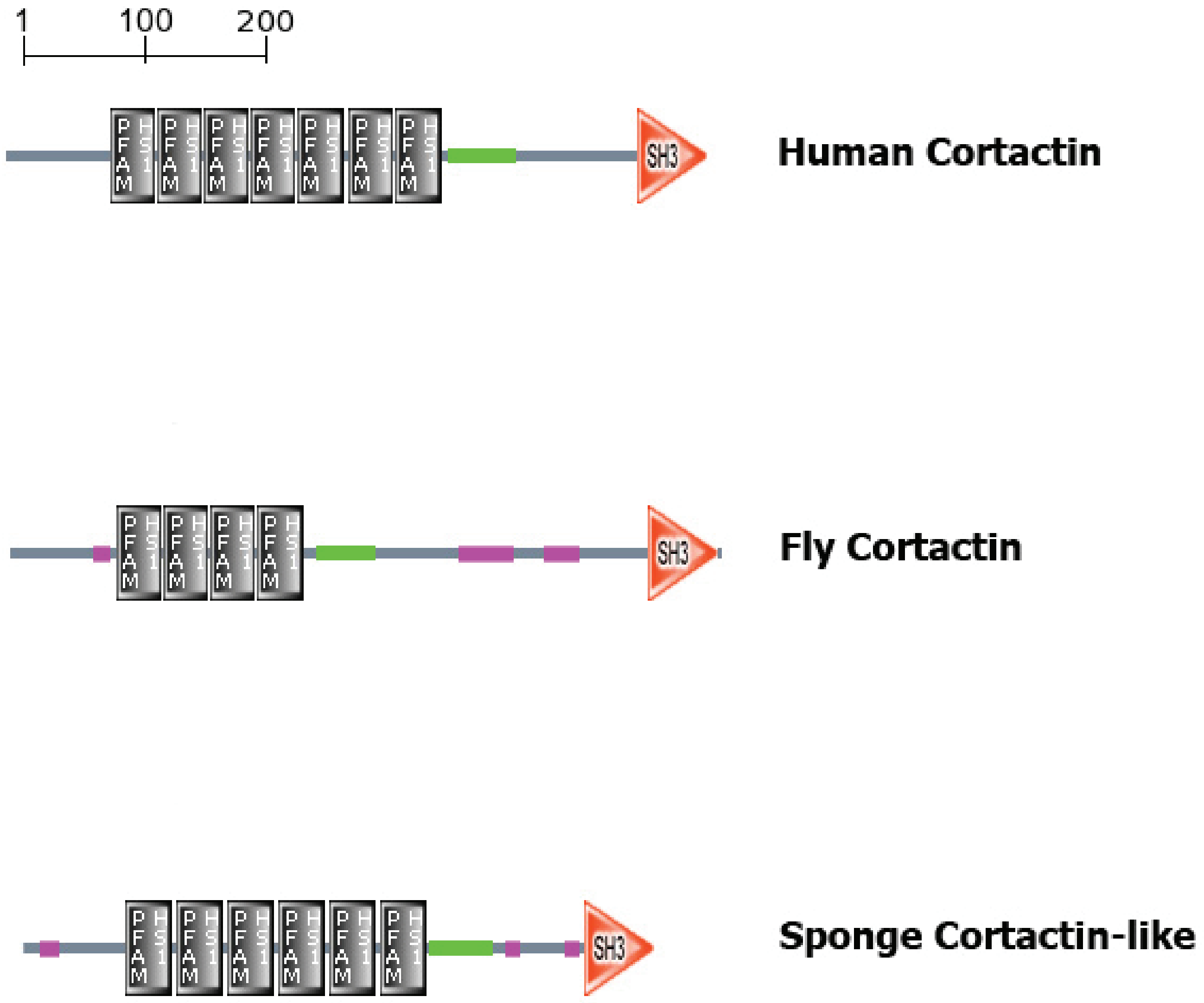

Figure S2.11. Domain architecture display of Cortactin family.

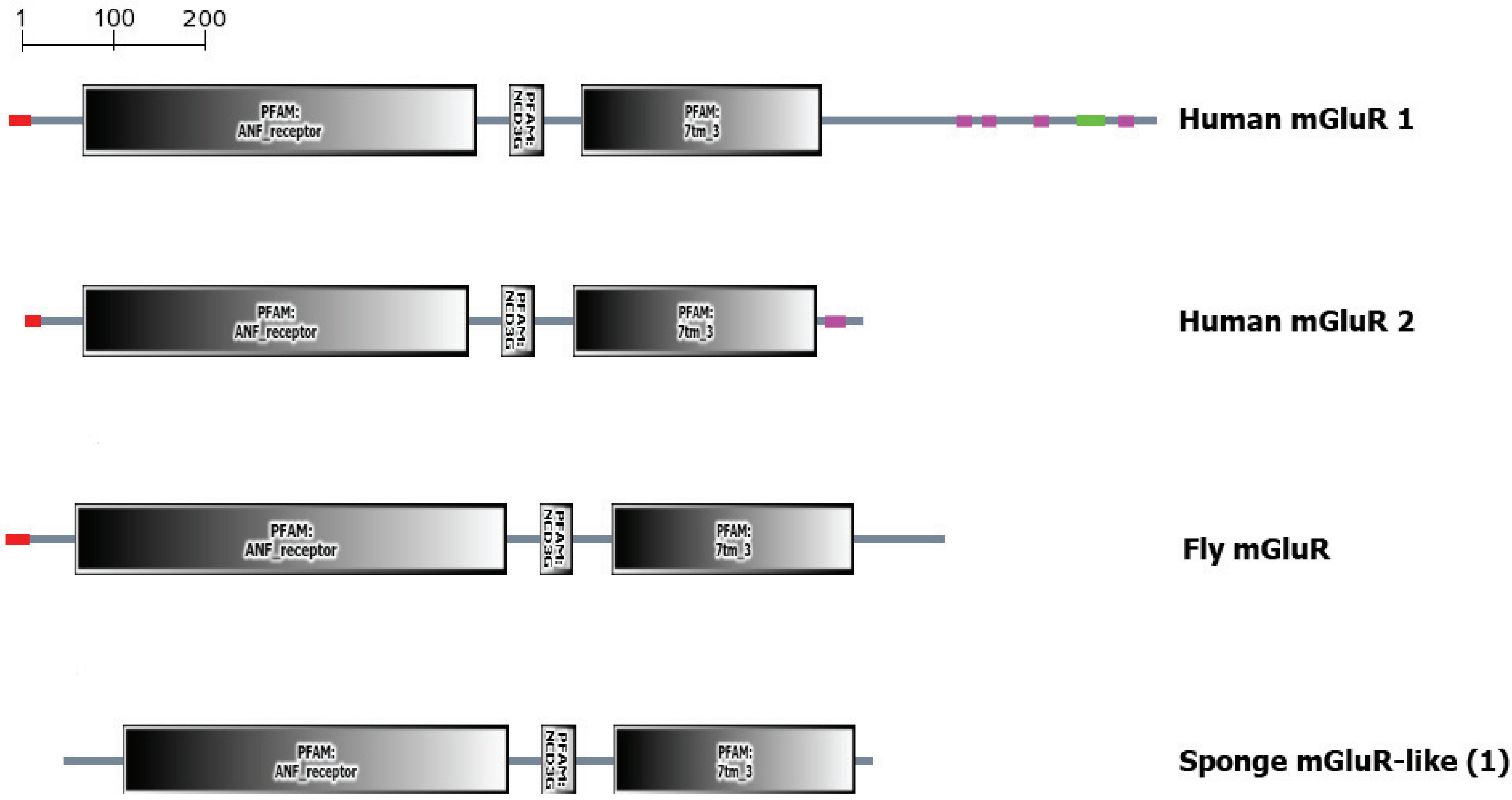

Figure S2.12.a. Domain architecture display of Metabotropic GluR family.

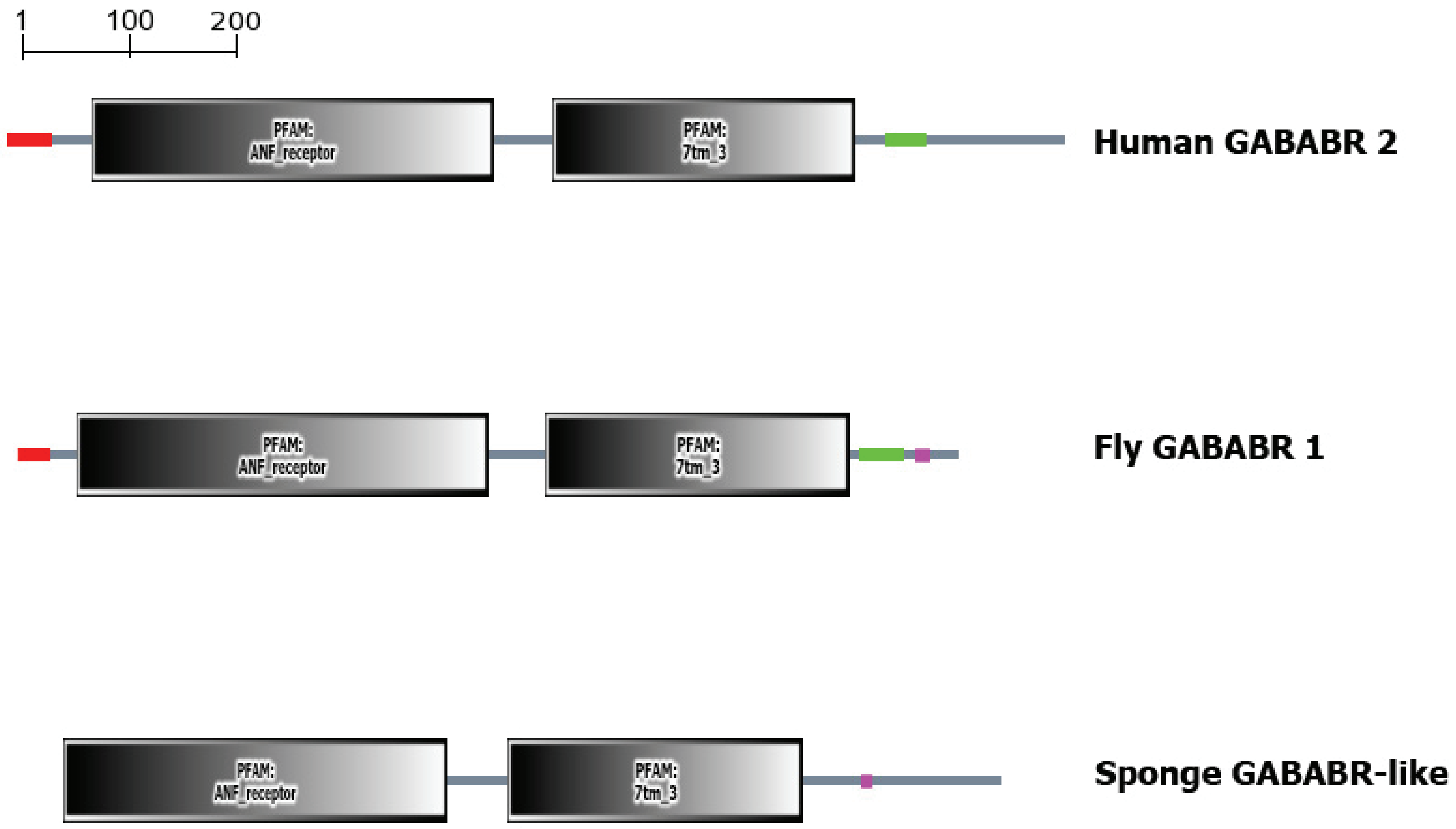

Figure S2.12.b. Domain architecture display of GABAB Receptor family.

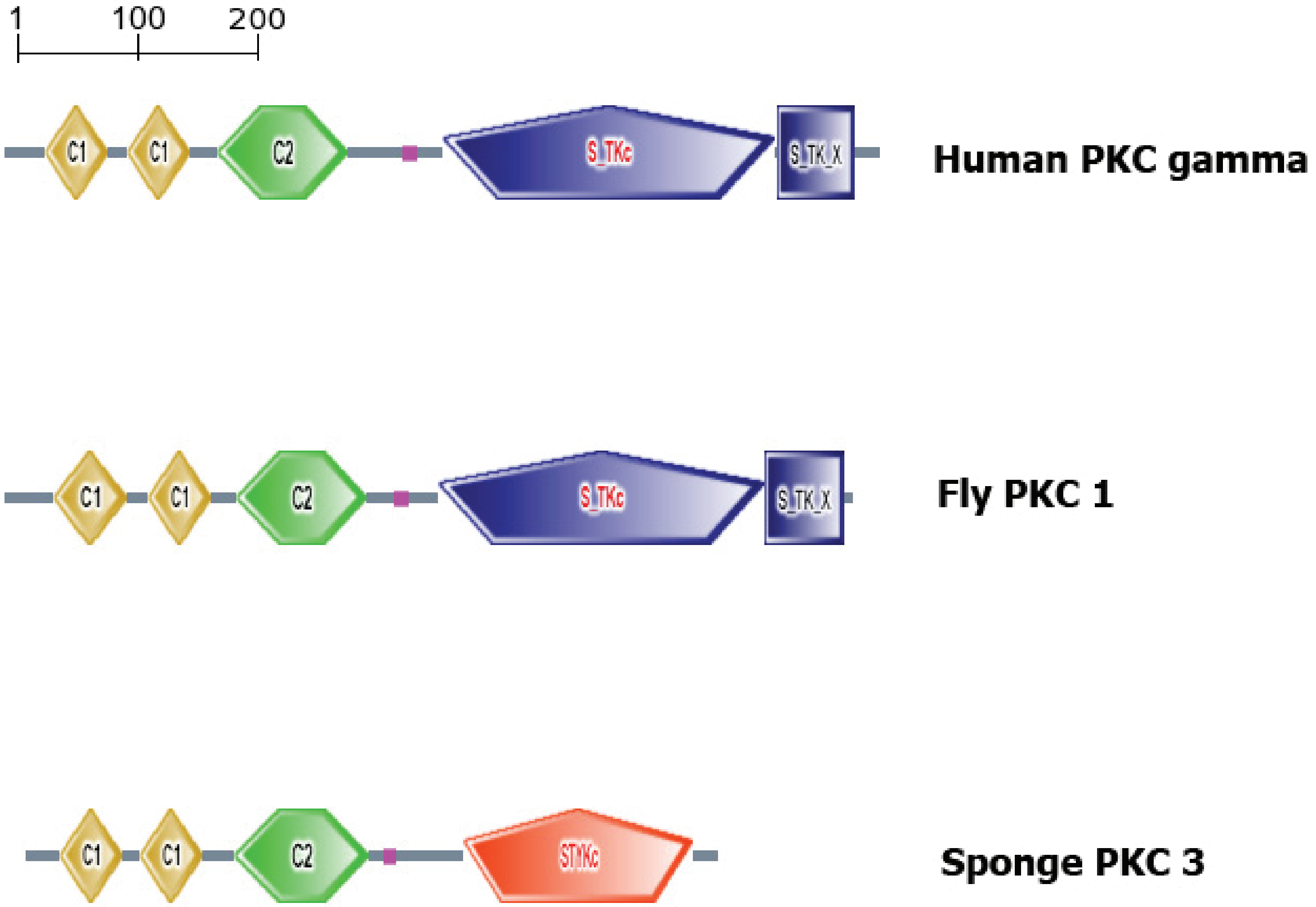

Figure S2.13. Domain architecture display of PKC family.

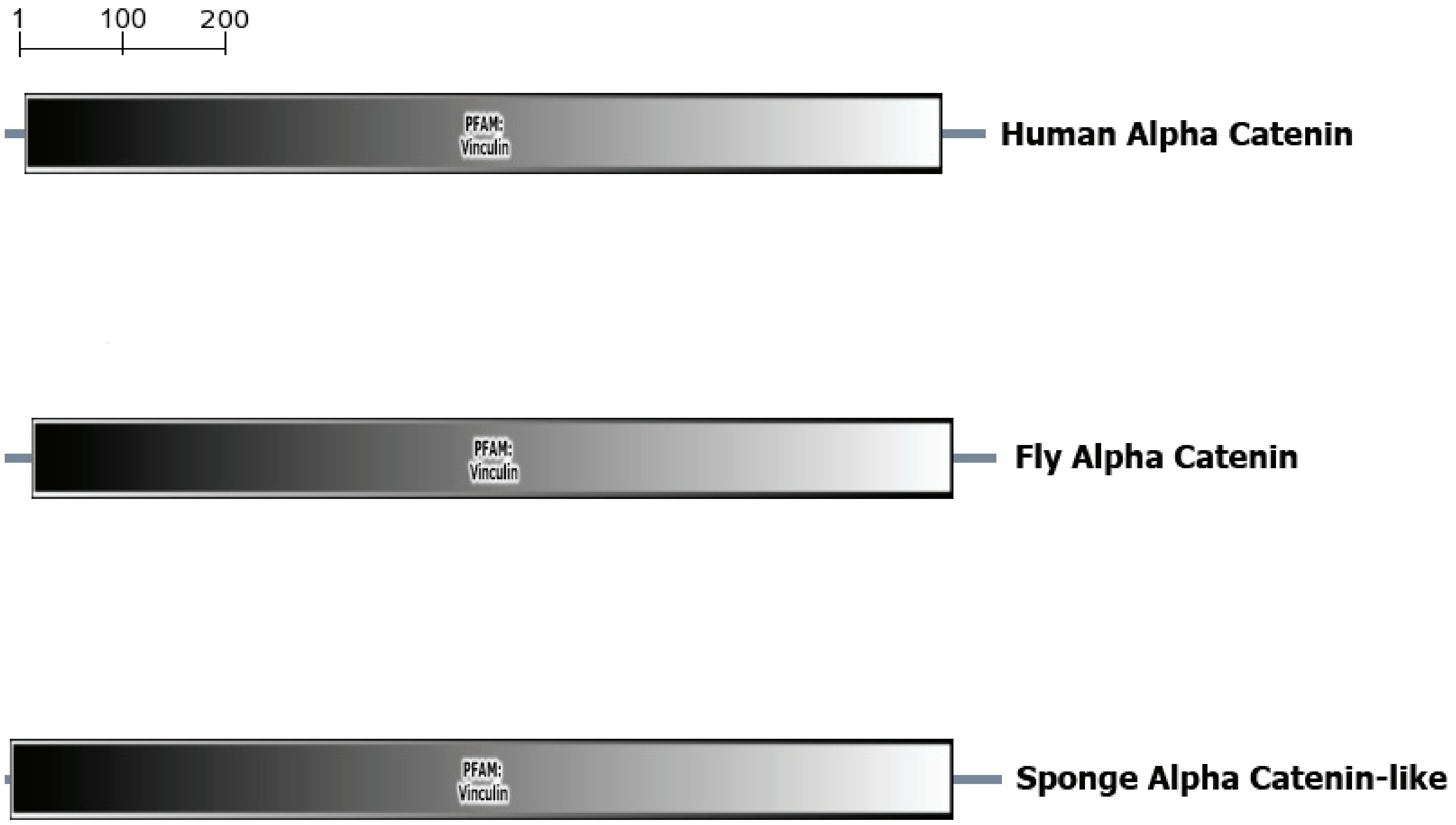

Figure S2.14. Domain architecture display of Alpha Catenin family.

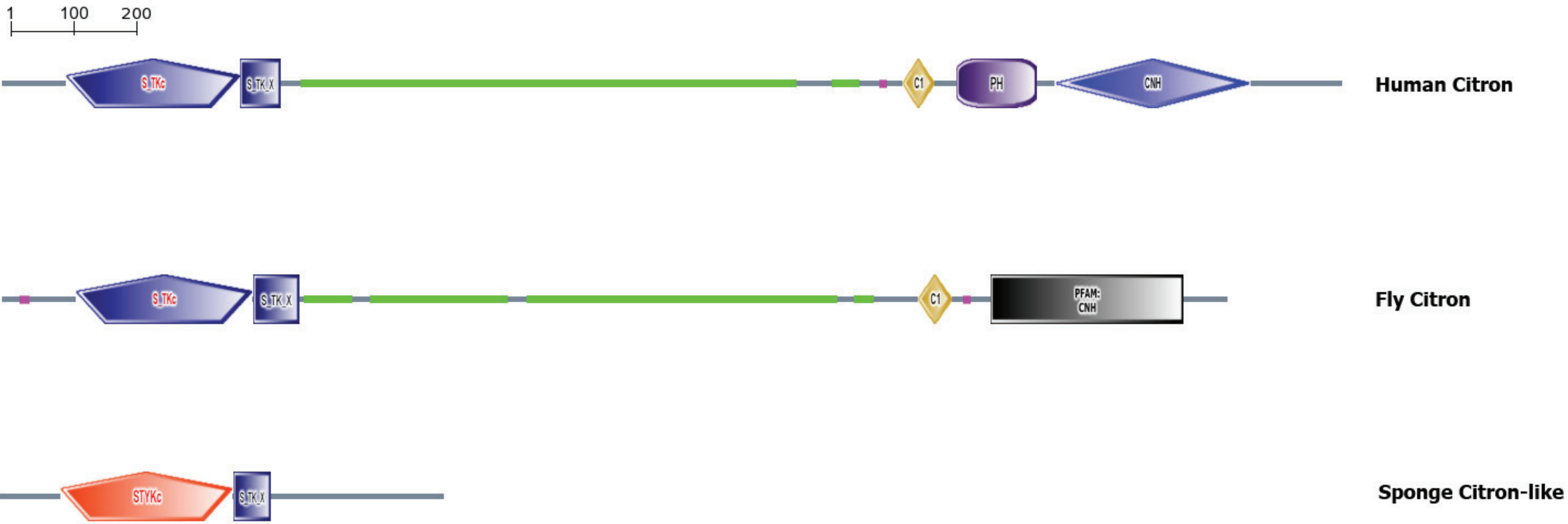

Figure S2.15. Domain architecture display of Citron family.

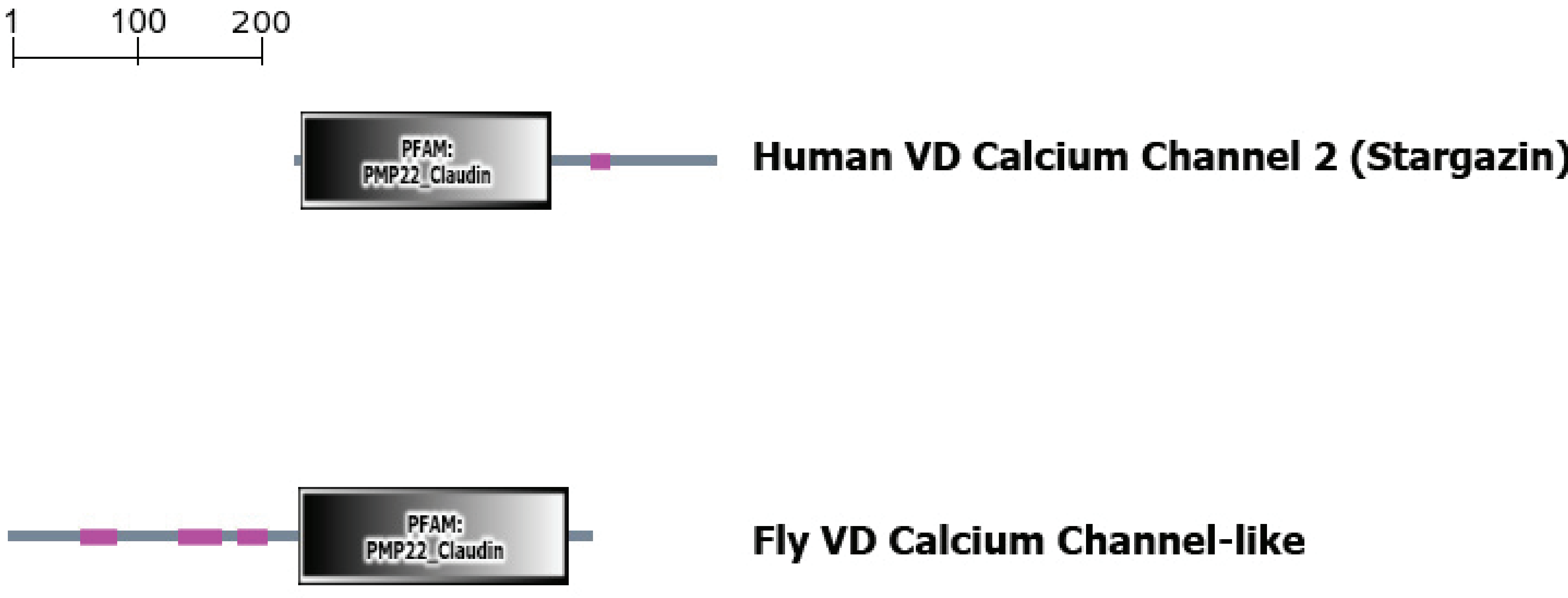

Figure S2.16. Domain architecture display of Stargazin family.

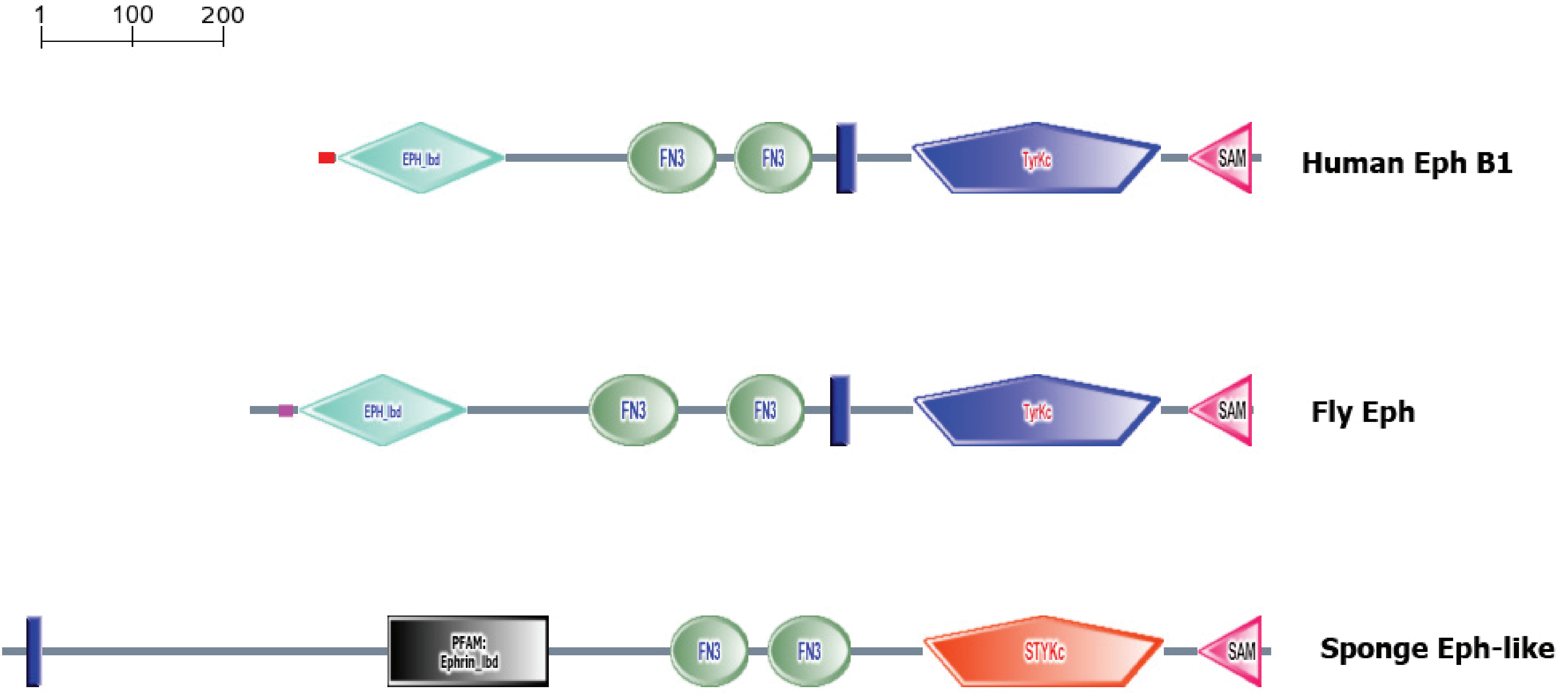

Figure S2.17.a. Domain architecture display of Ephrin Receptor family.

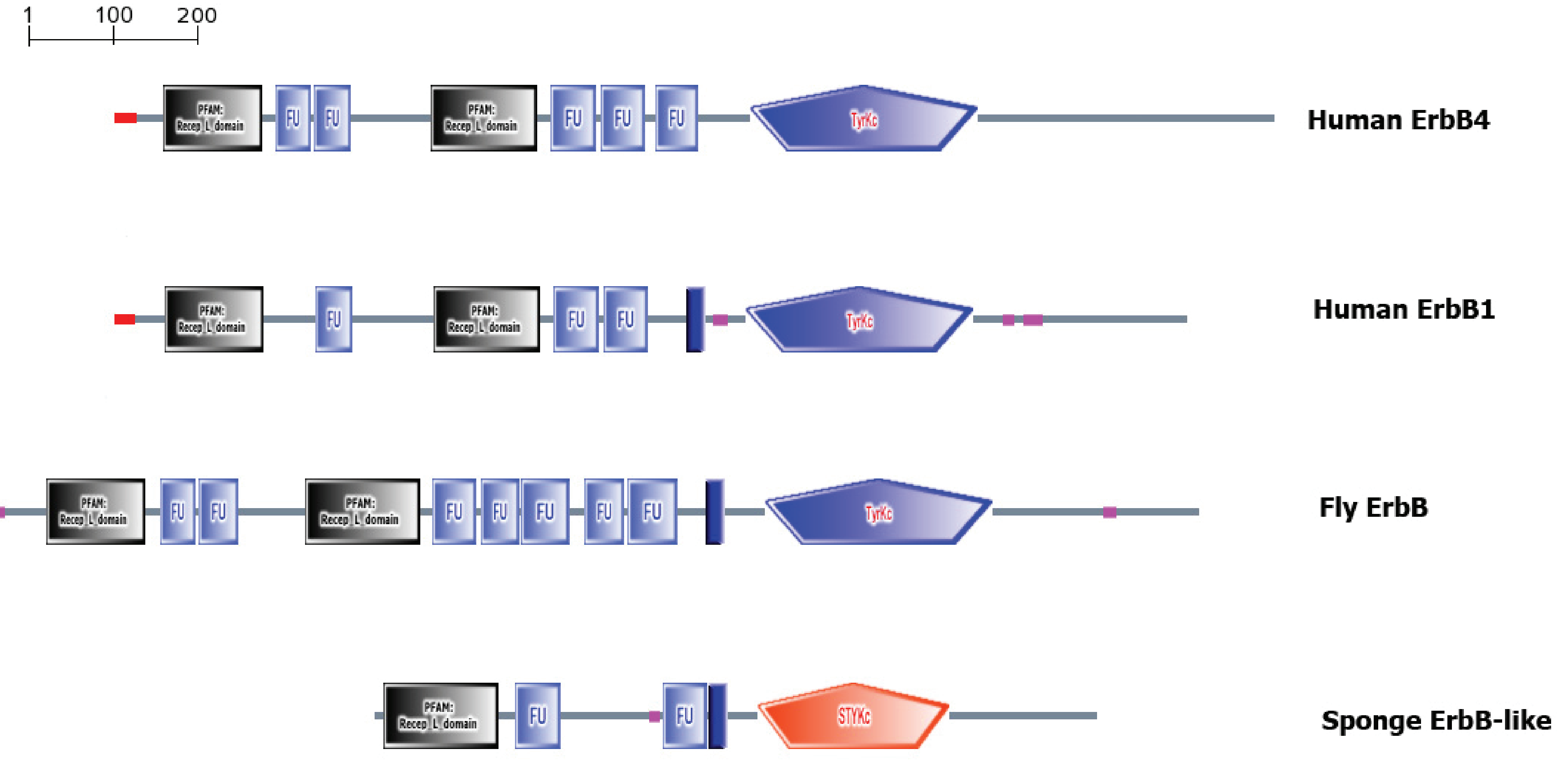

Figure S2.17.b. Domain architecture display of ErbB Receptor family.

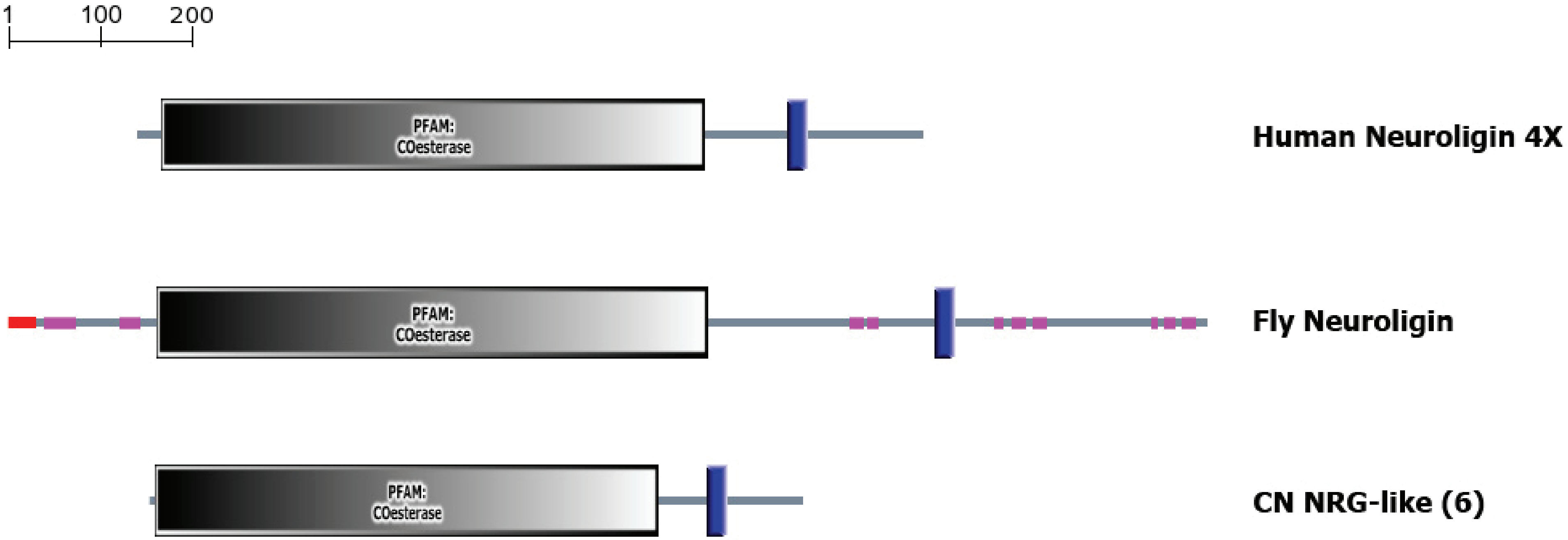

Figure S2.18. Domain architecture display of Neuroligin family.

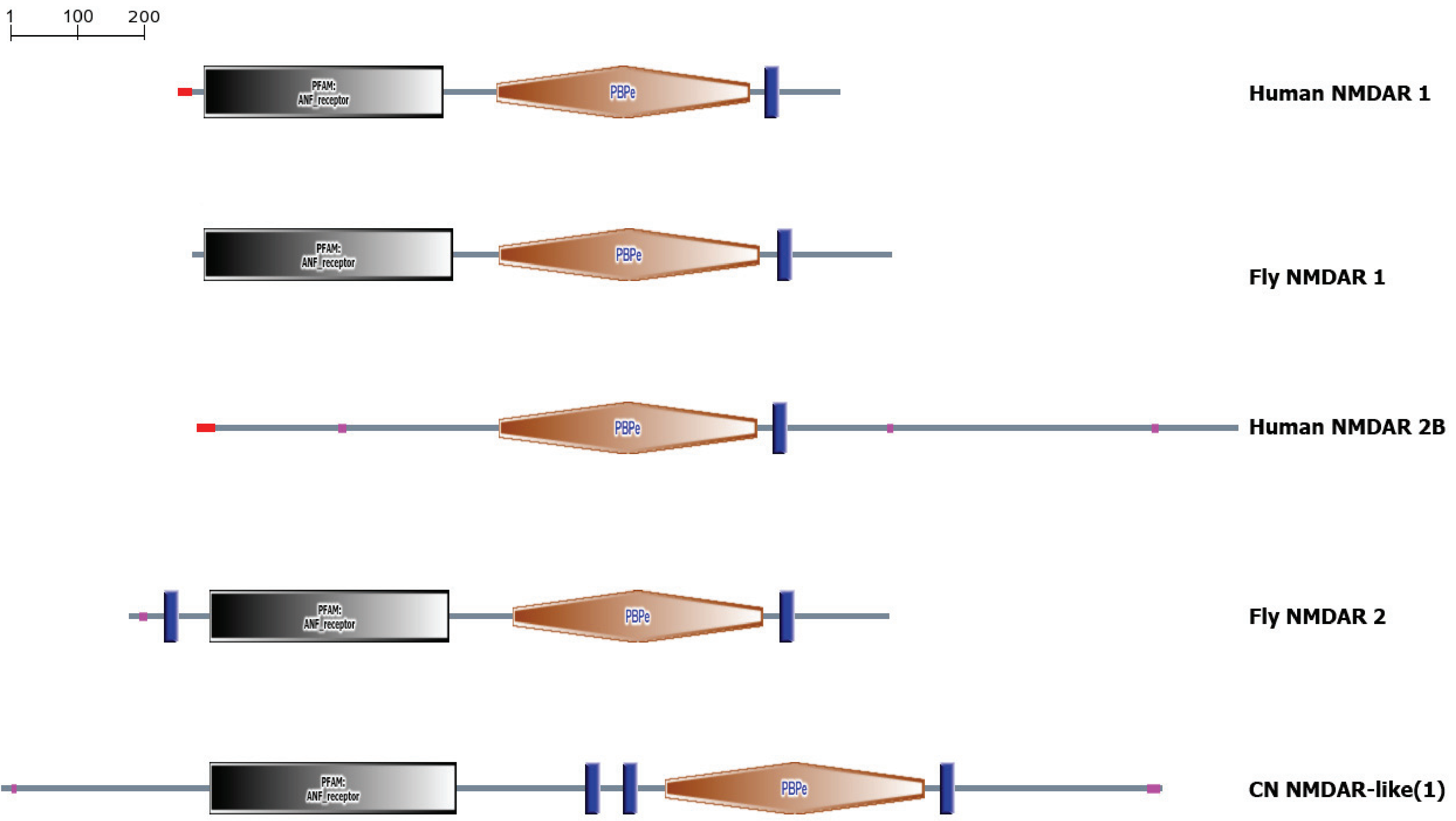

Figure S2.19.a. Domain architecture display of NMDA GluR family.

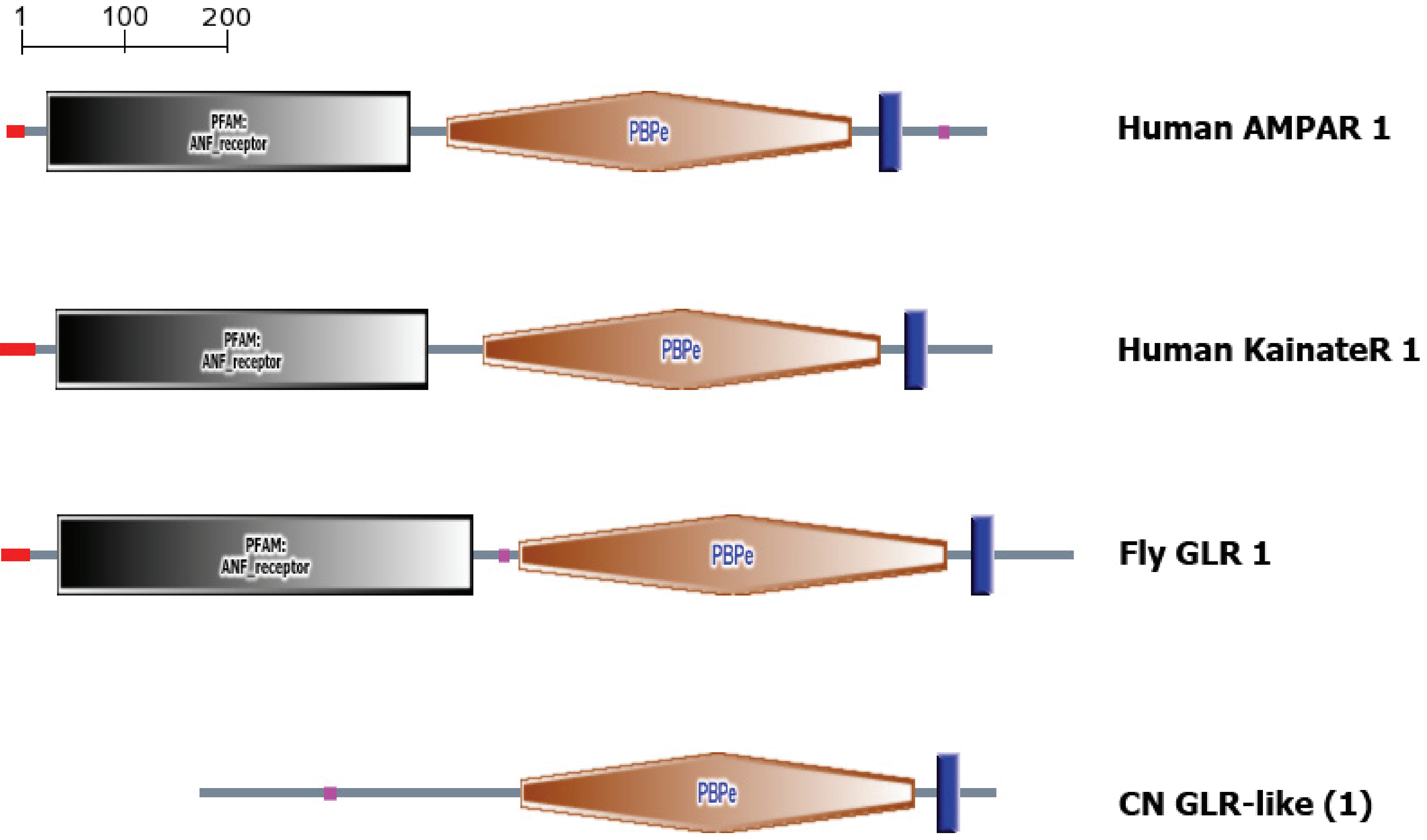

Figure S2.19.b. Domain architecture display of AMPA GluR family.

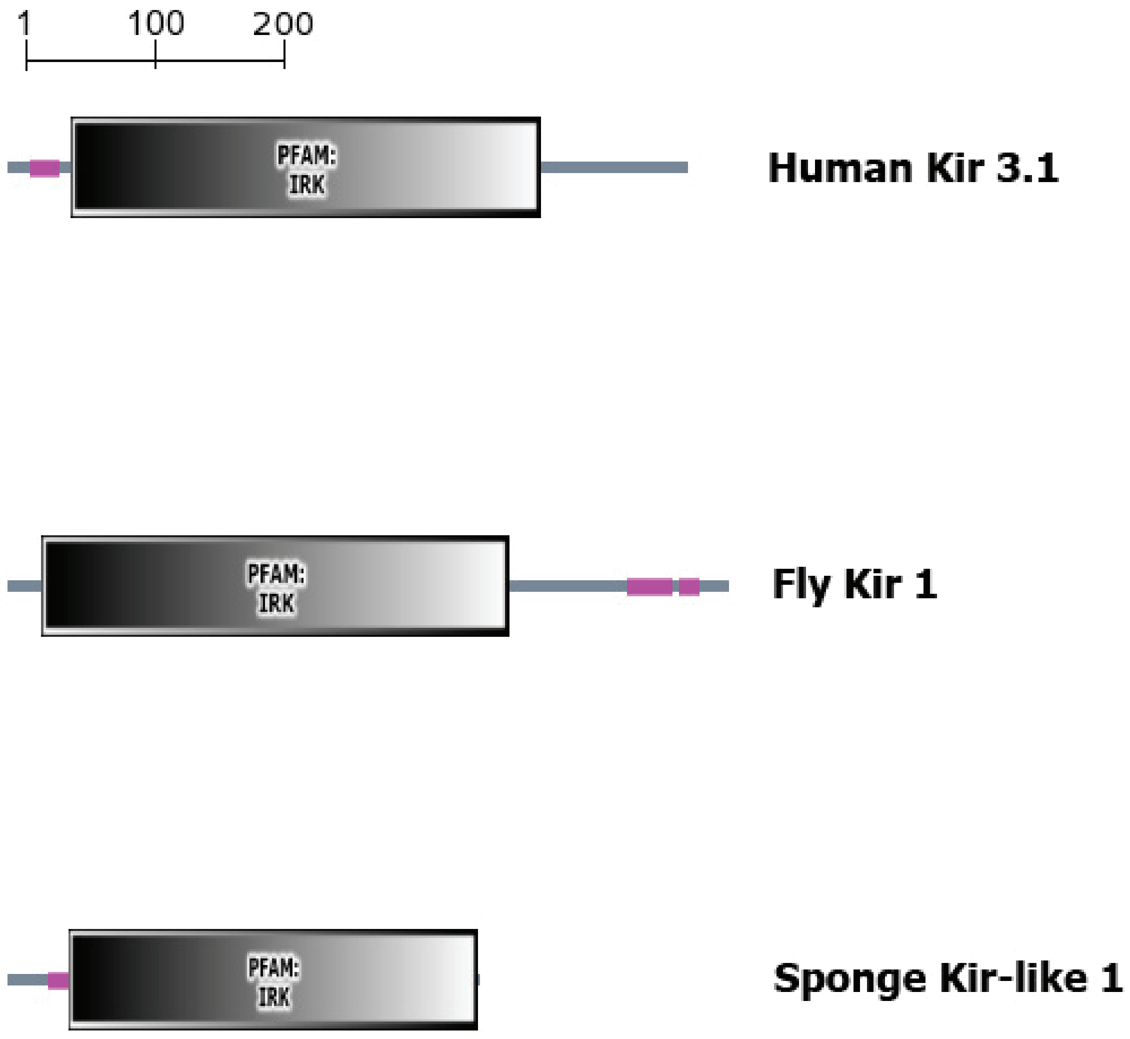

Figure S2.20. Domain architecture display of K+ Channel Kir family.

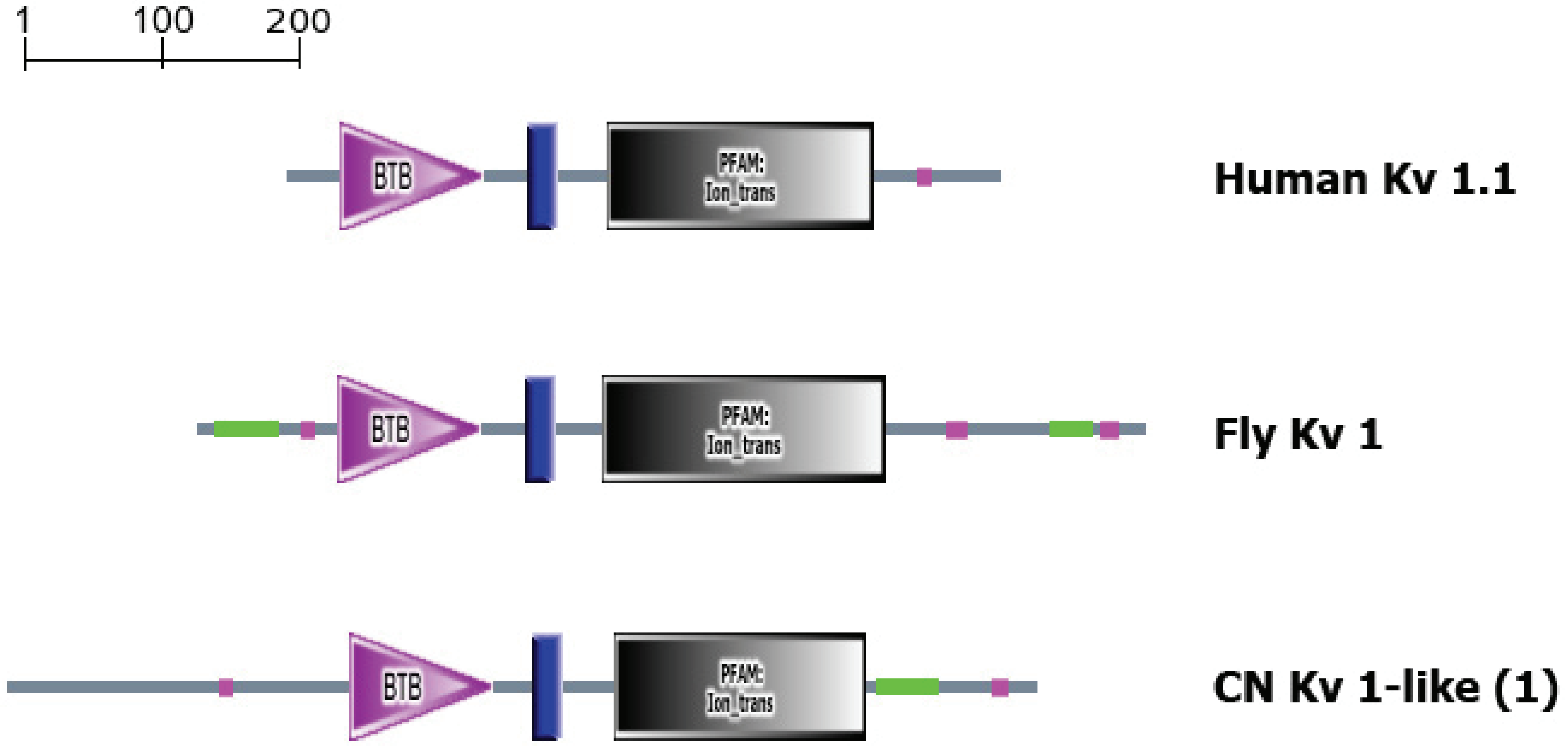

Figure S2.21. Domain architecture display of K+ Channel Shaker family.

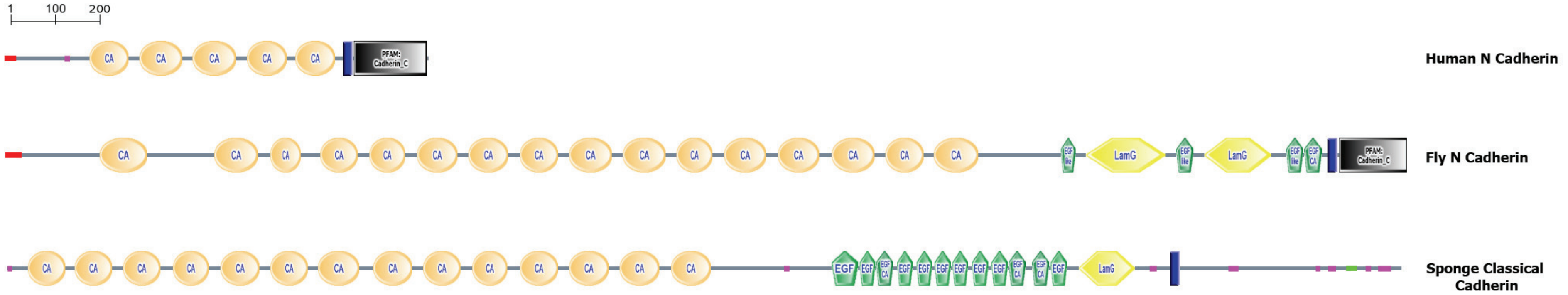

Figure S2.22. Domain architecture display of Classical Cadherin family.

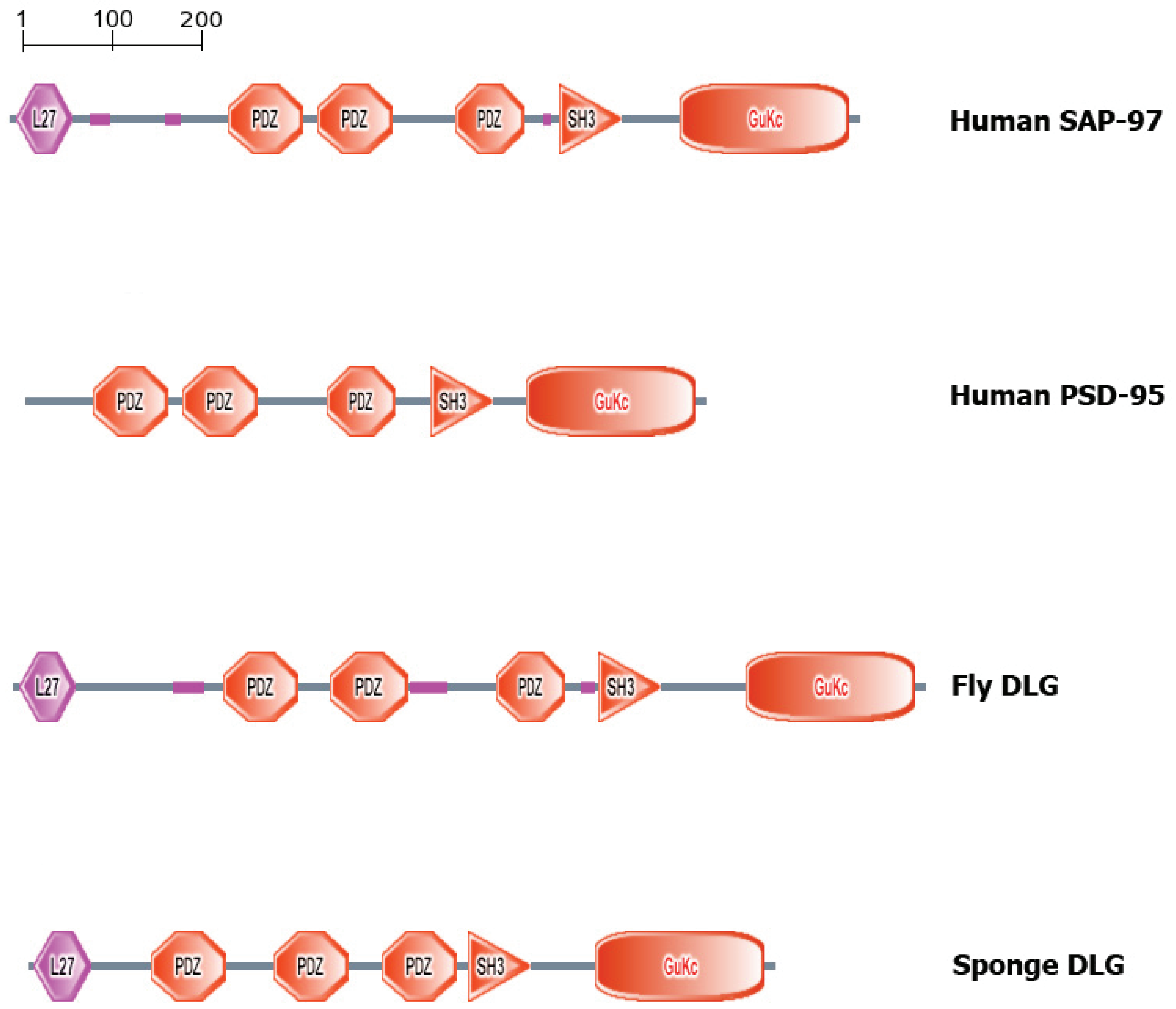

Figure S2.23.a. Domain architecture display of DLG family.

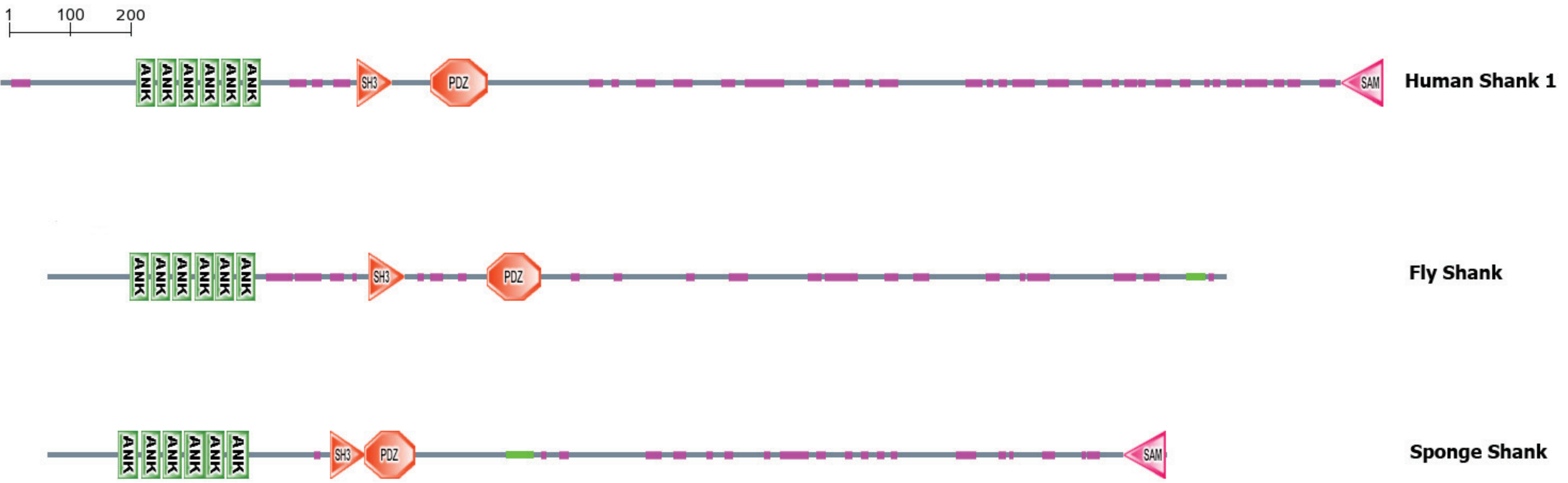

Figure S2.23.b. Domain architecture display of Shank family.

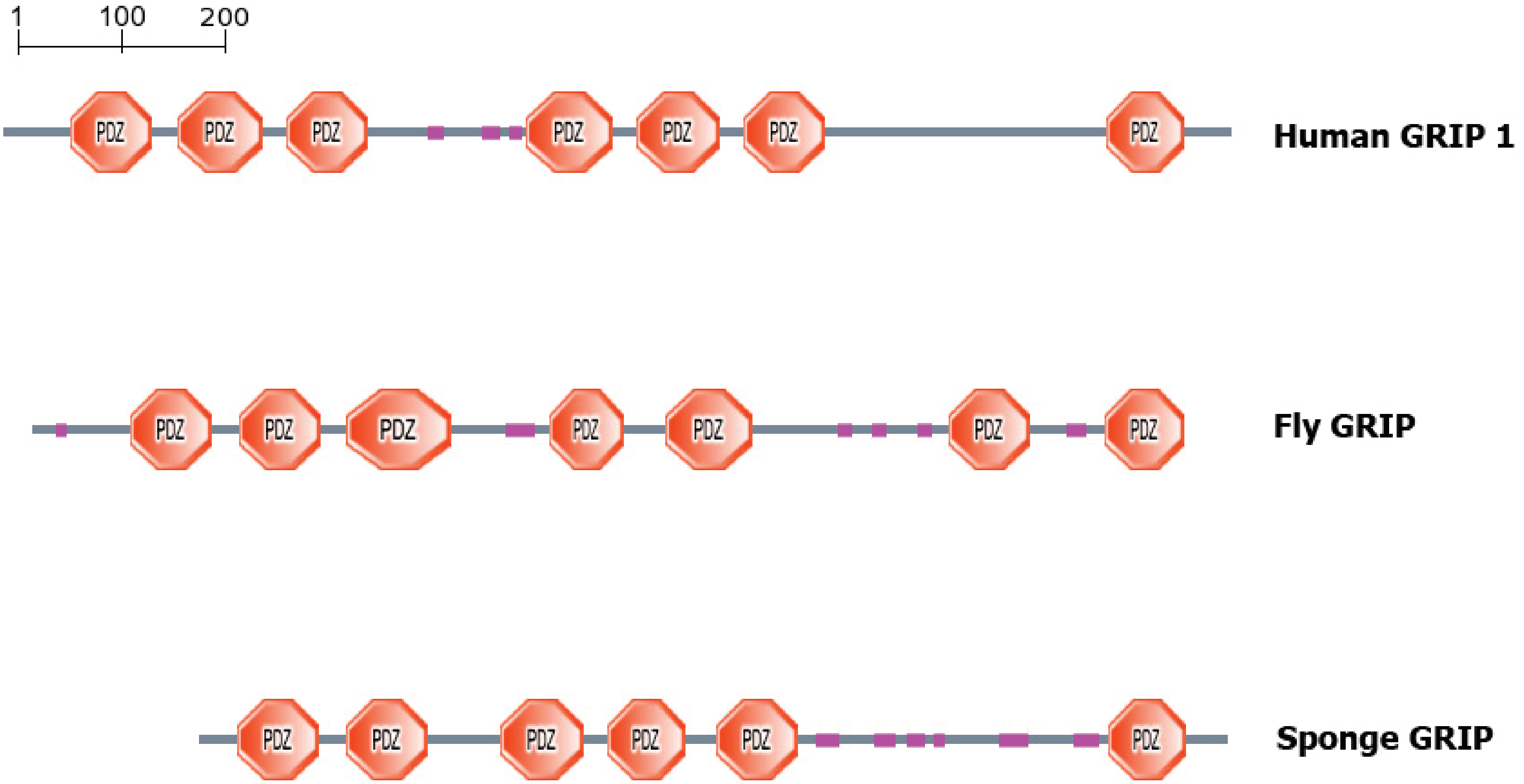

Figure S2.23.c. Domain architecture display of GRIP family.

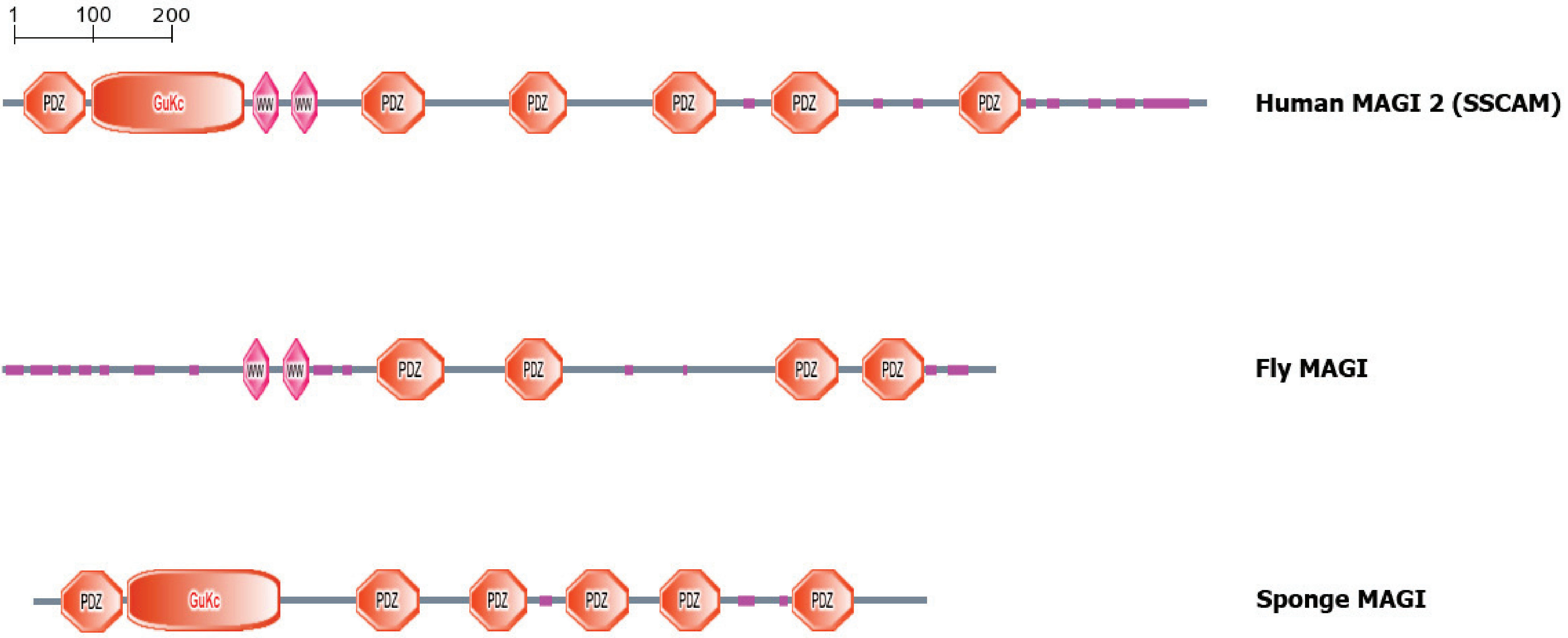

Figure S2.23.d. Domain architecture display of MAGI family.

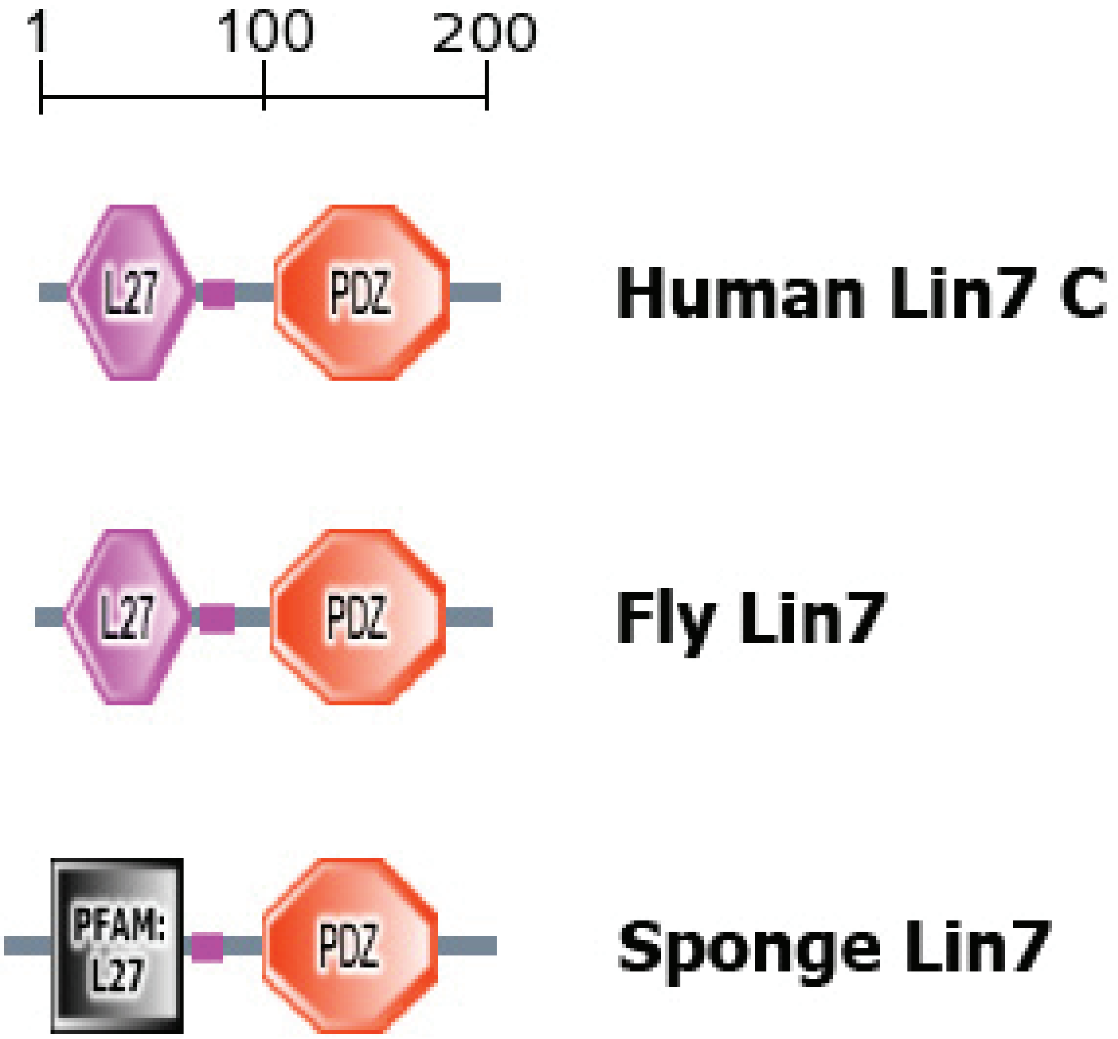

Figure S.23.e. Domain architecture display of LIN-7 family.

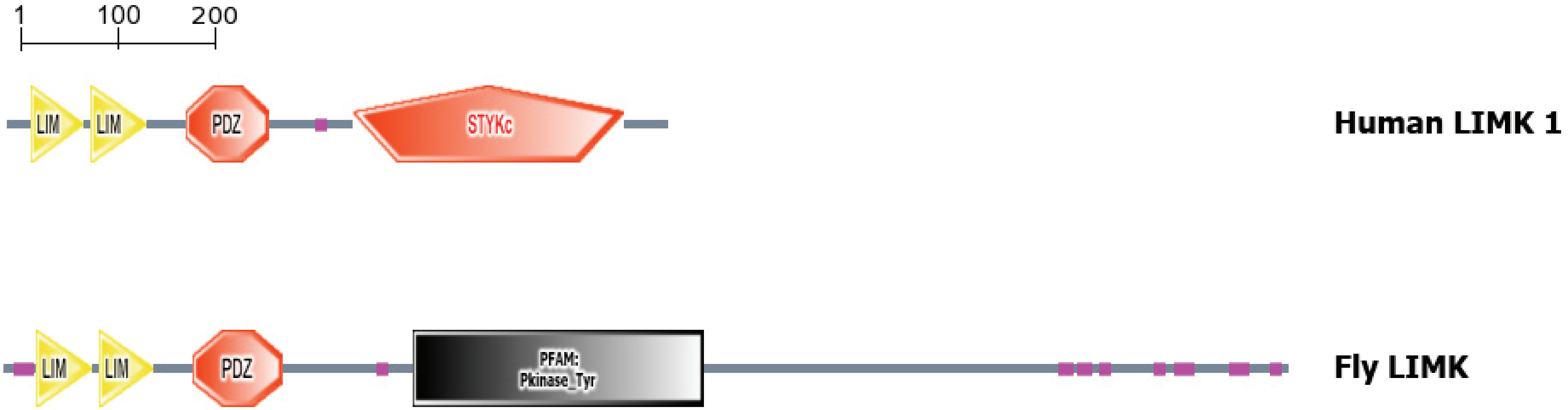

Figure S2.23.f. Domain architecture display of LIMK family.

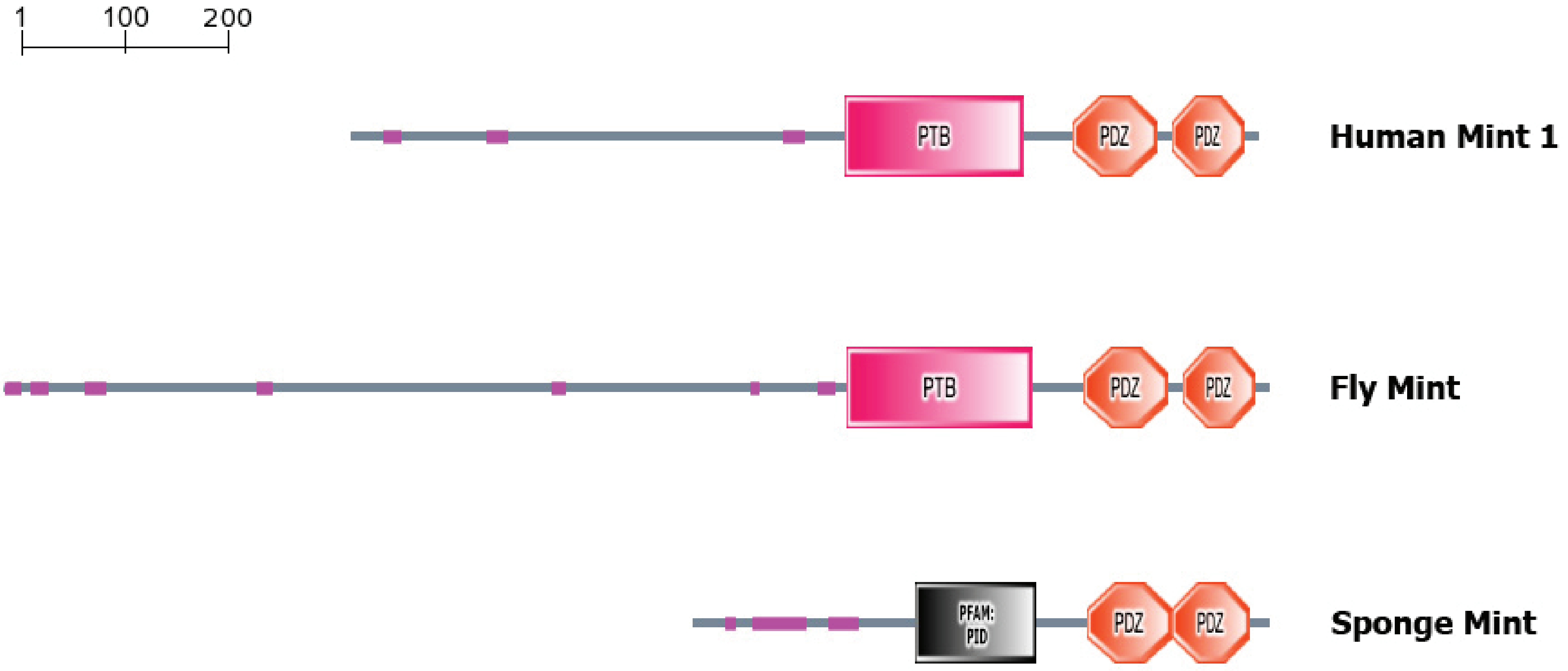

Figure S2.23.g. Domain architecture display of Mint family.

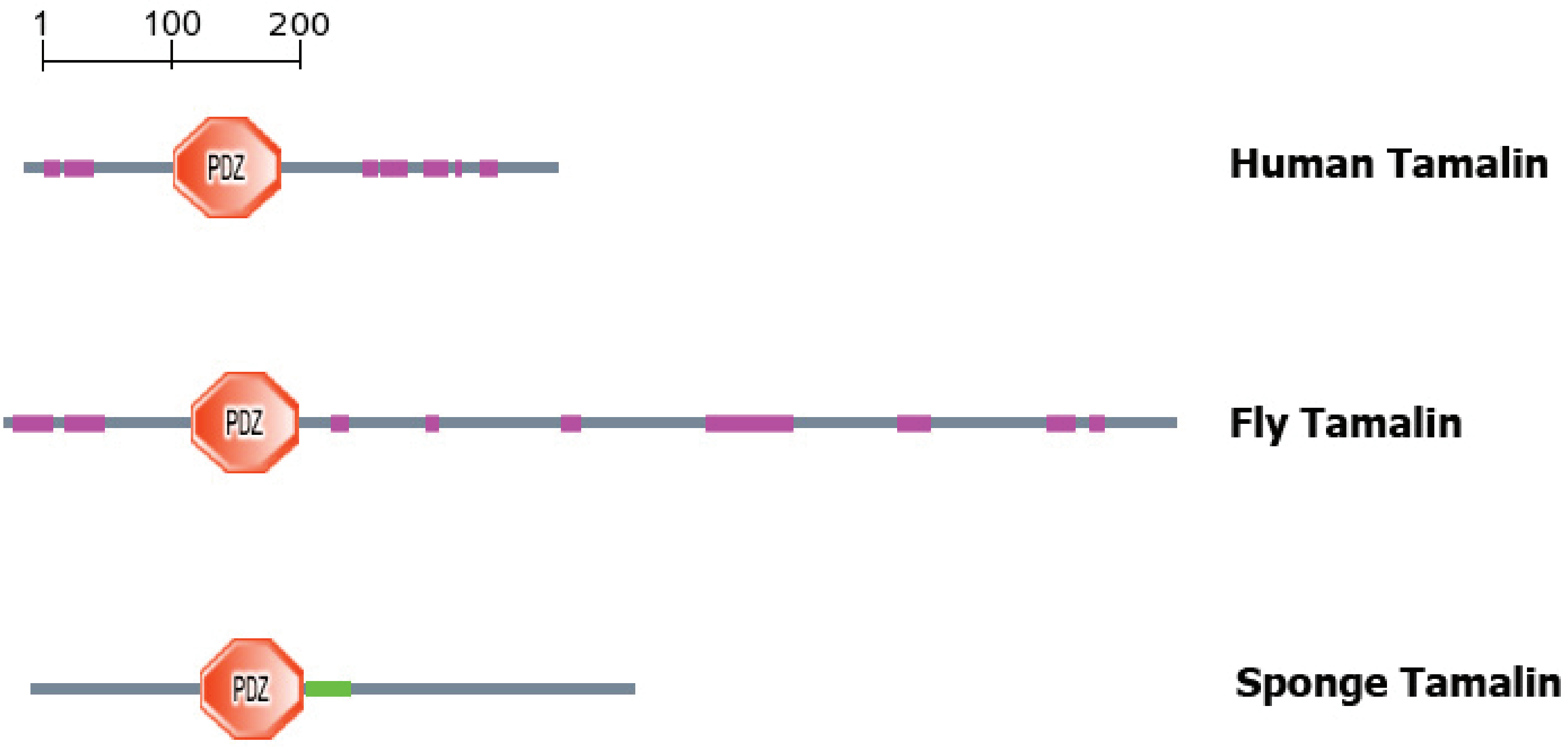

Figure S2.23.h. Domain architecture display of Tamalin family.

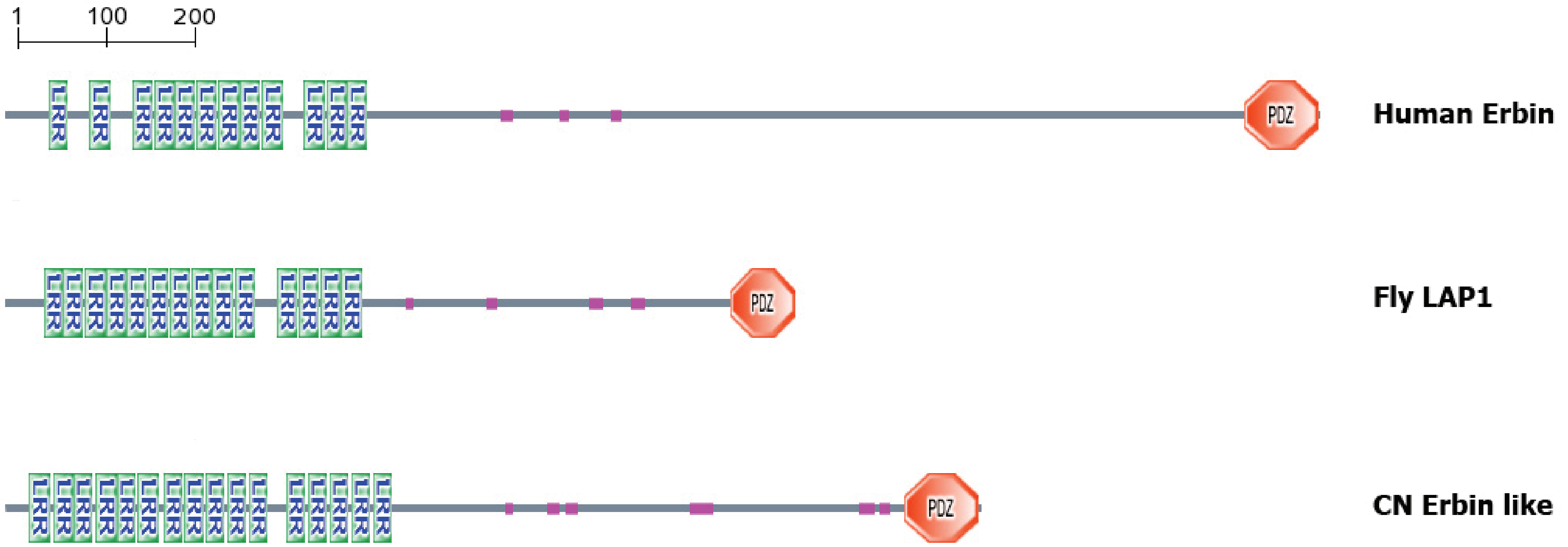

Figure S2.23.i. Domain architecture display of Erbin family.

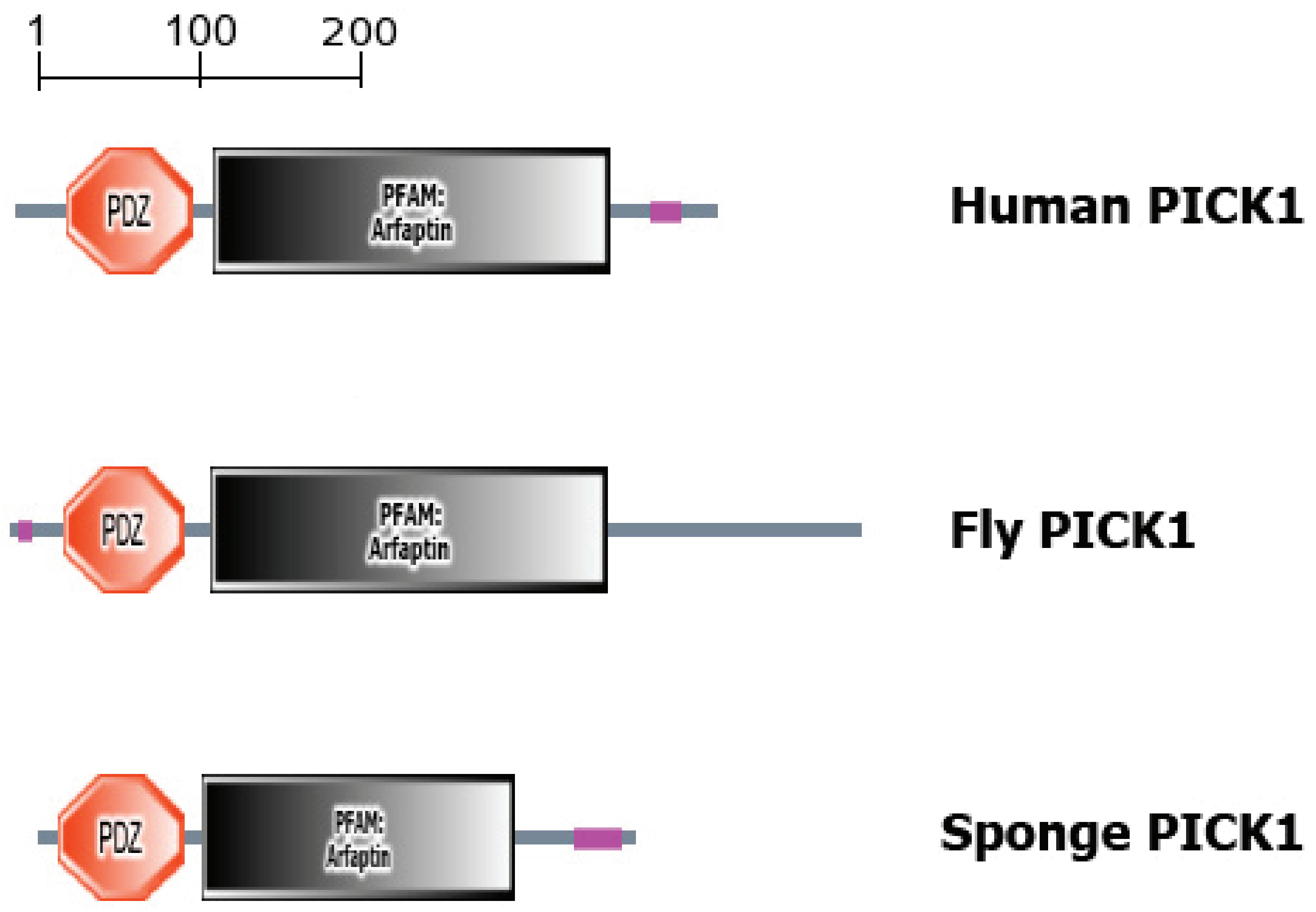

Figure S.23.j. Domain architecture display of PICK1 family.
