# Supplementary material for: A Post-Synaptic Scaffold at the Origin of the Animal Kingdom
Source: PLoS One. 2007 Jun 6;2(6):e506. doi: 10.1371/journal.pone.0000506 (PMC1876816; doi:10.1371/journal.pone.0000506)
Supplement: Figure S3 — DLG PDZ-3 sequence alignment through 8 species. Alignment of PDZ3 sequences used for homology modeling. Conserved residues are colored according to the scheme shown. Residues that can interact with CRIPT are noted by a gray line. (0.10 MB PDF) [file pone.0000506.s003.pdf]

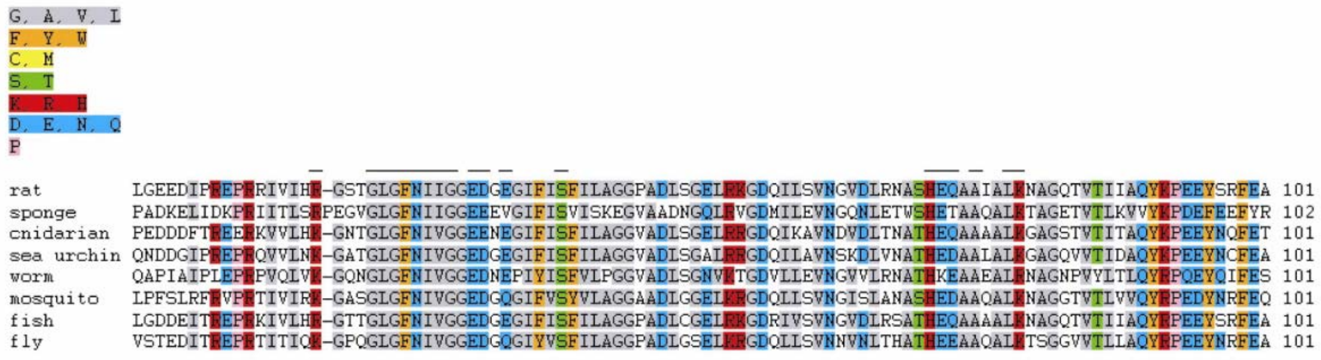
G, A, V, L
F, Y, W
C, M
S, T
K, R, H
D, E, N, Q
P
rat LGEEDIPREPRRIVIHR-GSTGLGFNIIGGEDGEGIFISFILAGGPADLSGELRKGDQIILSVNGVDLRNASHEQAAIALKNAGQTVTIIAQYKPEEYSRFEA 101
sponge PADKELIDKPRIITLSRPEGVGLGFNIIGGEEEVGIFISVISKEGVAADNGQLRVGDMILEVNGQNLETWSHETAAQALKTAGETVTLKVVYKPDEFEEFYR 102
cnidarian PEDDDFTREERKVVLHK-GNTGLGFNIVGGEENEGIFISFILAGGVADLSGELRRGDQIKAVNDVDLTNATHEQAAAALKGAGSTVTITAQYKPEEYNQFET 101
sea urchin QNDDGIPREPRQVVLNK-GATGLGFNIVGGEDGEGIFISFILAGGVADLSGALRRGDQIILAVNSKDLVNATHEDAALALKGAGQVVTIDAQYKPEEYNCFEA 101
worm QAPIAIPLEPRPVQLVK-GQNGLGFNIVGGEDNEPIYISFVLPGGVADLSGNVKTGDVLLEVNGVVLRNATHKEAAEALRNAGNPVYLTLQYRPQEYQIFES 101
mosquito LPFSLRFRVPRTIVIRK-GASGLGFNIVGGEDGQGIFVSYVLAGGAADLGGELKRGDQLLSVNGISLANASHEDAAQALKNAGGTVTLVVQYRPEDYNRFEQ 101
fish LGDDEITREPRKIVLHR-GTTGLGFNIVGGEDGEGIFISFILAGGPADLCGELRKGDRIVSVNGVDLRSATHEQAAAALKNAGQTVTIIAQYRPEEYSRFEA 101
fly VSTEDITREPRTITIQK-GPQGLGFNIVGGEDGQGIYVSFILAGGPADLGSELKRGDQLLSVNNVNLTHATHEEAAQALKTSGGVVTLLAQYRPEEYNRFEA 101
