# Supplementary figures and images for: A Post-Synaptic Scaffold at the Origin of the Animal Kingdom
Source: PLoS One. 2007 Jun 6;2(6):e506. doi: 10.1371/journal.pone.0000506 (PMC1876816; doi:10.1371/journal.pone.0000506)

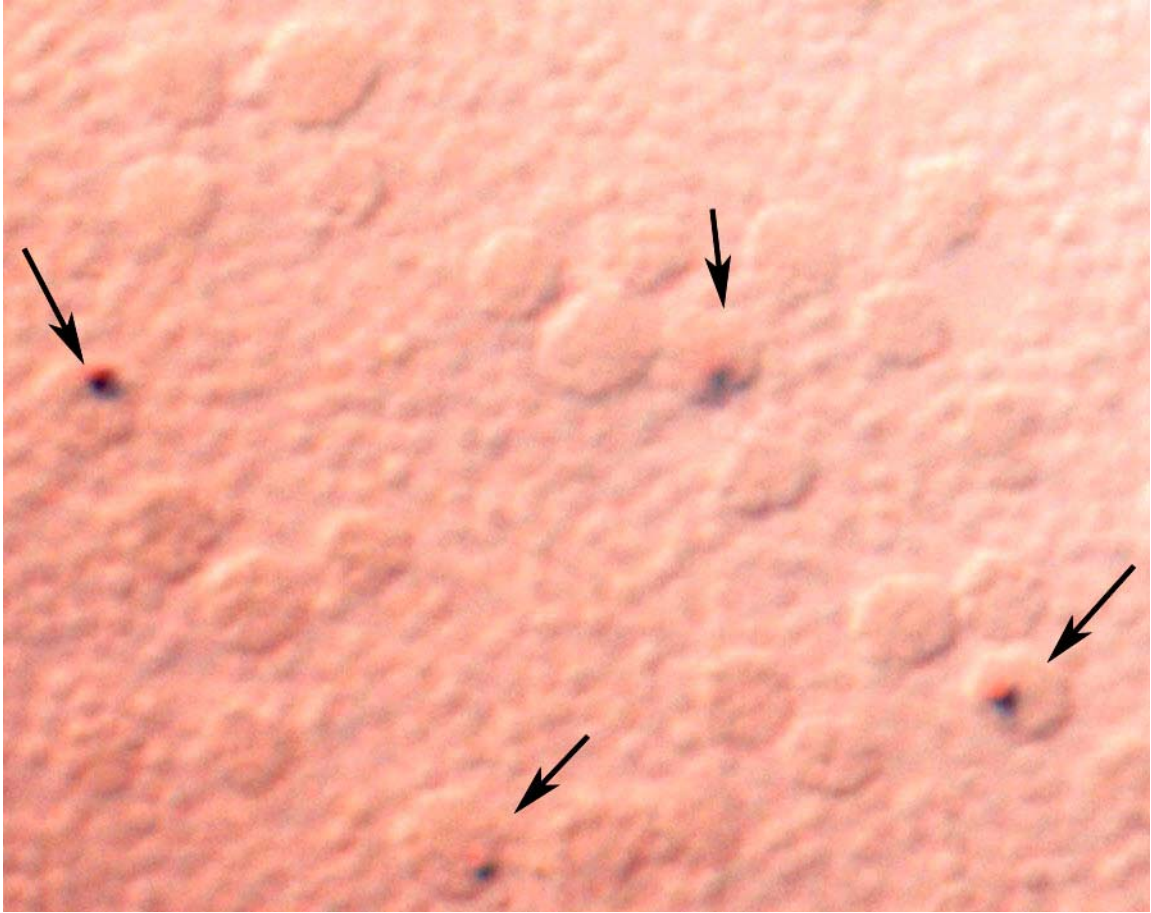

Supplement: Figure S5 — A surface view of an Amphimedon larva showing HOMER is expressed in a limited number of flask cells (arrows) by whole mount in situ hybridization. The large cells are the flask cells interspersed among the more numerous columnar epithelial cells. Transcripts are not detected in the remaining negatively stained flask cells in this field of view. (0.07 MB PDF) [file pone.0000506.s005.pdf]

**blastula**

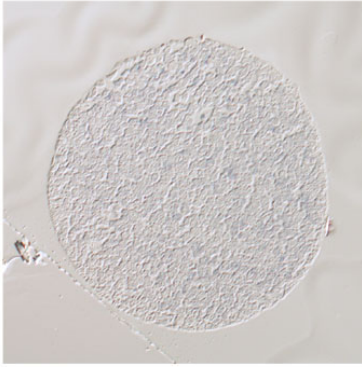

**very early gastrula**

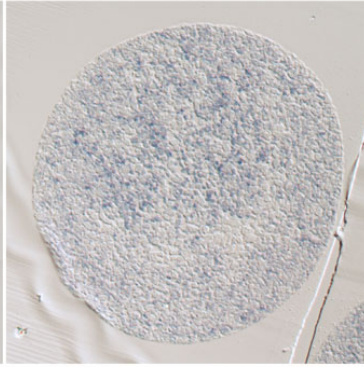

**early gastrula**

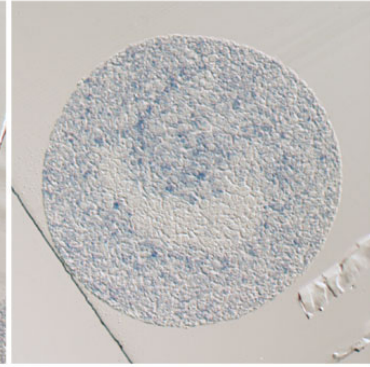

**gastrula**

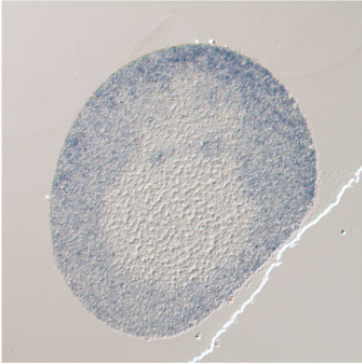

**spot**

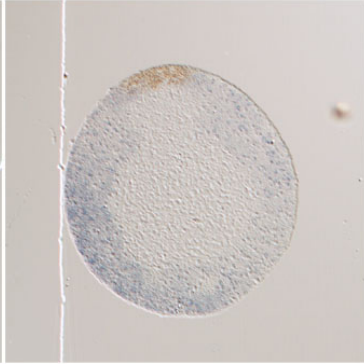

**ring**

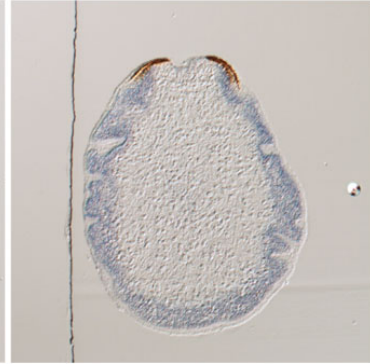

Supplement: Figure S6 — Developmental expression of dlg during Amphimedon embryogenesis. All panels are sections of in situ hybridized embryos; posterior pole is to the top. In the blastula, a small number of small cells express dlg. During the gastrulation-like stage, dlg-expressing cells sort to the outer layer; no expression is detected in the inner cell mass. At the later spot and ring stages, prior to hatching, cells expressing dlg are restricted to the outer epithelial-like layer. After hatching (Figure 3), flask cells in this layer express dlg at a higher level than the surrounding columnar epithelium. (1.01 MB PDF) [file pone.0000506.s006.pdf]
