# Supplementary material for: A Post-Synaptic Scaffold at the Origin of the Animal Kingdom
Source: PLoS One. 2007 Jun 6;2(6):e506. doi: 10.1371/journal.pone.0000506 (PMC1876816; doi:10.1371/journal.pone.0000506)
Supplement: Table S1 — Protein accession numbers for sequences used in phylogenetic analyses. Each table contains the accession numbers for the sequences used in the corresponding tree from Figure S1. PDZ and iGluR genes/domains that are shown in table S1.19 and S1.23 in grey letters are not included in phylogenetic analyses. In table S1.23, PDZ domain amino-acid locations on their corresponding proteins are shown in parenthesis after abbreviation and all gi numbers correspond to their proteins. Abbreviations used are: Sponge, Amphimedon queenslandica; CN, Nematostella vectensis; Human, Homo sapiens; Fly, Drosophila melanogaster; Yeast, Saccharomyces cerevisiae; Dicty, Dictyostelium discoideum; At, Arabidopsis thaliana; Os, Oryza sativa. (0.03 MB PDF) [file pone.0000506.s007.pdf]

Table S1.1. NOS family.

Abbrevietion	gi	Swissprot Locus	Description
At NADPH-like	7269302	N.A.	NADPH-ferrihemoprotein reductase ATR1
Os NADPH-like	50942815	N.A.	putative cytochrome P450 reductase
Dicty NADPH-like	66799961	N.A.	sulfite reductase (NADPH)
Yeast NADPH-like	730126	NCPR_YEAST	NADPH--cytochrome P450 reductase (CPR) (P450R)
Sponge NOS-like	N.A.	N.A.	**N.A.**
Sponge NADPH-like	N.A.	N.A.	N.A.
Fly NOS	26006989	NOS_DROME	CG6713 Nitric-oxide synthase (dNOS)
Fly NADPH	12643739	NCPR_DROME	CG11567 NADPH--cytochrome P450 reductase (CPR) (P450R)
Human nNOS	1709333	NOS1_HUMAN	Nitric-oxide synthase, brain (NOS type I) (Neuronal NOS) (N-NOS) (nNOS)
Human eNOS	266648	NOS3_HUMAN	Nitric-oxide synthase, endothelial (Endothelial NOS) (eNOS)
Human iNOS	1352513	NOS2A_HUMAN	Nitric oxide synthase, inducible (Inducible NOS) (iNOS)
Human NADPH	2851393	NCPR_HUMAN	NADPH--cytochrome P450 reductase (CPR) (P450R)

Table S1.2. PMCA family.

Abbrevietion	gi	Swissprot Locus	Description
At PMCA-like	12643246	ACA8_ARATH	Putative calcium-transporting ATPase 11, plasma membrane-type
Os PMCA-like	75322378	ACA6_ORYSA	Probable calcium-transporting ATPase 6, plasma membrane-type
Dicty PMCA-like	1703456	ATC1_DICDI	Probable calcium-transporting ATPase PAT1
Yeast PMCA-like	728904	ATC2_YEAST	Calcium-transporting ATPase 2 (Vacuolar Ca(2+)-ATPase)
Sponge PMCA-like	N.A.	N.A.	**N.A.**
Fly PMCA	62473111	N.A.	CG2165
Human PMCA1	14286104	AT2B1_HUMAN	Plasma membrane calcium-transporting ATPase 1 (PMCA1)
Human PMCA2	14286115	AT2B2_HUMAN	Plasma membrane calcium-transporting ATPase 2 (PMCA2)
Human PMCA3	14286116	AT2B3_HUMAN	Plasma membrane calcium-transporting ATPase 3 (PMCA3)
Human PMCA4	14286105	AT2B4_HUMAN	Plasma membrane calcium-transporting ATPase 4 (PMCA4)

Table S1.3. CaMKII and CASK families.

Abbrevietion	gi	Swissprot Locus	Description
At CaMK-like	12322336	N.A.	calcium-dependent protein kinase
Os CaMK-like	50355719	N.A.	putative calcium-dependent protein kinase
Dicty CaMK-like	1730055	MYLK_DICDI	Myosin light chain kinase (MLCK)
Yeast CaMK-like	2507190	KCC2_YEAST	Calcium/calmodulin-dependent protein kinase II
Sponge CaMKI-like	N.A.	N.A.	N.A.
Sponge CaMKII-like	N.A.	N.A.	**N.A.**
CN CASK-like	N.A.	N.A.	**N.A.**
Fly CaMKII	46576378	KCC2A_DROME	CG18069 Calcium/calmodulin-dependent protein kinase type II alpha chain
Fly CaMKI	3893099	N.A.	CG1495 Calcium/calmodulin dependent protein kinase I
Fly CaMGUK	34223738	CAKI_DROME	CG6703 Calcium/calmodulin-dependent protein kinase (CaMGUK)
Human CaMKII alpha	20177970	KCC2A_HUMAN	Calcium/calmodulin-dependent protein kinase type II alpha chain
Human CaMKII beta	12643413	KCC2B_HUMAN	Calcium/calmodulin-dependent protein kinase type II beta chain
Human CaMKII delta	12643414	KCC2D_HUMAN	Calcium/calmodulin-dependent protein kinase type II delta chain
Human CaMKII gamma	62512173	KCC2G_HUMAN	Calcium/calmodulin-dependent protein kinase type II gamma chain
Human CaMKI alpha	3122301	KCC1A_HUMAN	Calcium/calmodulin-dependent protein kinase type 1 (CaM-KI) (CaM kinase I alpha)
Human CaMKI beta	67466915	KCC1B_HUMAN	Calcium/calmodulin-dependent protein kinase type 1B(CaM kinase I beta)
Human CaMKI delta	56404610	KCC1D_HUMAN	Calcium/calmodulin-dependent protein kinase type 1D (CaM kinase I delta)
Human CaMKI gamma	73620970	KCC1G_HUMAN	Calcium/calmodulin-dependent protein kinase type 1G (CaM kinase I gamma)
Human CASK	27735175	CSKP_HUMAN	Peripheral plasma membrane protein CASK (hCASK)

Table S1.4. IP3R family.

Abbrevietion	gi	Swissprot Locus	Description
Sponge IP3R-like	N.A.	N.A.	**N.A.**
Sponge RYR-like	N.A.	N.A.	N.A.
Fly IP3R	44888998	ITPR_DROME	CG1063 Inositol 1,4,5-trisphosphate receptor (InsP3 receptor) (InsP3R)
Fly RYR	456984	RY44_DROME	CG10844 ryanodine receptor, calcium release channel
Human IP3R1	17366467	ITPR1_HUMAN	Inositol 1,4,5-trisphosphate receptor type 1 (Type 1 inositol 1,4,5-trisphosphate receptor)
Human IP3R2	2833252	ITPR2_HUMAN	Inositol 1,4,5-trisphosphate receptor type 2 (Type 2 inositol 1,4,5-trisphosphate receptor)
Human IP3R3	17366458	ITPR3_HUMAN	Inositol 1,4,5-trisphosphate receptor type 3 (Type 3 inositol 1,4,5-trisphosphate receptor)
Human RYR1	19857096	RYR1_HUMAN	Ryanodine receptor 1 (Skeletal muscle-type ryanodine receptor) (RyR1) (RYR-1)
Human RYR2	17380312	RYR2_HUMAN	Ryanodine receptor 2 (Cardiac muscle-type ryanodine receptor) (RyR2) (RYR-2)
Human RYR3	18202506	RYR3_HUMAN	Ryanodine receptor 3 (Brain-type ryanodine receptor) (RyR3) (RYR-3)

Table S1.5. Delta Catenin and Beta Catenin families.

Abbrevietion	gi	Swissprot Locus	Description
At ARMC6-like	111074180	N.A.	At4g33945
Os ARMC6-like	50913021	N.A.	armadillo/beta-catenin repeat protein-like
Dicty ARMC6-like	10444518	N.A.	Aardvark
Sponge p120/Delta Catenin-like	N.A.	N.A.	**N.A.**
Sponge Beta Catenin-like	N.A.	N.A.	**N.A.**
Fly Beta Catenin	114162	ARM_DROME	CG11579 Armadillo segment polarity protein
Fly Delta Catenin	6959880	N.A.	CG17484 Adherens junction protein p120
Fly ARMC6	19922518	N.A.	CG5721
Human p120 Catenin	14916543	CTND1_HUMAN	Catenin delta-1 (p120 catenin) (p120(ctn)) (Cadherin-associated Src substrate)
Human Delta Catenin	84028193	CTND2_HUMAN	Catenin delta-2 (Delta-catenin) (Neural plakophilin-related ARM-repeat protein)
Human Plakophilin1	20138951	PKP1_HUMAN	Plakophilin-1 (Band-6 protein) (B6P)
Human Plakophilin2	20139105	PKP2_HUMAN	Plakophilin-2
Human Plakophilin3	20139301	PKP3_HUMAN	Plakophilin-3
Human p0071	20139104	PKP4_HUMAN	Plakophilin-4 (p0071)
Human ARVC	12229553	ARVC_HUMAN	Armadillo repeat protein deleted in velo-cardio-facial syndrome
Human Beta Catenin	461854	CTNB1_HUMAN	Beta-catenin

Human PLAK	130257	PLAK_HUMAN	Junction plakoglobin (Desmoplakin-3) (Desmoplakin III)
Human ARMC6	109940304	ARMC6_HUMAN	Armadillo repeat-containing protein 6

Table S1.6. GKAP family.

Abbrevietion	gi	Swissprot Locus	Description
Sponge GKAP/DLG7	N.A.	N.A.	**N.A.**
CN GKAP-like	N.A.	N.A.	N.A.
CN DLG7-like	N.A.	N.A.	N.A.
Fly vulcan	24585883	N.A.	CG8390 vulcan
Fly mars	20129953	N.A.	CG17064 mars
Human GKAP1	18201963	DLGP1_HUMAN	Disks large-associated protein 1 (DAP-1) (hGKAP)
Human GKAP2	71153507	DLGP2_HUMAN	Disks large-associated protein 2 (DAP-2)(SAPAP2)
Human GKAP3	71153509	DLGP3_HUMAN	Disks large-associated protein 3 (DAP-3) (SAPAP3)
Human GKAP4	18203629	DLGP4_HUMAN	Disks large-associated protein 4 (DAP-4) (SAPAP4)
Human DLG7	82592583	DLG7_HUMAN	Discs large homolog 7 (Hepatoma up-regulated protein) (HURP)

Table S1.7. SynGAP family.

Abbrevietion	gi	Swissprot Locus	Description
Dicty GAP-like	66816789	N.A.	NGAP-like protein
Yeast GAP-like	543907	BUD2_YEAST	Inhibitory regulator protein BUD2/CLA2
Sponge GAP1	N.A.	N.A.	N.A.
Sponge SynGAP-like (GAP2)	N.A.	N.A.	**N.A.**
Sponge GAP3	N.A.	N.A.	N.A.
Fly GAP1	68067650	GAP1_DROME	CG6721 GTPase-activating protein
Fly SynGAP-like (GAP2)	33112300	GAP2_DROME	CG32560 Probable Ras GTPase-activating protein
Fly GAP3	4107166	N.A.	CG9209 vacuolar peduncle
Human SynGAP	34098679	SYGP1_HUMAN	Ras GTPase-activating protein SynGAP
Human NGAP	13959419	NGAP_HUMAN	Ras GTPase-activating protein nGAP
Human RasP21-1	4506431	N.A.	RAS p21 protein activator 1 isoform 1
Human RasP21-2	12545408	N.A.	RAS p21 protein activator 2
Human RasP21-3	38201692	N.A.	RAS p21 protein activator 3
Human RasP21-4	31745138	N.A.	RAS p21 protein activator 4

Table S1.8. Cript family.

Abbrevietion	gi	Swissprot Locus	Description
At Cript-like	15220607	N.A.	unknown protein
Os Cript-like	50941887	N.A.	putative postsynaptic protein CRIPT
Sponge Cript-like	N.A.	N.A.	**N.A.**
Fly Cript	7297565	N.A.	CG4537
Human Cript	7661798	N.A.	postsynaptic protein CRIPT

Table S1.9. Homer family.

Abbrevietion	gi	Swissprot Locus	Description
Dicty VASP-like	66804927	N.A.	vasodilator-stimulated phosphoprotein
Sponge Homer-like	N.A.	N.A.	**N.A.**
Fly Homer	3834623	N.A.	CG11324 homer
Fly Enabled	24655476	N.A.	CG15112 enabled
Human Homer1	38604765	HOME1_HUMAN	Homer protein homolog 1
Human Homer2	38605067	HOME2_HUMAN	Homer protein homolog 2
Human Homer3	38605068	HOME3_HUMAN	Homer protein homolog 3
Human Enabled	48428086	ENAH_HUMAN	Protein enabled homolog
Human VASP	1718079	VASP_HUMAN	Vasodilator-stimulated phosphoprotein

Table S1.10. SPAR family.

Abbrevietion	gi	Swissprot Locus	Description
Dicty RapGAP-like	66802848	N.A.	hypothetical protein DDB0183944
Sponge SPAR-like	N.A.	N.A.	**N.A.**
Sponge Rap1GAP-like	N.A.	N.A.	N.A.
Fly Rap1GAP	2655096	N.A.	CG33529 Rapgap1
Human SI1L1 (SPAR)	50401319	SI1L1_HUMAN	Signal-induced proliferation-associated 1-like protein 1
Human SI1L2	85681894	SI1L2_HUMAN	Signal-induced proliferation-associated 1-like protein 2
Human SI1L3	50401326	SI1L3_HUMAN	Signal-induced proliferation-associated 1-like protein 3
Human Rap1GAP1	1350590	RGP2_HUMAN	Rap1 GTPase-activating protein 1 (Rap1GAP)
Human Rap1GAP2	51869679	N.A.	Rap1 GTPase activating protein 2a

Table S1.11. Cortactin family.

Abbrevietion	gi	Swissprot Locus	Description
Dicty SH3_Cortactin-like	28828733	N.A.	hypothetical protein
Yeast ABP1	113000	ABP1_YEAST	Actin-binding protein
Sponge Cortactin-like	N.A.	N.A.	**N.A.**
Fly Cortactin	24648611	N.A.	CG3637 Cortactin
Human Cortactin	2498954	SRC8_HUMAN	Src substrate cortactin (Amplaxin) (Oncogene EMS1)
Human HCLS1	123557	HCLS1_HUMAN	Hematopoietic lineage cell-specific protein

Table S1.12. Metabotropic GluR and GABAB Receptor families.

Abbrevietion	gi	Swissprot Locus	Description
At GLR33	41017227	GLR33_ARATH	Glutamate receptor 3.3 precursor (Ligand-gated ion channel 3.3)
Os GLR	50904793	N.A.	putative glutamate receptor
Dicty GABABR-like	60463477	N.A.	G-protein-coupled receptor (GPCR) family protein
Sponge mGluR-like(1)	N.A.	N.A.	**N.A.**
Sponge mGluR-like(2)	N.A.	N.A.	**N.A.**

Sponge mGluR-like(3)	N.A.	N.A.	**N.A.**
Sponge mGluR-like(4)	N.A.	N.A.	**N.A.**
Sponge mGluR-like(5)	N.A.	N.A.	**N.A.**
Sponge mGluR-like(6)	N.A.	N.A.	**N.A.**
Sponge mGluR-like(7)	N.A.	N.A.	**N.A.**
Sponge mGluR-like(8)	N.A.	N.A.	**N.A.**
Sponge GABABR-like	N.A.	N.A.	**N.A.**
Fly GABABR 1	13160942	N.A.	CG15274 metabotropic GABA-B receptor subtype 1
Fly GABABR 2	13160945	N.A.	CG6706 metabotropic GABA-B receptor subtype 2
Fly GABABR 3	24580689	N.A.	CG3022 metabotropic GABA-B receptor subtype 3
Fly mGluR	17380359	MGR_DROME	CG11144 Metabotropic glutamate receptor
Fly mGluXR	42538965	N.A.	CG30361 metabotropic X receptor
Human GABABR 1	12643873	GABR1_HUMAN	GABA-B receptor 1
Human GABABR 2	12643641	GABR2_HUMAN	GABA-B receptor 2
Human mGluR 1	62297775	MGR1_HUMAN	Metabotropic glutamate receptor 1
Human mGluR 2	76803802	MGR2_HUMAN	Metabotropic glutamate receptor 2
Human mGluR 3	76803803	MGR3_HUMAN	Metabotropic glutamate receptor 3
Human mGluR 4	2495077	MGR4_HUMAN	Metabotropic glutamate receptor 4
Human mGluR 5	1709020	MGR5_HUMAN	Metabotropic glutamate receptor 5
Human mGluR 6	3024134	MGR6_HUMAN	Metabotropic glutamate receptor 6
Human mGluR 7	2495078	MGR7_HUMAN	Metabotropic glutamate receptor 7
Human mGluR 8	12644040	MGR8_HUMAN	Metabotropic glutamate receptor 8
Human CASR	1168781	CASR_HUMAN	Extracellular calcium-sensing receptor
Human GPC6A	74745292	GPC6A_HUMAN	G-protein coupled receptor family C group 6 member A
Human TS1R 1	57013075	TS1R1_HUMAN	Taste receptor type 1 member 1
Human TS1R 2	57013086	TS1R2_HUMAN	Taste receptor type 1 member 2
Human TS1R 3	62299063	TS1R3_HUMAN	Taste receptor type 1 member 3

Table S1.13. PKC family.

Abbrevietion	gi	Swissprot Locus	Description
At PKC-like	21431798	KPK19_ARATH	Serine/threonine-protein kinase AtPK19
Os PKC-like	108707998	N.A.	Serine/threonine-protein kinase AtPK19, putative, expressed
Dicty PKC-like	1730069	KRAC_DICDI	RAC-family serine/threonine-protein kinase homolog
Yeast PKC-like	585365	KPC1_YEAST	Protein kinase C-like 1 (PKC 1)
Sponge PKC1	N.A.	N.A.	N.A.
Sponge PKC2	N.A.	N.A.	N.A.
Sponge PKC3	N.A.	N.A.	**N.A.**
Fly PKC1	26006990	KPC1_DROME	CG6622 Protein kinase C, brain isozyme (PKC)
Fly PKC2	125543	KPC2_DROME	CG6518 Protein kinase C, eye isozyme (PKC)
Fly PKC3	125547	KPC3_DROME	CG1954 Protein kinase C (PKC) (dPKC98F)
Fly PKC4	31340528	KPC4_DROME	CG10524 Putative protein kinase C, delta type homolog
Fly aPKC	24653760	N.A.	CG10261 atypical protein kinase C
Human PKC delta	547803	KPCD_HUMAN	Protein kinase C delta type
Human PKC theta	20141582	KPCT_HUMAN	Protein kinase C theta type
Human PKC eta	1346393	KPCL_HUMAN	Protein kinase C eta type
Human PKC epsilon	400135	KPCE_HUMAN	Protein kinase C epsilon type
Human PKC beta	20141488	KPCB_HUMAN	Protein kinase C beta type
Human PKC alpha	125549	KPCA_HUMAN	Protein kinase C alpha type
Human PKC gamma	462455	KPCG_HUMAN	Protein kinase C gamma type
Human PKC zeta	68067736	KPCZ_HUMAN	Protein kinase C zeta type
Human PKC iota	1170688	KPCI_HUMAN	Protein kinase C iota type

Table S1.14. Alpha Catenin family.

Abbrevietion	gi	Swissprot Locus	Description
Dicty Vinculin-like	66808591	N.A.	putative actin binding protein
Sponge Alpha Catenin-like	N.A.	N.A.	**N.A.**
Sponge Vinculin-like	N.A.	N.A.	N.A.
Fly Alpha Catenin	21264412	CTNA_DROME	CG17947 Catenin-alpha
Fly Alpha Catulin	24762469	N.A.	CG2987 alpha-catenin-related
Fly Vinculin	50401320	VINC_DROME	CG3299 Vinculin
Human Alpha E-Catenin	461853	CTN1_HUMAN	Alpha-1 catenin (Cadherin-associated protein) (Alpha E-catenin)
Human Alpha N-Catenin	14916980	CTN2_HUMAN	Alpha-2 catenin (Alpha-catenin-related protein) (Alpha N-catenin)
Human Alpha T-Catenin	78099215	CTN3_HUMAN	Alpha-3 catenin (Alpha T-catenin) (Cadherin-associated protein)
Human Alpha Catulin	62901512	CTNL1_HUMAN	Alpha-catulin (Catenin alpha-like protein 1) (Alpha-catenin-related protein)
Human Vinculin	21903479	VINC_HUMAN	Vinculin (Metavinculin)

Table S1.15. Citron family.

Abbrevietion	gi	Swissprot Locus	Description
At kinase_171888	15219591	N.A.	kinase
At kinase_179637	15225358	N.A.	kinase
Dicty kinase_642376	66816743	N.A.	putative protein serine/threonine kinase
Sponge S_Tk1	N.A.	N.A.	N.A.
Sponge Citron-like (S_Tk2)	N.A.	N.A.	**N.A.**
Sponge S_Tk3	N.A.	N.A.	N.A.
Fly Citron	24663340	N.A.	CG10522
Fly ROCK	24642569	N.A.	CG9774 Rho-kinase
Fly MRCK	24762562	N.A.	CG4012 genghis khan
Human Citron	57015279	CTRO_HUMAN	Citron Rho-interacting kinase (CRIK)
Human ROCK1	47605999	ROCK1_HUMAN	Rho-associated protein kinase 1
Human ROCK2	47605963	ROCK2_HUMAN	Rho-associated protein kinase 2
Human MRCKA	74746874	MRCKA_HUMAN	Serine/threonine-protein kinase MRCK alpha

Human MRCKB	92090617	MRCKB_HUMAN	Serine/threonine-protein kinase MRCK beta
Human MRCKG	74762299	MRCKG_HUMAN	Serine/threonine-protein kinase MRCK gamma
Human DMPK	1706450	DMPK_HUMAN	Myotonin-protein kinase (Myotonic dystrophy protein kinase)

Table S1.16. Stargazin family.

Abbrevietion	gi	Swissprot Locus	Description
Fly VD Calcium Channel-like	71854527	N.A.	CG33670
Human VD Calcium Channel 2 (Stargazin)	6685289	CCG2_HUMAN	Voltage-dependent calcium channel gamma-2 subunit
Human VD Calcium Channel 3	6685277	CCG3_HUMAN	Voltage-dependent calcium channel gamma-3 subunit
Human VD Calcium Channel 4	10719940	CCG4_HUMAN	Voltage-dependent calcium channel gamma-4 subunit
Human VD Calcium Channel 5	20532390	CCG5_HUMAN	Voltage-dependent calcium channel gamma-5 subunit
Human VD Calcium Channel 7	51702249	CCG7_HUMAN	Voltage-dependent calcium channel gamma-7 subunit
Human VD Calcium Channel 8	20532017	CCG8_HUMAN	Voltage-dependent calcium channel gamma-8 subunit

Table S1.17. Ephrin Receptor and ErbB Receptor families.

Abbrevietion	gi	Swissprot Locus	Description
At TyrK	397645	N.A.	protein tyrosine kinase
Os TyrK	18408889	N.A.	ATP binding / kinase/ protein kinase/ protein serine/threonine kinase
Dicty TyrK	108862829	N.A.	protein kinase family protein, putative, expressed
Sponge Eph-like	N.A.	N.A.	**N.A.**
Sponge Src-like(1)	N.A.	N.A.	N.A.
Sponge Src-like(2)	N.A.	N.A.	N.A.
Sponge Abl-like	N.A.	N.A.	N.A.
Sponge Fps-like	N.A.	N.A.	N.A.
Sponge Ror-like	N.A.	N.A.	N.A.
Sponge FAK-like	N.A.	N.A.	N.A.
Sponge ErbB-like	N.A.	N.A.	**N.A.**
Fly Eph	62484409	N.A.	CG1511 Eph receptor tyrosine kinase
Fly ErbB	6175066	EGFR_DROME	CG10079 Epidermal growth factor receptor precursor (Egfr) (Gurken receptor)
Fly Src42	33112450	SRC42_DROME	CG7873 Tyrosine-protein kinase Src42A (Dsrc41)
Fly Src64	14286176	SRC64_DROME	CG7524 Tyrosine-protein kinase Src64B (Dsrc64)
Fly Abl	62512130	ABL_DROME	CG4032 Tyrosine-protein kinase Abl (Abelson protein) (D-ash)
Fly Fps	44888970	FPS_DROME	CG8874 Tyrosine-protein kinase Fps85D (dFer)
Fly Ror2	27923847	ROR2_DROME	CG4007 Tyrosine-protein kinase transmembrane receptor Ror2 precursor
Fly Ror1	27923846	ROR1_DROME	CG4926 Tyrosine-protein kinase transmembrane receptor Ror precursor
Fly Btk	25453425	BTKL_DROME	CG8049 Tyrosine-protein kinase Btk29A (Dsrc28C)
Fly FAK	24655701	N.A.	CG10023 Focal Adhesion Kinase
Human Eph B1	1706663	EPHB1_HUMAN	Ephrin type-B receptor 1 precursor (Tyrosine-protein kinase receptor EPH-2)
Human Eph B2	76803654	EPHB2_HUMAN	Ephrin type-B receptor 2 precursor (Tyrosine-protein kinase receptor EPH-3)
Human Eph B3	76803655	EPHB3_HUMAN	Ephrin type-B receptor 3 precursor (Tyrosine-protein kinase receptor HEK-2)
Human Eph B4	19860819	EPHB4_HUMAN	Ephrin type-B receptor 4 precursor (Tyrosine-protein kinase receptor HTK)
Human Eph B6	6919882	EPHB6_HUMAN	Ephrin type-B receptor 6 precursor (Tyrosine-protein kinase-defective receptor EPH-6)
Human Eph A1	47117832	EPHA1_HUMAN	Ephrin type-A receptor 1 precursor (Tyrosine-protein kinase receptor EPH)
Human Eph A2	125333	EPHA2_HUMAN	Ephrin type-A receptor 2 precursor (Tyrosine-protein kinase receptor ECK)
Human Eph A3	125387	EPHA3_HUMAN	Ephrin type-A receptor 3 precursor (Tyrosine-protein kinase receptor ETK1)
Human Eph A4	1711371	EPHA4_HUMAN	Ephrin type-A receptor 4 precursor (Tyrosine-protein kinase receptor SEK)
Human Eph A5	1706628	EPHA5_HUMAN	Ephrin type-A receptor 5 precursor (Tyrosine-protein kinase receptor EHK-1)
Human Eph A7	68846922	EPHA7_HUMAN	Ephrin type-A receptor 7 precursor (Tyrosine-protein kinase receptor EHK-3)
Human Eph A8	19857975	EPHA8_HUMAN	Ephrin type-A receptor 8 precursor (Tyrosine-protein kinase receptor EEK)
Human Eph A10	74762207	EPHAA_HUMAN	Ephrin type-A receptor 10 precursor
Human Src	125711	SRC_HUMAN	Proto-oncogene tyrosine-protein kinase Src (p60-Src)
Human FRK	1169745	FRK_HUMAN	Tyrosine-protein kinase FRK
Human Fyn	125370	FYN_HUMAN	Proto-oncogene tyrosine-protein kinase Fyn (p59-Fyn)
Human Yes	125870	YES_HUMAN	Proto-oncogene tyrosine-protein kinase Yes (p61-Yes)
Human FGR	125358	FGR_HUMAN	Proto-oncogene tyrosine-protein kinase FGR (P55-FGR)
Human HCK	20141296	HCK_HUMAN	Tyrosine-protein kinase HCK (p59-HCK/p60-HCK)
Human LCK	125474	LCK_HUMAN	Proto-oncogene tyrosine-protein kinase LCK (p56-LCK)
Human Lyn	125480	LYN_HUMAN	Tyrosine-protein kinase Lyn
Human BLK	1705485	BLK_HUMAN	Tyrosine-protein kinase BLK (B lymphocyte kinase) (p55-BLK)
Human Srms	27805732	SRMS_HUMAN	Tyrosine-protein kinase Srms
Human ABL1	85681908	ABL1_HUMAN	Proto-oncogene tyrosine-protein kinase ABL1 (p150)
Human ABL2	1168268	ABL2_HUMAN	Tyrosine-protein kinase ABL2 (Abelson murine leukemia viral oncogene homolog 2)
Human Fes	400127	FES_HUMAN	Proto-oncogene tyrosine-protein kinase Fes/Fps
Human FER	97536202	FER_HUMAN	Proto-oncogene tyrosine-protein kinase FER (p94-FER)
Human Ror2	90110767	ROR2_HUMAN	Tyrosine-protein kinase transmembrane receptor ROR2 precursor
Human Ror1	19924290	ROR1_HUMAN	Tyrosine-protein kinase transmembrane receptor ROR1 precursor
Human NTRK1	94730402	NTRK1_HUMAN	High affinity nerve growth factor receptor precursor
Human MUSK	50400806	MUSK_HUMAN	Muscle, skeletal receptor tyrosine protein kinase precursor
Human NTRK2	2497560	NTRK2_HUMAN	BDNF/NT-3 growth factors receptor precursor
Human NTRK3	2497562	NTRK3_HUMAN	NT-3 growth factor receptor precursor
Human BTK	547759	BTK_HUMAN	Tyrosine-protein kinase BTK (Bruton tyrosine kinase)
Human Tec	1174630	TEC_HUMAN	Tyrosine-protein kinase Tec
Human ITK	585361	ITK_HUMAN	Tyrosine-protein kinase ITK/TSK (T-cell-specific kinase)
Human TXK	1351326	TXK_HUMAN	Tyrosine-protein kinase TXK
Human BMX	1705489	BMX_HUMAN	Cytoplasmic tyrosine-protein kinase BMX
Human FAK1	3183518	FAK1_HUMAN	Focal adhesion kinase 1 (FADK 1) (pp125FAK)
Human FAK2	3183003	FAK2_HUMAN	Protein tyrosine kinase 2 beta (Focal adhesion kinase 2)
Human ErbB1	2811086	EGFR_HUMAN	Epidermal growth factor receptor precursor (Receptor tyrosine-protein kinase ErbB-1)
Human ErbB3	119534	ERBB3_HUMAN	Receptor tyrosine-protein kinase erbB-3 precursor (c-erbB3)
Human ErbB4	3913590	ERBB4_HUMAN	Receptor tyrosine-protein kinase erbB-4 precursor (p180erbB4)
Human ErbB2	119533	ERBB2_HUMAN	Receptor tyrosine-protein kinase erbB-2 precursor (p185erbB2)

Table S1.18. Neuroligin family.

Abbrevietion	gi	Swissprot Locus	Description
Dicty CES-like	66802562	N.A.	hypothetical protein DDBDRAFT_0183832
Sponge CES-like	N.A.	N.A.	N.A.
CN NRL-like(1)	N.A.	N.A.	N.A.
CN NRL-like(2)	N.A.	N.A.	N.A.
CN NRL-like(3)	N.A.	N.A.	N.A.
CN NRL-like(4)	N.A.	N.A.	N.A.
CN NRL-like(5)	N.A.	N.A.	N.A.
CN NRL-like(6)	N.A.	N.A.	**N.A.**
Fly Neuroligin 1	7716610	N.A.	CG13772 neuroligin
Fly Neuroligin 2	28381150	N.A.	CG31146
Fly Gliotactin	24584488	N.A.	CG3903 Gliotactin
Fly ACES	113036	ACES_DROME	Acetylcholinesterase precursor (AChE)
Human Neuroligin 3	31076855	NLGN3_HUMAN	Neuroligin-3 precursor (Gliotactin homolog)
Human Neuroligin 4X	31076821	NLGNX_HUMAN	Neuroligin-4, X-linked precursor (Neuroligin X) (HNLX)
Human Neuroligin 4Y	31076823	NLGNY_HUMAN	Neuroligin-4, Y-linked precursor (Neuroligin Y)
Human Neuroligin 1	31076822	NLGN1_HUMAN	Neuroligin-1 precursor
Human Neuroligin 2	31076824	NLGN2_HUMAN	Neuroligin-2 precursor
Human ACES	113037	ACES_HUMAN	Acetylcholinesterase precursor (AChE)
Human CHLE	116353	CHLE_HUMAN	Cholinesterase precursor (Acylcholine acylhydrolase)
Human CEL	231629	CEL_HUMAN	Bile salt-activated lipase precursor (BAL) (Bile salt-stimulated lipase) (BSSL)
Human EST2	46576349	EST2_HUMAN	Carboxylesterase 2 precursor (CE-2) (hCE-2)
Human EST1	119576	EST1_HUMAN	Liver carboxylesterase 1 precursor

Table S1.19. Ionotropic Glutamate Receptor families.

Abbrevietion	gi	Swissprot Locus	Description
At GLR33	39545692	N.A.	GLR3.3
Os GLR	50904793	N.A.	putative glutamate receptor
CN NMDAR-like (1)	N.A.	N.A.	**N.A.**
CN NMDAR-like (2)	N.A.	N.A.	**N.A.**
CN NMDAR-like (3)	N.A.	N.A.	**N.A.**
CN NMDAR-like (4)	N.A.	N.A.	**N.A.**
CN NMDAR-like (5)	N.A.	N.A.	**N.A.**
CN NMDAR-like (6)	N.A.	N.A.	**N.A.**
CN NMDAR-like (7)	N.A.	N.A.	**N.A.**
CN NMDAR-like (8)	N.A.	N.A.	**N.A.**
CN GLR-like (1)	N.A.	N.A.	**N.A.**
CN GLR-like (2)	N.A.	N.A.	**N.A.**
CN GLR-like (3)	N.A.	N.A.	**N.A.**
Fly GLR 1	68067727	GLK1_DROME	CG8442 Glutamate receptor 1 (dGLUR-I)
Fly GLR 2	24648559	N.A.	CG3822
Fly GLR 3	61699735	N.A.	CG11155
Fly GLR 4	23171826	N.A.	CG5621
Fly GLR 5	6687415	N.A.	CG18039 KaiRIA ionotropic glutamate receptor subunit IA
Fly GLR 6	24581972	N.A.	CG6992 Glutamate receptor IIA
Fly GLR 7	24648476	N.A.	CG31201 Glutamate receptor IIE
Fly GLR 8	24661440	N.A.	CG4481 Glutamate receptor IB
Fly GLR 9	45549153	N.A.	CG7234 Glutamate receptor IIB
Fly GLR 10	22945551	N.A.	CG4226
Fly GLR 11	24581733	N.A.	CG15627
Fly GLR 12	116007354	N.A.	CG8681 clumsy
Fly GLR 13	85682857	N.A.	CG10633
Fly GLR 14	24666122	N.A.	CG14586
Fly GLR 15	24666536	N.A.	CG14076
Fly GLR 16	24667182	N.A.	CG7385
Fly GLR 17	28572148	N.A.	CG17274
Fly GLR 18	85683065	N.A.	CG32704
Fly GLR 19	24644826	N.A.	CG10101
Fly NMDAR 1	24644257	N.A.	CG2902 NMDA receptor 1
Fly NMDAR 2	48095783	N.A.	CG33513 NMDA receptor subunit 2
Human AMPAR 1	1169959	GRIA1_HUMAN	Glutamate receptor 1 precursor (GluR-1) (GluR-A) (GluR-K1) (AMPA 1)
Human AMPAR 2	23831146	GRIA2_HUMAN	Glutamate receptor 2 precursor (GluR-2) (GluR-B) (GluR-K2) (AMPA 2)
Human AMPAR 3	77416864	GRIA3_HUMAN	Glutamate receptor 3 precursor (GluR-3) (GluR-C) (GluR-K3) (AMPA 3)
Human AMPAR 4	1346142	GRIA4_HUMAN	Glutamate receptor 4 precursor (GluR-4) (GluR4) (GluR-D) (AMPA 4)
Human KainateR 1	729597	GRIK1_HUMAN	Glutamate receptor, ionotropic kainate 1 precursor (Glutamate receptor 5)
Human KainateR 2	2492627	GRIK2_HUMAN	Glutamate receptor, ionotropic kainate 2 precursor (Glutamate receptor 6)
Human KainateR 3	3287976	GRIK3_HUMAN	Glutamate receptor, ionotropic kainate 3 precursor (Glutamate receptor 7)
Human KainateR 4	3287848	GRIK4_HUMAN	Glutamate receptor, ionotropic kainate 4 precursor (Glutamate receptor KA-1)
Human KainateR 5	3287849	GRIK5_HUMAN	Glutamate receptor, ionotropic kainate 5 precursor (Glutamate receptor KA-2)
Human DeltaR 1	38372397	GRID1_HUMAN	Glutamate receptor delta-1 subunit precursor (GluR delta-1)
Human DeltaR 2	25090493	GRID2_HUMAN	Glutamate receptor delta-2 subunit precursor (GluR delta-2)
Human NMDAR 1	2343289	N.A.	NMDAR1 subunit
Human NMDAR 2A	14285603	NMDE1_HUMAN	Glutamate [NMDA] receptor subunit epsilon 1 precursor (NR2A)
Human NMDAR 2B	14548162	NMDE2_HUMAN	Glutamate [NMDA] receptor subunit epsilon 2 precursor (NR2B)
Human NMDAR 2C	2492629	NMDE3_HUMAN	Glutamate [NMDA] receptor subunit epsilon 3 precursor (NR2C)
Human NMDAR 2D	18201966	NMDE4_HUMAN	Glutamate [NMDA] receptor subunit epsilon 4 precursor (NR2D)
Human NMDAR 3A	51701684	NMD3A_HUMAN	Glutamate [NMDA] receptor subunit 3A precursor (NR3A)
Human NMDAR 3B	71153527	NMD3B_HUMAN	Glutamate [NMDA] receptor subunit 3B precursor (NR3B)

Table S1.20. K+ Channel Kir family.

Abbrevietion	gi	Swissprot Locus	Description
Sponge Kir-like 1	N.A.	N.A.	**N.A.**
Sponge Kir-like 2	N.A.	N.A.	**N.A.**
Fly Kir 1	24649234	N.A.	CG6747 Inwardly rectifying potassium channel
Fly Kir 2	24649329	N.A.	CG4370 Inwardly rectifying potassium channel 2
Fly Kir 3	62484243	N.A.	CG10369 Inwardly rectifying potassium channel 3
Human Kir 1.3	77416869	IRK15_HUMAN	Inward rectifier K(+) channel Kir4.2 (Kir1.3)
Human Kir 1.2	2493605	IRK10_HUMAN	Inward rectifier K(+) channel Kir1.2
Human Kir 1.1	1352479	IRK1_HUMAN	ATP-regulated potassium channel ROM-K (Kir1.1)
Human Kir 7.1	13878543	IRK13_HUMAN	Inward rectifier K(+) channel Kir7.1
Human Kir 2.2	77416868	IRK12_HUMAN	Inward rectifier K(+) channel Kir2.2
Human Kir 2.3	1352483	IRK4_HUMAN	Inward rectifier K(+) channel Kir2.3
Human Kir 2.1	54037433	IRK2_HUMAN	Inward rectifier K(+) channel Kir2.1
Human Kir 2.4	54036159	IRK14_HUMAN	Inward rectifier K(+) channel Kir2.4
Human Kir 5.1	13878562	IRK16_HUMAN	Inward rectifier K(+) channel Kir5.1
Human Kir 3.2	1352487	IRK6_HUMAN	Inward rectifier K(+) channel Kir3.2
Human Kir 3.4	1352484	IRK5_HUMAN	Inward rectifier K(+) channel Kir3.4
Human Kir 3.3	2493603	IRK9_HUMAN	Inward rectifier K(+) channel Kir3.3
Human Kir 3.1	1352482	IRK3_HUMAN	Inward rectifier K(+) channel Kir3.1
Human Kir 6.1	2493600	IRK8_HUMAN	Inwardly rectifier K(+) channel Kir6.1
Human Kir 6.2	76803775	IRK11_HUMAN	Inwardly rectifier K(+) channel Kir6.2

Table S1.21. K+ Channel Shaker family.

Abbrevietion	gi	Swissprot Locus	Description
CN Kv 1-like(1)	N.A.	N.A.	**N.A.**
CN Kv 1-like(2)	N.A.	N.A.	**N.A.**
CN Kv 1-like(3)	N.A.	N.A.	**N.A.**
CN Kv 3-like	N.A.	N.A.	N.A.
Fly Kv 1	13432103	KCNAS_DROME	CG12348 Potassium voltage-gated channel protein Shaker
Fly Kv 3.1	28574020	N.A.	CG2822 Shaker cognate w
Fly Kv 3.2	45445057	N.A.	CG4450
Human Kv 1.1	1168947	KCNA1_HUMAN	Voltage-gated potassium channel subunit Kv1.1
Human Kv 1.2	1345813	KCNA2_HUMAN	Voltage-gated potassium channel subunit Kv1.2
Human Kv 1.3	1168948	KCNA3_HUMAN	Voltage-gated potassium channel subunit Kv1.3
Human Kv 1.4	116430	KCNA4_HUMAN	Voltage-gated potassium channel subunit Kv1.4
Human Kv 1.5	1705863	KCNA5_HUMAN	Voltage-gated potassium channel subunit Kv1.5
Human Kv 1.6	116434	KCNA6_HUMAN	Voltage-gated potassium channel subunit Kv1.6
Human Kv 1.7	14485555	N.A.	voltage-gated potassium channel KCNA7
Human Kv 3.1	1352085	KCNC1_HUMAN	Voltage-gated potassium channel subunit Kv3.1
Human Kv 3.2	21999191	N.A.	potassium voltage-gated potassium channel subfamily C member 2
Human Kv 3.3	8488974	KCNC3_HUMAN	Voltage-gated potassium channel subunit Kv3.3
Human Kv 3.4	66774206	KCNC4_HUMAN	Voltage-gated potassium channel subunit Kv3.4

Table S1.22. Classical Cadherin family.

Abbrevietion	gi	Swissprot Locus	Description
Sponge Classical Cadherin	N.A.	N.A.	**N.A.**
Fly Dachsous	25090185	DS_DROME	CG17941 Protein dachsous precursor (Adherin)
Fly N-Cadherin	13124002	CADN_DROME	CG7100 Neural-cadherin precursor (Cadherin-N protein) (DN-cadherin)
Fly N-Cadherin2	27923751	CADN2_DROME	CG7527 Putative neural-cadherin 2 precursor (Cadherin-N2 protein) (DN2-cadherin)
Fly DE-Cadherin	13124007	CADE_DROME	CG3722 DE-cadherin precursor (Protein shotgun)
Human Dachsous	20139065	PCD16_HUMAN	Protocadherin-16 precursor (Dachsous 1) (Cadherin-19) (Fibroblast cadherin 1)
Human N-Cadherin	1705541	CADH2_HUMAN	Neural-cadherin precursor (N-cadherin) (Cadherin-2) (CDw325 antigen)
Human R-Cadherin	81175161	CADH4_HUMAN	Cadherin-4 precursor (Retinal-cadherin) (R-cadherin) (R-CAD)
Human E-Cadherin	399166	CADH1_HUMAN	Epithelial-cadherin precursor (E-cadherin) (Uvomorulin) (Cadherin-1)
Human P-Cadherin	115427	CADH3_HUMAN	Cadherin-3 precursor (Placental-cadherin) (P-cadherin)
Human M-Cadherin	1705553	CAD15_HUMAN	Muscle-cadherin precursor (M-cadherin) (Cadherin-15)
Human OB-Cadherin	1705548	CAD11_HUMAN	Cadherin-11 precursor (Osteoblast-cadherin) (OB-cadherin) (OSF-4)
Human K-Cadherin	1705545	CADH6_HUMAN	Cadherin-6 precursor (Kidney-cadherin) (K-cadherin)
Human T2-Cadherin	13431364	CAD10_HUMAN	Cadherin-10 precursor (T2-cadherin)
Human Cadherin 7	17367151	CADH7_HUMAN	Cadherin-7 precursor
Human Cadherin 9	13431362	CADH9_HUMAN	Cadherin-9 precursor
Human BR-Cadherin	1705551	CAD12_HUMAN	Cadherin-12 precursor (Brain-cadherin) (BR-cadherin) (N-cadherin 2)
Human Cadherin 18	3023435	CAD18_HUMAN	Cadherin-18 precursor (Cadherin-14)
Human Cadherin 20	17366842	CAD20_HUMAN	Cadherin-20 precursor
Human Cadherin 24	38257450	CAD24_HUMAN	Cadherin-24 precursor
Human PB-Cadherin	24211543	CAD22_HUMAN	Cadherin-22 precursor (Pituitary and brain cadherin) (PB-cadherin)
Human VE-Cadherin	13432109	CADH5_HUMAN	Cadherin-5 precursor (Vascular endothelial-cadherin) (VE-cadherin)
Human Cadherin 19	13431344	CAD17_HUMAN	Cadherin-17 precursor (Liver-intestine-cadherin) (LI-cadherin)

Table S1.23. DLG, Shank, GRIP, MAGI, LIN-7, LIMK, Mint, Tamalin, Erbin, NOS, PICK1 and CASK families.

Abbrevietion	gi	Swissprot Locus	Description
At SP PDZ			
Sponge DLG PDZ1 (AA 144 - 223)	N.A.	N.A.	**N.A.**
Sponge DLG PDZ2 (AA 274 - 353)	N.A.	N.A.	**N.A.**
Sponge DLG PDZ3 (AA 397 - 470)	N.A.	N.A.	**N.A.**
Sponge Lin7 PDZ (AA 111 - 185)	N.A.	N.A.	**N.A.**
Sponge GRIP PDZ1 (AA 35 - 109)	N.A.	N.A.	**N.A.**
Sponge GRIP PDZ2 (AA 136 - 210)	N.A.	N.A.	**N.A.**
Sponge GRIP PDZ4 (AA 278 - 354)	N.A.	N.A.	**N.A.**
Sponge GRIP PDZ5 (AA 376 - 447)	N.A.	N.A.	**N.A.**

Sponge GRIP PDZ6 (AA 475 - 549)	N.A.	N.A.	**N.A.**
Sponge GRIP PDZ7 (AA 836 - 907)	N.A.	N.A.	**N.A.**
Sponge Shank PDZ (AA 495 - 574)	N.A.	N.A.	**N.A.**
Sponge NOS PDZ (AA 16 - 88)	N.A.	N.A.	**N.A.**
Sponge PICK1 PDZ (AA 13 - 87)	N.A.	N.A.	**N.A.**
Sponge Magi PDZ1 (AA 34 -108)	N.A.	N.A.	**N.A.**
Sponge Magi PDZ2 (AA 424 - 500)	N.A.	N.A.	**N.A.**
Sponge Magi PDZ3 (AA 561 - 631)	N.A.	N.A.	**N.A.**
Sponge Magi PDZ4 (AA 678 - 754)	N.A.	N.A.	**N.A.**
Sponge Magi PDZ5 (AA 790 - 863)	N.A.	N.A.	**N.A.**
Sponge Magi PDZ6 (AA 951 - 1026)	N.A.	N.A.	**N.A.**
Sponge Mint PDZ1 (AA 364 - 442)	N.A.	N.A.	**N.A.**
Sponge Mint PDZ2 (AA 443 - 515)	N.A.	N.A.	**N.A.**
Sponge Tamalin PDZ (AA 126 - 202)	N.A.	N.A.	**N.A.**
CN Erbin-like PDZ (AA 966 - 1046)	N.A.	N.A.	**N.A.**
CN CASK PDZ (AA 625 - 721)	N.A.	N.A.	**N.A.**
Fly DLG PDZ1 (AA 224 - 303)	51704269	DLG1_DROME	CG1725 Discs large 1 tumor suppressor protein
Fly DLG PDZ2 (AA 338 - 421)	51704269	DLG1_DROME	CG1725 Discs large 1 tumor suppressor protein
Fly DLG PDZ3 (AA 514 - 587)	51704269	DLG1_DROME	CG1725 Discs large 1 tumor suppressor protein
Fly Shank PDZ (AA 688 - 773)	24653538	N.A.	CG30483 Prosap
Fly Lin7 PDZ (AA 101 - 175)	21356237	N.A.	CG7662 veli
Fly GRIP PDZ1 (AA 90 - 165)	24640023	N.A.	CG14447 Glutamate receptor binding protein
Fly GRIP PDZ2 (AA 191 - 276)	24640023	N.A.	CG14447 Glutamate receptor binding protein
Fly GRIP PDZ3 (AA 289 - 386)	24640023	N.A.	CG14447 Glutamate receptor binding protein
Fly GRIP PDZ4 (AA 475 - 543)	24640023	N.A.	CG14447 Glutamate receptor binding protein
Fly GRIP PDZ5 (AA 582 - 661)	24640023	N.A.	CG14447 Glutamate receptor binding protein
Fly GRIP PDZ6 (AA 842 - 916)	24640023	N.A.	CG14447 Glutamate receptor binding protein
Fly GRIP PDZ7 (AA 985 - 1058)	24640023	N.A.	CG14447 Glutamate receptor binding protein
Fly CASK PDZ (AA 504 - 576)	34223738	CAKI_DROME	CG6703 Calcium/calmodulin-dependent protein kinase (CaMGUK)
Fly PICK1 PDZ (AA 33 - 105)	28574146	N.A.	CG6167 PICK1
Fly Magi PDZ2 (AA 454 - 535)	20304109	N.A.	CG30388 Magi
Fly Magi PDZ3 (AA 609 - 679)	20304109	N.A.	CG30388 Magi
Fly Magi PDZ4 (AA 936 - 1012)	20304109	N.A.	CG30388 Magi
Fly Magi PDZ5 (AA 1040 - 1114)	20304109	N.A.	CG30388 Magi
Fly Mint PDZ1 (AA 995 - 1073)	24642814	N.A.	CG5675 X11L
Fly Mint PDZ2 (AA 1087 - 1153)	24642814	N.A.	CG5675 X11L
Fly LIMK PDZ (AA 186 - 274)	74865306	LIMK1_DROME	CG1848 LIM domain kinase 1 (LIMK-1) (dLIMK)
Fly Tamalin PDZ (AA 139 - 219)	45550544	N.A.	CG6619
Human SAP97 PDZ1 (AA 232- 311)	2497504	DLG1_HUMAN	Disks large homolog 1 (Synapse-associated protein 97) (SAP-97) (hDlg)
Human SAP97 PDZ2 (AA 327 - 406)	2497504	DLG1_HUMAN	Disks large homolog 1 (Synapse-associated protein 97) (SAP-97) (hDlg)
Human SAP97 PDZ3 (AA 474 - 547)	2497504	DLG1_HUMAN	Disks large homolog 1 (Synapse-associated protein 97) (SAP-97) (hDlg)
Human C110 PDZ1 (AA 106 - 185)	73920200	DLG2_HUMAN	Discs large homolog 2 (Channel-associated protein of synapse-110)
Human C110 PDZ2 (AA 201 - 280)	73920200	DLG2_HUMAN	Discs large homolog 2 (Channel-associated protein of synapse-110)
Human C110 PDZ3 (AA 429 - 502)	73920200	DLG2_HUMAN	Discs large homolog 2 (Channel-associated protein of synapse-110)
Human SAP102 PDZ1 (AA 139 - 218)	2497506	DLG3_HUMAN	Discs large homolog 3 (Synapse-associated protein 102) (SAP102)
Human SAP102 PDZ2 (AA 234 - 313)	2497506	DLG3_HUMAN	Discs large homolog 3 (Synapse-associated protein 102) (SAP102)
Human SAP102 PDZ3 (AA 394 - 467)	2497506	DLG3_HUMAN	Discs large homolog 3 (Synapse-associated protein 102) (SAP102)
Human PSD95 PDZ1 (AA 73 - 152)	71658825	DLG4_HUMAN	Discs large homolog 4 (Postsynaptic density protein 95) (PSD-95)
Human PSD95 PDZ2 (AA 168 - 247)	71658825	DLG4_HUMAN	Discs large homolog 4 (Postsynaptic density protein 95) (PSD-95)
Human PSD95 PDZ3 (AA 321 - 394)	71658825	DLG4_HUMAN	Discs large homolog 4 (Postsynaptic density protein 95) (PSD-95)
Human GRIP1 PDZ1 (AA 62 - 136)	48474711	GRIP1_HUMAN	Glutamate receptor-interacting protein 1 (GRIP1 protein)
Human GRIP1 PDZ2 (AA 160 - 238)	48474711	GRIP1_HUMAN	Glutamate receptor-interacting protein 1 (GRIP1 protein)
Human GRIP1 PDZ3 (AA 261 - 336)	48474711	GRIP1_HUMAN	Glutamate receptor-interacting protein 1 (GRIP1 protein)
Human GRIP1 PDZ4 (AA 481 - 561)	48474711	GRIP1_HUMAN	Glutamate receptor-interacting protein 1 (GRIP1 protein)
Human GRIP1 PDZ5 (AA 582 - 658)	48474711	GRIP1_HUMAN	Glutamate receptor-interacting protein 1 (GRIP1 protein)
Human GRIP1 PDZ6 (AA 681 - 755)	48474711	GRIP1_HUMAN	Glutamate receptor-interacting protein 1 (GRIP1 protein)
Human GRIP1 PDZ7 (AA 1014 - 1086)	48474711	GRIP1_HUMAN	Glutamate receptor-interacting protein 1 (GRIP1 protein)
Human GRIP2 PDZ1 (AA 57 - 131)	48474948	GRIP2_HUMAN	Glutamate receptor-interacting protein 2 (GRIP2 protein)
Human GRIP2 PDZ2 (AA 156 - 234)	48474948	GRIP2_HUMAN	Glutamate receptor-interacting protein 2 (GRIP2 protein)
Human GRIP2 PDZ3 (AA 257 - 332)	48474948	GRIP2_HUMAN	Glutamate receptor-interacting protein 2 (GRIP2 protein)
Human GRIP2 PDZ4 (AA 465 - 545)	48474948	GRIP2_HUMAN	Glutamate receptor-interacting protein 2 (GRIP2 protein)
Human GRIP2 PDZ5 (AA 566 - 641)	48474948	GRIP2_HUMAN	Glutamate receptor-interacting protein 2 (GRIP2 protein)
Human GRIP2 PDZ6 (AA 664 - 738)	48474948	GRIP2_HUMAN	Glutamate receptor-interacting protein 2 (GRIP2 protein)
Human GRIP2 PDZ7 (AA 951 - 1023)	48474948	GRIP2_HUMAN	Glutamate receptor-interacting protein 2 (GRIP2 protein)
Human Shank1 PDZ (AA 672 - 761)	22001988	SHAN1_HUMAN	SH3 and multiple ankyrin repeat domains protein 1 (Shank1)
Human Shank2 PDZ (AA 46 - 131)	22001987	SHAN2_HUMAN	SH3 and multiple ankyrin repeat domains protein 2 (Shank2)
Human Lin7A PDZ (AA 116 - 190)	59798442	LIN7A_HUMAN	LIN-7 homolog A (LIN-7A) (hLin-7) (Mammalian LIN-seven protein 1)
Human Lin7B PDZ (AA 101 - 175)	59798472	LIN7B_HUMAN	LIN-7 homolog B (LIN-7B) (hLin7B) (Mammalian LIN-seven protein 2) (MALS-2)
Human Lin7C PDZ (AA 101 - 175)	59798474	LIN7C_HUMAN	LIN-7 homolog C (LIN-7C) (Mammalian LIN-seven protein 3) (MALS-3)
Human Erbin PDZ (AA 1330 - 1410)	50401131	LAP2_HUMAN	LAP2 protein (Erbb2-interacting protein) (Erbin) (Densin-180-like protein)
Human Densin_180 PDZ (AA 1455 - 1535)	50401129	LRRC7_HUMAN	Leucine-rich repeat-containing protein 7 (LAP1 protein) (Densin-180)
Human PICK1 PDZ (AA 31 - 105)	22095990	PICK1_HUMAN	PRKCA-binding protein (Protein interacting with C kinase 1)
Human CASK PDZ (AA 499 - 571)	27735175	CSKP_HUMAN	Peripheral plasma membrane protein CASK (hCASK)
Human Magi1 PDZ1 (AA 26 -105)	52782748	MAGI1_HUMAN	Membrane-associated guanylate kinase, WW and PDZ domain-containing protein 1
Human Magi1 PDZ2 (AA 480 - 556)	52782748	MAGI1_HUMAN	Membrane-associated guanylate kinase, WW and PDZ domain-containing protein 1
Human Magi1 PDZ3 (AA 651 - 753)	52782748	MAGI1_HUMAN	Membrane-associated guanylate kinase, WW and PDZ domain-containing protein 1
Human Magi1 PDZ4 (AA 849 - 925)	52782748	MAGI1_HUMAN	Membrane-associated guanylate kinase, WW and PDZ domain-containing protein 1
Human Magi1 PDZ5 (AA 1007 - 1094)	52782748	MAGI1_HUMAN	Membrane-associated guanylate kinase, WW and PDZ domain-containing protein 1
Human Magi1 PDZ6 (AA 1160 - 1234)	52782748	MAGI1_HUMAN	Membrane-associated guanylate kinase, WW and PDZ domain-containing protein 1
Human Magi2 PDZ1 (AA 26 - 101)	88909269	MAGI2_HUMAN	Membrane-associated guanylate kinase, WW and PDZ domain-containing protein 2
Human Magi2 PDZ2 (AA 434 - 510)	88909269	MAGI2_HUMAN	Membrane-associated guanylate kinase, WW and PDZ domain-containing protein 2

Human Magi2 PDZ3 (AA 613 - 683)	88909269	MAGI2_HUMAN	Membrane-associated guanylate kinase, WW and PDZ domain-containing protein 2
Human Magi2 PDZ4 (AA 786 - 862)	88909269	MAGI2_HUMAN	Membrane-associated guanylate kinase, WW and PDZ domain-containing protein 2
Human Magi2 PDZ5 (AA 929 - 1010)	88909269	MAGI2_HUMAN	Membrane-associated guanylate kinase, WW and PDZ domain-containing protein 2
Human Magi2 PDZ6 (AA 1155 - 1229)	88909269	MAGI2_HUMAN	Membrane-associated guanylate kinase, WW and PDZ domain-containing protein 2
Human Magi3 PDZ1 (AA 27 - 106)	27544925	N.A.	membrane-associated guanylate kinase-related 3 isoform 1
Human Magi3 PDZ2 (AA 443 - 519)	27544925	N.A.	membrane-associated guanylate kinase-related 3 isoform 1
Human Magi3 PDZ3 (AA 611 - 681)	27544925	N.A.	membrane-associated guanylate kinase-related 3 isoform 1
Human Magi3 PDZ4 (AA 759 - 835)	27544925	N.A.	membrane-associated guanylate kinase-related 3 isoform 1
Human Magi3 PDZ5 (AA 885 - 963)	27544925	N.A.	membrane-associated guanylate kinase-related 3 isoform 1
Human Magi3 PDZ6 (AA 1054 - 1128)	27544925	N.A.	membrane-associated guanylate kinase-related 3 isoform 1
Human Mint1 PDZ1 (AA 665 - 743)	6226838	APBA1_HUMAN	Amyloid beta A4 precursor protein-binding family A member 1 (Mint-1)
Human Mint1 PDZ2 (AA 757 - 823)	6226838	APBA1_HUMAN	Amyloid beta A4 precursor protein-binding family A member 1 (Mint-1)
Human Mint2 PDZ1 (AA 577 - 655)	6226950	APBA2_HUMAN	Amyloid beta A4 precursor protein-binding family A member 2 (Mint-2)
Human Mint2 PDZ2 (AA 669 - 735)	6226950	APBA2_HUMAN	Amyloid beta A4 precursor protein-binding family A member 2 (Mint-2)
Human Mint3 PDZ1 (AA 403 - 481)	6226953	APBA3_HUMAN	Amyloid beta A4 precursor protein-binding family A member 3 (Mint-3)
Human Mint3 PDZ2 (AA 495 - 561)	6226953	APBA3_HUMAN	Amyloid beta A4 precursor protein-binding family A member 3 (Mint-3)
Human nNOS PDZ (AA 26 - 100)	1709333	NOS1_HUMAN	Nitric-oxide synthase, brain (NOS type I) (Neuronal NOS) (N-NOS) (nNOS)
Human LIMK1 PDZ (AA 176 - 258)	90185240	LIMK1_HUMAN	LIM domain kinase 1 (LIMK-1)
Human LIMK2 PDZ (AA 161 - 239)	1708824	LIMK2_HUMAN	LIM domain kinase 2 (LIMK-2)
Human Tamalin 1 PDZ (AA 110 - 190)	74738818	GRASP_HUMAN	Tamalin - General receptor for phosphoinositides 1-associated scaffold protein
