# Supplementary material for: A Post-Synaptic Scaffold at the Origin of the Animal Kingdom
Source: PLoS One. 2007 Jun 6;2(6):e506. doi: 10.1371/journal.pone.0000506 (PMC1876816; doi:10.1371/journal.pone.0000506)
Supplement: Table S2 — Sponge dlg and Human SAP97 intron/exon structure comparison. Cloned cDNAs of Sponge (Amphimedon) dlg were fully sequenced and mapped to its genomic sequence. Intron/exon structure was compared with its human ortholog SAP97, and about half the exons were found to be almost same size (highlighted in red) and content (in bold). However, human SAP-97 introns were in average 100 times larger than Amphimedon dlg introns (data not shown). (0.03 MB PDF) [file pone.0000506.s008.pdf]

	**Sponge *dlg***	Spanning Domains	**Human SAP97**	Spanning Domains
exon1			159	
exon2	39		50	
**exon3**	**135**	**L27**	**132**	**L27**
**exon4**	**165**	**L27 + gap**	**167**	**L27 + gap**
exon5			165	
exon6	32		99	
exon7	39		54	
exon8	34		51	
**exon9**	**128**	**PDZ1**	**125**	**PDZ1**
exon10	**102**	**PDZ1**	**170**	**PDZ1**
exon11	**203**	**PDZ1 + gap + PDZ2**	**137**	**PDZ2**
exon12	**245**	**PDZ2**	**145**	**PDZ2**
exon13	**213**	**gap + PDZ3**	**121**	**gap**
exon14			**157**	**PDZ3**
**exon15**	**102**	**PDZ3**	**103**	**PDZ3**
exon16	**68**		**115**	
**exon17**	**176**	**SH3**	**177**	**SH3**
**exon18**	**64**		**67**	
exon19	124		100	
exon20	46		42	
exon21			51	
**exon22**	**101**		**102**	
**exon23**	**173**	**GK**	**173**	**GK**
**exon24**	**112**	**GK**	**110**	**GK**
**exon25**	**90**	**GK**	**92**	**GK**
**exon26**	**107**		**107**	
