# Supplementary material for: A Post-Synaptic Scaffold at the Origin of the Animal Kingdom
Source: PLoS One. 2007 Jun 6;2(6):e506. doi: 10.1371/journal.pone.0000506 (PMC1876816; doi:10.1371/journal.pone.0000506)
Supplement: Table S3 — Presence of post-synaptic gene orthologs in animals, yeast, dicty and plants. Species abbreviations used: Human, Homo sapiens; Fly, Drosophila Melanogaster; Nema, Nematostella vectensis; Sponge, Amphimedon queenslandica; Yeast, Saccharomyces cerevisiae; Dicty, Dictyostelium discoideum; Plants, Arabidopsis thaliana and Oryza sativa. (0.08 MB PDF) [file pone.0000506.s009.pdf]

	Human	Fly	Nema	Sponge	Yeast	Dicty	Plants
**PKC**	YES	YES	YES	YES	YES	YES	YES
**PMCA**	YES	YES	YES	YES	YES	YES	YES
**CRIPT**	YES	YES	YES	YES	NO	NO	YES
**iGluR**	YES	YES	YES	NO	NO	NO	YES
**mGluR**	YES	YES	YES	YES	NO	NO	NO
**GABABR**	YES	YES	YES	YES	NO	YES	NO
**K+ Kir Channel**	YES	YES	YES	YES	NO	NO	NO
**K+ Shaker**	YES	YES	YES	NO	NO	NO	NO
**Neuroligin**	YES	YES	YES	NO	NO	NO	NO
**C. Cadherin**	YES	YES	YES	YES	NO	NO	NO
**Homer**	YES	YES	YES	YES	NO	NO	NO
**NOS**	YES	YES	YES	YES	NO	NO	NO
**IP3R**	YES	YES	YES	YES	NO	NO	NO
**Stargazin**	YES	YES	NO	NO	NO	NO	NO
**GKAP/DLG7**	YES	YES	YES	YES	NO	NO	NO
**Cortactin**	YES	YES	YES	YES	NO	NO	NO
**SPAR**	YES	YES	YES	YES	NO	NO	NO
**SynGAP**	YES	YES	YES	YES	NO	NO	NO
**Citron**	YES	YES	YES	YES	NO	NO	NO
**δ-Catenin**	YES	YES	YES	YES	NO	NO	NO
**β-Catenin**	YES	YES	YES	YES	NO	NO	NO
**α-Catenin**	YES	YES	YES	YES	NO	NO	NO
**CaMKII**	YES	YES	YES	YES	NO	NO	NO
**DLG**	YES	YES	YES	YES	NO	NO	NO
**CASK**	YES	YES	YES	NO	NO	NO	NO
**PICK1**	YES	YES	YES	YES	NO	NO	NO
**Mint (LIN-10)**	YES	YES	YES	YES	NO	NO	NO
**LIN-7 (Veli)**	YES	YES	YES	YES	NO	NO	NO
**LimK**	YES	YES	NO	NO	NO	NO	NO
**Erbin**	YES	YES	YES	NO	NO	NO	NO
**Tamalin**	YES	YES	YES	YES	NO	NO	NO
**GRIP**	YES	YES	NO	YES	NO	NO	NO
**Shank**	YES	YES	YES	YES	NO	NO	NO
**MAGI**	YES	YES	YES	YES	NO	NO	NO
**Ephrin Receptor**	YES	YES	YES	YES	NO	NO	NO
**ErbB Receptor**	YES	YES	YES	YES	NO	NO	NO
