# Supplementary material for: A Post-Synaptic Scaffold at the Origin of the Animal Kingdom
Source: PLoS One. 2007 Jun 6;2(6):e506. doi: 10.1371/journal.pone.0000506 (PMC1876816; doi:10.1371/journal.pone.0000506)
Supplement: Figure S4 — Structural interactions between the C-terminus of the CRIPT peptide and the PDZ3 domain. (A), the C-terminus of the CRIPT peptide and the PDZ3 domain in rat, fish, fly, mosquito, cnidarian, sea urchin, and worm and (B), the C-terminus of the CRIPT peptide and the PDZ-3 domain in sponge. All the distances shown in part A and the “ray” marks indicating hydrophobic contacts are based on the rat (1BE9) crystal structure. Distances to Lys5 are shown to the beta-carbon because this is the last atom whose coordinates were reported in the crystal structure. Hydrophobic contacts from residues G322, A376, and L379 were found to interact in some organisms, but in the rat they were somewhat distant. For each residue, the main label corresponds to the rat sequence, and the smaller (or “residue name”) notes correspond to other organisms (not rat or sponge). Two interactions seen in the worm homology model were not included because they were unlike those seen in other organisms (G330 and P335). In the sponge diagram (part B), interactions that are possible given homology to the rat sequence are indicated, such as the hydrogen-bonds between PDZ3 and peptide. Residue numbering in the sponge structure corresponds to the aligned residue in the rat sequence. Some of the homology models suggested variant hydrogen-bonding patterns in the sponge, especially for residues Gln6 and Asn326. The figure was created using Ligplot and HBPLUS. The identical residues between rat and sponge are: 311, 312, 314, 318, 322*, 323*, 324*, 325*, 326*, 327*, 328*, 329, 330, 331, 335, 336, 337, 338, 339*, 341, 345, 347, 351, 353, 354, 356, 357, 359, 360, 362, 363, 364, 367, 371, 372*, 373, 375, 376, 378, 379, 380*, 382, 383, 385, 386, 387, 392, 393, 394, 396, and 400. Residues that make direct contact with the CRIPT ligand in the rat crystal structure are marked with an asterisk. (0.13 MB PDF) [file pone.0000506.s004.pdf]

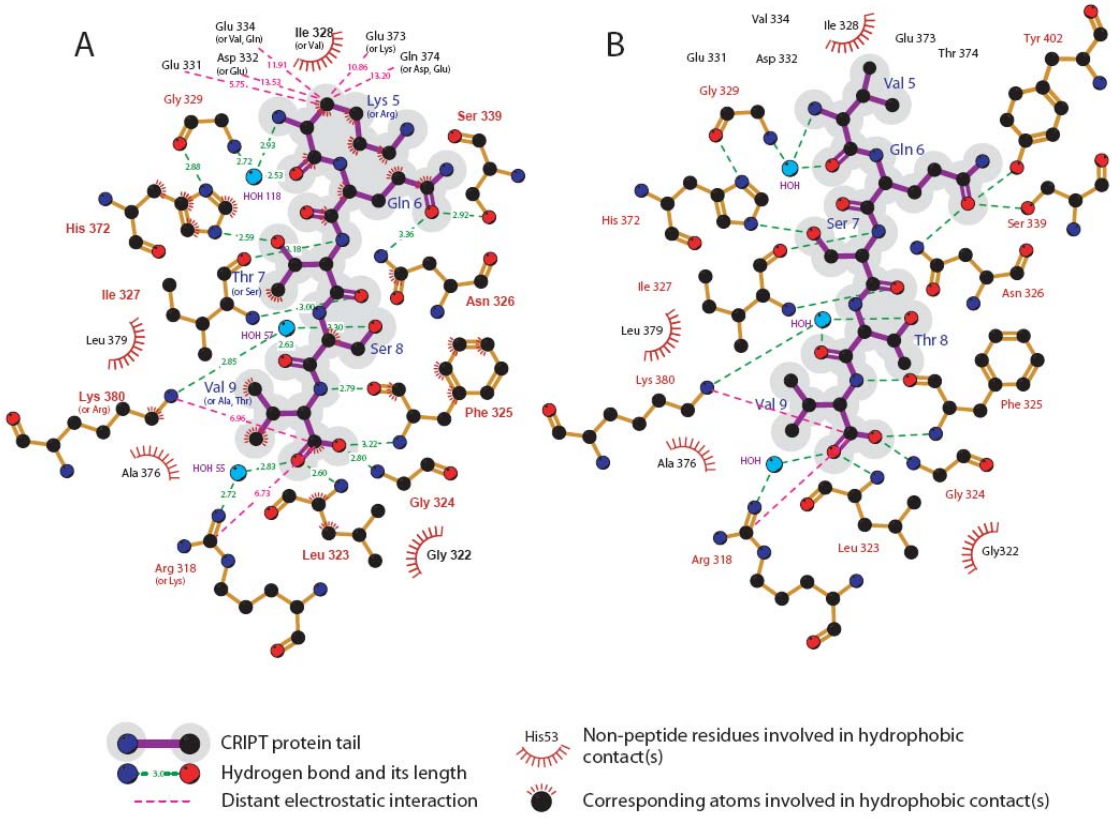

A
Glu 334
(or Val, Gln)
Ile 328
(or Val)
Glu 373
(or Lys)
Gln 374
(or Asp, Glu)
Asp 332
(or Glu)
Glu 331
Gly 329
Lys 5
(or Arg)
Ser 339
HOH 118
His 372
Gln 6
Thr 7
(or Ser)
Ile 327
Asn 326
Leu 379
HOH 57
Ser 8
Lys 380
(or Arg)
Val 9
(or Ala, Thr)
Phe 325
Ala 376
HOH 55
Gly 324
Leu 323
Gly 322
Arg 318
(or Lys)
B
Val 334
Ile 328
Glu 373
Thr 374
Glu 331
Asp 332
Tyr 402
Val 5
Gly 329
Gln 6
HOH
His 372
Ser 7
Ser 339
Asn 326
Ile 327
HOH
Leu 379
Thr 8
Lys 380
Val 9
Phe 325
Ala 376
HOH
Gly 324
Arg 318
Leu 323
Gly322
CRIPT protein tail
Hydrogen bond and its length
Distant electrostatic interaction
His53
Non-peptide residues involved in hydrophobic contact(s)
Corresponding atoms involved in hydrophobic contact(s)
